# Supplementary material for: Hydrogen Bonding Drives Helical Chirality via 10-Membered Rings in Dipeptide Conjugates of Ferrocene-1,1′-Diamine
Source: Int J Mol Sci. 2022 Oct 13;23(20):12233. doi: 10.3390/ijms232012233 (PMC9603039; doi:10.3390/ijms232012233)
Supplement: Supplementary file 1 [file ijms-23-12233-s001.zip › ijms-1931729-supplementary.pdf]

Hydrogen bonding drives helical chirality via 10-membered rings in dipeptide conjugates of ferrocene-1,1’-diamine

Monika Kovačević, Dora Markulin, Matea Zelenika, Marko Marjanović, Marija Lovrić, Denis Polančec, Marina Ivančić, Jasna Mrvčić, Krešimir Molčanov, Valentina Milašinović, Sunčica Roca, Ivan Kodrin and Lidija Barišić

Supplementary Material

Content:

Synthesis of precursors **2–31** 2

DFT data of the goal compounds **32–37** 7

MS, NMR and IR data of the goal compounds **32–37** 16

Ac–L–Phe–NH–Fn–NH–L–Phe–Boc **(32)** 16

Ac–D–Phe–NH–Fn–NH–D–Phe–Boc **(35)** 30

Ac–L–Val–NH–Fn–NH–L–Val–Boc **(33)** 33

Ac–D–Val–NH–Fn–NH–D–Val–Boc **(36)** 47

Ac–L–Leu–NH–Fn–NH–L–Leu–Boc **(34)** 50

Ac–D–Leu–NH–Fn–NH–D–Leu–Boc **(37)** 64

X-ray crystal structure analysis of **33** and **36** 69

Biological evaluation of the goal compounds **32–37** 70

Synthesis of precursors 2–31

Synthesis of Boc–L–AA–NH–Fn–COOMe (2–7): Boc–NH–Fn–COOMe (**1**) (1000 mg, 2.78 mmol) was Boc-deprotected in the presence of HCl$_{\text{gas}}$ in dry CH_2Cl_2 (5 ml) at 0 °C. After 2 hours, the solvent was evaporated *in vacuo* to afford the hydrochloride salt which was then suspended in CH_2Cl_2 and treated with NEt_3 (pH ~ 8) to give free amine suitable for coupling to Boc–L-AA–OH or Boc–D-AA–OH (5.57 mmol) (AA = Phe, Val and Leu) using the standard EDC/ HOBt method [EDC (2135 mg, 11.1 mmol); HOBt (1505 mg, 11.1 mmol)]. The reaction mixtures were then stirred at room temperature until total consumption of ferrocene amine, which was monitored by TLC (~ 1-3 hour). Standard work-up (washing with a saturated aqueous solution of $NaHCO_3$, a 10% aqueous solution of citric acid and brine, drying over Na_2SO_4 and evaporation in vacuo) including TLC purification of the crude products [EtOAc : CH_2Cl_2 = 1 : 5; R_f = 0.75 **(2)**, R_f = 0.63 **(3)**, R_f = 0.83 **(4)**, R_f = 0.72 **(5)**, R_f = 0.66 **(6)**, R_f = 0.80 **(7)**] gave orange solids of **2** (1053 mg, 75%), **3** (815 mg, 64%), **4** (850 mg, 65%), **5** (982 mg, 70%), **6** (764 mg, 60%) and **7** (771 mg, 59%).

Boc–L–Phe–NH–Fn–COOMe (2): **IR** (CH_2Cl_2) ν_{max}/cm^{-1}: 3416 m (NH$_{\text{free}}$), 3324 w (NH$_{\text{assoc.}}$), 1709 s (C=O$_{\text{COOMe}}$), 1685 s (C=O$_{\text{Boc}}$), 1640 m (C=O$_{\text{CONH}}$), 1535 m, 1496 m, 1466 m (amide II). **^{1}H-NMR** (600 MHz, $CDCl_3$) *δ*: 7.34-7.24 (m, 5H, CH$_{\text{Phe}}$), 7.32 (s, 1H, NH$_{\text{Fn}}$), 5.15 (s, 1H, NH$_{\text{Boc}}$), 4.72 (s, 1H, CH$_{\text{Fn}}$), 4.68 (s, 1H, CH$_{\text{Fn}}$), 4.61 (s, 1H, CH$_{\text{Fn}}$), 4.44 (s, 1H, CH$_{\text{Fn}}$), 4.39 (d, *J* = 6.6 Hz, 1H, CH$_{\alpha\text{-Phe}}$), 4.32 (s, 2H, CH$_{\text{Fn}}$), 4.02 (s, 1H, CH$_{\text{Fn}}$), 4.00 (s, 1H, H$_{\text{Fn}}$), 3.78 (s, 3H, CH$_{3\text{-COOMe}}$), 3.17 (dd, *J*= 6.1, 13.1 Hz, 1H, CH$_{\beta1\text{-Phe}}$), 3.09 (dd, *J* = 7.1, 13.5 Hz, 1H, CH$_{\beta2\text{-Phe}}$), 1.42 [s, 9H, (CH$_3$)$_{3\text{-Boc}}$] ppm. **^{13}C-NMR** (150 MHz, $CDCl_3$) *δ*: 171.84 (1C, CO$^{a}_{\text{Fn}}$), 169.93 (1C, CO$^{b}_{\text{Fn}}$), 155.66 (1C, CO$_{\text{Boc}}$), 136.92 (1C, C$_{q\text{-}\gamma\text{Phe}}$), 129.53 (2C, CH$_{\text{Phe}}$), 128.87 (2C, CH$_{\text{Phe}}$), 127.14 (1C, CH$_{\text{Phe}}$), 94.51 (1C, C$_q-1_{\text{Fn}}$), 80.62 (1C, C$_{q\text{Boc}}$), 72.76 (1C, CH$_{\text{Fn}}$), 72.05 (1C, C$_q-6_{\text{Fn}}$), 71.36, 71.32, 66.79, 66.57, 63.74, 63.25 (7C, CH$_{\text{Fn}}$), 56.38 (1C, CH$_{\square\text{-Phe}}$), 51.81 (1C, CH$_{3\text{-COOMe}}$), 38.37 (1C, CH$_{2\beta\text{-Phe}}$), 28.43 [3C, (CH$_3$)$_{3\text{-Boc}}$] ppm.

Boc–L–Val–NH–Fn–COOMe (3): **IR** (CH_2Cl_2) ν_{max}/cm^{-1}: 3422 m (NH$_{\text{free}}$), 3324 w (NH$_{\text{assoc.}}$), 1710 s (C=O$_{\text{COOMe}}$), 1683 s (C=O$_{\text{Boc}}$), 1636 m (C=O$_{\text{CONH}}$), 1534 m, 1498 m, 1466 m (amide II). **^{1}H-NMR** (600 MHz, $CDCl_3$) *δ*: 7.35 (s, 1H, NH$_{\text{Fn}}$), 5.14 (s, 1H, NH$_{\text{Boc}}$), 4.75 (s, 2H, CH$_{\text{Fn}}$), 4.65 (s, 1H, CH$_{\text{Fn}}$), 4.54 (s, 1H, CH$_{\text{Fn}}$), 4.39 (s, 2H, CH$_{\text{Fn}}$), 4.04 (s, 2H, CH$_{\text{Fn}}$), 3.94 (t, *J* = 6.8 Hz, 1H, CH$_{\alpha\text{–Val}}$), 3.81 (s, 3H, CH$_{3\text{-COOMe}}$), 2.22 (s, 1H, CH$_{\beta\text{-Val}}$), 1.47 [s, 9H, (CH$_3$)$_{3\text{-Boc}}$], 1.02 [d, *J* = 6.7 Hz, 3H, (CH$_3$)$_{\gamma\text{-aVal}}$], 0.98 [d, *J* = 6.7 Hz, 3H, (CH$_3$)$_{\gamma\text{-bVal}}$] ppm. **^{13}C-NMR** (150 MHz, $CDCl_3$) *δ*: 172.04 (1C, CO$^{a}_{\text{Fn}}$), 170.39 (1C, CO$^{b}_{\text{Fn}}$), 156.25 (1C, CO$_{\text{Boc}}$), 94.75 (1C, C$_q$-1$_{\text{Fn}}$), 80.26 (1C, C$_{q\text{Boc}}$), 72.77 (1C, CH$_{\text{Fn}}$), 72.00 (1C, C$_q$-6$_{\text{Fn}}$), 71.42, 71.34, 66.80, 66.60, 63.70 (7C, CH$_{\text{Fn}}$), 63.36 (1C, CH$_{\alpha\text{-Val}}$), 51.85 (1C, CH$_{3\text{-COOMe}}$), 30.65 (1C, CH$_{\beta\text{-Val}}$), 28.51 [3C, (CH$_3$)$_{3\text{-Boc}}$], 19.59 [1C, (CH$_3$)$_{\gamma\text{-aVal}}$], 17.94 [1C, (CH$_3$)$_{\gamma\text{-bVal}}$] ppm.

Boc–L–Leu–NH–Fn–COOMe (4): **IR** (CH_2Cl_2) ν_{max}/cm^{-1}: 3425 m (NH$_{\text{free}}$), 3320 w (NH$_{\text{assoc.}}$), 1708 s (C=O$_{\text{COOMe}}$), 1685 s (C=O$_{\text{Boc}}$), 1654 m (C=O$_{\text{CONH}}$), 1534 m, 1499 m, 1467 m (amide II). **^{1}H-NMR** (600 MHz, $CDCl_3$) *δ*: 7.57 (s, 1H, NH$_{\text{Fn}}$), 5.01 (d, *J* = 8.1 Hz, 1H, NH$_{\text{Boc}}$), 4.76 (pt, 2H, CH$_{\text{Fn}}$), 4.68 (s, 1H, CH$_{\text{Fn}}$), 4.55 (s, 1H, CH$_{\text{Fn}}$), 4.39 (pt, 2H, CH$_{\text{Fn}}$), 4.16-4.11 (m, 1H, CH$_{\alpha\text{-Leu}}$), 4.04 (s, 1H, CH$_{\text{Fn}}$), 4.01 (s, 1H, CH$_{\text{Fn}}$), 3.81 (s, 3H, CH$_{3\text{-COOMe}}$), 1.79-1.71 (m, 2H, CH$_{2\text{-}\beta1\text{-Leu}}$, CH$_{\gamma\text{-Leu}}$), 1.56-1.51 (m, 1H, CH$_{2\text{-}\beta2\text{-Leu}}$), 1.48 [s, 9H, (CH$_3$)$_{3\text{-Boc}}$], 0.97 [t, *J*= 6.7 Hz, 6H, (CH$_3$)$_{2\text{-}\delta\text{-Leu}}$] ppm. **^{13}C-NMR** (150 MHz, $CDCl_3$) *δ*: 171.92 (1C, CO$^{a}_{\text{Fn}}$), 171.11 (1C, CO$^{b}_{\text{Fn}}$), 156.22 (1C, CO$_{\text{Boc}}$), 95.17 (1C, C$_q$-1$_{\text{Fn}}$), 80.63 (1C, C$_{q\text{Boc}}$),

72.81, 72.78 (2C, CH_{Fn}), 72.29 (1C, C_q-6_{Fn}), 72.03, 71.39, 71.27, 66.76, 66.46 (5C, CH_{Fn}), 63.43 ($CH_{\alpha\text{-}Leu}$), 63.06 (1C, CH_{Fn}), 51.82 (1C, $CH_{3\text{-}COOMe}$), 40.91 [1C, $(CH_2)_{\beta\text{-}Leu}$], 28.52 [3C, $(CH_3)_{3\text{-}Boc}$], 24.95 (1C, $CH_{\gamma\text{-}Leu}$), 23.14, 22.15 [2C, $(CH_3)_2$-$_{\delta\text{-}Leu}$] ppm.

Boc–D–Phe–NH–Fn–COOMe (5): **IR** (CH_2Cl_2) ν_{max}/cm^{-1}: 3412 m (NH_{free}), 3323 w ($NH_{assoc.}$), 1709 s ($C{=}O_{COOMe}$), 1687 s ($C{=}O_{Boc}$), 1605 m ($C{=}O_{CONH}$), 1557 m, 1536 m, 1508 m, 1496 m (amide II). **^{1}H-NMR** (600 MHz, $CDCl_3$) δ: 7.34-7.31 (m, 5H, CH_{Phe}), 7.27 (s, 1H, NH_{Fn}), 5.15 (s, 1H, NH_{Boc}), 4.71 (s, 1H, CH_{Fn}), 4.68 (m, 1H, CH_{Fn}), 4.62 (pt, 1H, CH_{Fn}), 4.43 (s, 1H, CH_{Fn}), 4.40-4.39 (m, 1H, $CH_{\alpha\text{-}Phe}$), 4.32 (pt, 2H, CH_{Fn}), 4.03-4.01 (m, 2H, CH_{Fn}), 3.78 (s, 3H, $CH_{3\text{-}COOMe}$), 3.17 (m, 1H, $CH_{\beta1\text{-}Phe}$), 3.10-3.06 (m, 1H, $CH_{\beta2\text{-}Phe}$), 1.42 [s, 9H, $(CH_3)_{3\text{-}Boc}$] ppm. **^{13}C-NMR** (150 MHz, $CDCl_3$) δ: 171.90 (1C, CO^a_{Fn}), 169.93 (1C, CO^b_{Fn}), 155.76 (1C, CO_{Boc}), 136.89 (1C, $C_{q\text{-}\square Phe}$), 129.53 (2C, CH_{Phe}), 128.88 (2C, CH_{Phe}), 127.14 (1C, CH_{Phe}), 94.46 (1C, C_q-1_{Fn}), 80.59 (1C, C_{qBoc}), 72.76 (1C, CH_{Fn}), 72.02 (1C, C_q-6_{Fn}), 71.35, 71.31, 66.80, 66.57, 63.73, 63.25 (7C, CH_{Fn}), 56.36 (1C, $CH_{\alpha\text{-}Phe}$), 51.84 (1C, $CH_{3\text{-}COOMe}$), 38.34 (1C, $CH_{2\beta\text{-}Phe}$), 28.42 [3C, $(CH_3)_{3\text{-}Boc}$] ppm.

Boc–D–Val–NH–Fn–COOMe (6): IR (CH_2Cl_2) ν_{max}/cm^{-1}: 3425 s (NH_{free}), 3323 m ($NH_{assoc.}$), 1709 s ($C{=}O_{COOMe}$), 1688 s ($C{=}O_{CONH}$), 1534 s, 1498 s (amide II). **^{1}H-NMR** (600 MHz, $CDCl_3$) δ: 7.48 (s, 1H, NH_{Fn}), 5.20 (d, J = 8.55 Hz, 1H, NH_{Boc}), 4.74 (pt, 2H, CH_{Fn}), 4.65 (s, 1H, CH_{Fn}), 4.55 (s, 1H, CH_{Fn}), 4.38 (pt, 2H, CH_{Fn}), 4.03 (pt, 2H, CH_{Fn}), 3.96-3.93 (m, 1H, $CH_{\alpha\text{-}Val}$), 3.80 (s, 3H, $CH_{3\text{-}COOMe}$), 2.19 (s, 1H, $CH_{\beta\text{-}Val}$), 1.46 (s, 9H, $(CH_3)_{3\text{-}Boc}$), 1.02 (d, J = 6.8 Hz, 3H, $(CH_3)_{\gamma\text{-}aVal}$), 0.98 (d, J = 6.9 Hz, 3H, $(CH_3)_{\gamma\text{-}bVal}$) ppm. **^{13}C-NMR** (150 MHz, $CDCl_3$) δ: 172.06 (1C, CO^a_{Fn}), 170.44 (1C, CO^b_{Fn}), 156.21 (1C, CO_{Boc}), 94.83 (1C, C_q-1_{Fn}), 80.28 (1C, C_{qBoc}), 72.77 (1C, CH_{Fn}), 71.91 (1C, C_q-6_{Fn}), 71.34, 71.27, 66.75, 66.56, 63.54, 63.27 (7C, CH_{Fn}), 60.62 (1C, $CH_{\alpha\text{-}Val}$), 51.85 (1C, $CH_{3\text{-}COOMe}$), 30.71 (1C, $CH_{\beta\text{-}Val}$), 28.49 [3C, $(CH_3)_{3\text{-}Boc}$], 19.57 [1C, $(CH_3)_{\gamma\text{-}aVal}$], 17.98 [1C, $(CH_3)_{\gamma\text{-}bVal}$] ppm.

Boc–D–Leu–NH–Fn–COOMe (7): **IR** (CH_2Cl_2) ν_{max}/cm^{-1}: 3416 s (NH_{free}), 3322 m ($NH_{assoc.}$), 1709 s ($C{=}O_{COOMe}$), 1685 s ($C{=}O_{Boc}$), 1603 m ($C{=}O_{CONH}$), 1584 s, 1534 m, 1506 m, 1499 m (amide II). **^{1}H-NMR** (600 MHz, $CDCl_3$) δ: 7.62 (s, 1H, NH_{Fn}), 5.03 (d, J = 8.03 Hz, 1H, NH_{Boc}), 4.74 (pt, 2H, CH_{Fn}), 4.67 (s, 1H, CH_{Fn}), 4.55 (s, 1H, CH_{Fn}), 4.37 (pt, 2H, CH_{Fn}), 4.15-4.12 (m, 1H, $CH_{\alpha\text{-}Leu}$), 4.03 (s, 1H, CH_{Fn}), 4.01 (s, 1H, CH_{Fn}), 3.80 (s, 3H, $CH_{3\text{-}COOMe}$), 1.78-1.70 (m, 2H, $CH_{2\text{-}\beta1\text{-}Leu}$, $CH_{\gamma\text{-}Leu}$), 1.55-1.51 (m, 1H, $CH_{2\text{-}\beta2\text{-}Leu}$), 1.47 [s, 9H, $(CH_3)_{3\text{-}Boc}$], 0.97 [t, J= 6.3 Hz, 6H, $(CH_3)_2$-$_{\delta\text{-}Leu}$] ppm. **^{13}C-NMR** (150 MHz, $CDCl_3$) δ: 171.96 (1C, CO^a_{Fn}), 171.12 (1C, CO^b_{Fn}), 154.34 (1C, CO_{Boc}), 100.13 (1C, C_{qBoc}), 95.17 (1C, C_q-1_{Fn}), 72.81 (1C, CH_{Fn}), 72.79 (1C, C_q-6_{Fn}), 71.95, 71.35, 71.24, 66.73, 66.44 (6C, CH_{Fn}), 63.37 (1C, $CH_{\alpha\text{-}Leu}$), 63.01 (1C, CH_{Fn}), 51.83 (1C, $CH_{3\text{-}COOMe}$), 40.90 [1C, $(CH_2)_{\beta\text{-}Leu}$], 28.49 [3C, $(CH_3)_{3\text{-}Boc}$], 24.91 (1C, $CH_{\gamma\text{-}Leu}$), 23.13, 22.15 [2C, $(CH_3)_2$-$_{\delta\text{-}Leu}$] ppm.

Synthesis of Ac–L–AA–NH–Fn–COOMe (8–13): The transformation of carbamates **2–7** (2 mmol) to acetamides **8–13** began with the acidic Boc-deprotection described above. Their free amines, obtained by treating the hydrochloride salt with NEt_3 (25.1 mmol), were cooled to 0°C and acetyl chloride (12 mmol) was added dropwise, stirring in an ice bath. After TLC monitoring showed complete conversion of the starting materials, the reaction mixtures were poured into water and extracted with CH_2Cl_2. The combined organic phases were washed with a brine, dried over Na_2SO_4 and evaporated to dryness in vacuo. The resulting crude products were purified by TLC on silica gel [EtOAc : CH_2Cl_2 = 1 : 5; R_f =

0.25 (**8**), R_f = 0.16 (**9**), R_f = 0.33 (**10**), R_f = 0.21 (**11**), R_f = 0.13 (**12**), R_f = 0.32 (**13**)] to give orange solids of acetamides of **8** (394 mg, 76%), **9** (538 mg, 78%), **10** (498 mg, 60%), **11** (357 mg, 69%), **12** (496 mg, 72%) and **13** (540 mg, 65%).

Ac–L–Phe–NH–Fn–COOMe (8): **IR** (CH_2Cl_2) ν_{max}/cm^{-1}: 3418 m (NH_{free}), 3290 m, 3248 w ($NH_{assoc.}$), 1709 s ($C{=}O_{COOMe}$), 1696 s, 1668 s ($C{=}O_{CONH}$), 1574, 1558, 1540, 1535, 1516, 1507, 1498, 1466 (amide II). **^{1}H-NMR** (600 MHz, $CDCl_3$) *δ*: 7.68 (s, 1H, NH_{Fn}), 7.33-7.24 (m, 5H, CH_{Phe}), 6.43 (d, *J* = 7.6 Hz, 1H, NH_{Ac}), 4.77 (q, *J* = 7.3 Hz, 1H, $CH_{\alpha\text{-Phe}}$), 4.69 (s, 1H, CH_{Fn}), 4.65 (s, 1H, CH_{Fn}), 4.57 (s, 1H, CH_{Fn}), 4.45 (s, 1H, CH_{Fn}), 4.29 (s, 2H, CH_{Fn}), 4.03 (s, 1H, CH_{Fn}), 4.00 (s, 1H, CH_{Fn}), 3.78 (s, 3H, $CH_{3\text{-COOMe}}$), 3.1 (dd, *J*= 7.4 Hz, 13.8 Hz, 1H, $CH_{\beta1\text{-Phe}}$), 3.09 (dd, *J*= 6.8 Hz, 13.9 Hz, 1H, $CH_{\beta2\text{-Phe}}$), 2.02 [s, 3H, $CH_{3\text{-Ac}}$] ppm. **^{13}C-NMR** (150 MHz, $CDCl_3$) *δ*: 171.92 (1C, CO^b_{Fn}), 170.62 (1C, CO^a_{Fn}), 169.71 (1C, CO_{Ac}), 136.78 (1C, $C_{q\text{-}\gamma Phe}$), 129.49, 129.46, 128.85, 127.21 (5C, CH_{Phe}), 94.40 (1C, C_q-1_{Fn}), 72.72 (1C, CH_{Fn}), 72.05 (1C, C_q-6_{Fn}), 71.40, 71.28, 66.84, 66.58, 63.89, 63.44 (7C, CH_{Fn}), 55.01 (1C, $CH_{\alpha\text{-Phe}}$), 51.82 (1C, $CH_{3\text{-COOMe}}$), 38.23 (1C, $CH_{2\beta\text{-Phe}}$), 23.35 ($CH_{3\text{-Ac}}$) ppm.

Ac–L–Val–NH–Fn–COOMe (9): **IR** (CH_2Cl_2) ν_{max}/cm^{-1}: 3421 m (NH_{free}), 3287 m, 3245 m ($NH_{assoc.}$), 1711 s ($C{=}O_{COOMe}$), 1684 s, 1670 s, 1661 s ($C{=}O_{CONH}$), 1569, 1559, 1540, 1517, 1508, 1490, 1466 (amide II). **^{1}H-NMR** (600 MHz, $CDCl_3$) *δ*: 8.21 (s, 1H, NH_{Fn}), 6.49 (d, *J* = 8.7 Hz, 1H, NH_{Ac}), 4.74 (s, 1H, CH_{Fn}), 4.73 (s, 1H, CH_{Fn}), 4.61 (s, 1H, CH_{Fn}), 4.60 (s, 1H, CH_{Fn}), 4.41 (t, *J* = 7.7 Hz, 1H, $CH_{\square\text{-Val}}$), 4.37 (s, 2H, CH_{Fn}), 4.03 (s, 2H, CH_{Fn}), 3.78 (s, 3H, $CH_{3\text{-COOMe}}$), 2.18-2.13 (m, 1H, $CH_{\square\square Val}$), 2.10 (s, 3H, $CH_{3\text{-Ac}}$), 1.04 [d, *J* = 6.7 Hz, 3H, $(CH_3)_{\gamma\text{-aVal}}$], 1.01 [d, *J* = 6.7 Hz, 3H, $(CH_3)_{\gamma\text{-bVal}}$] ppm. **^{13}C-NMR** (150 MHz, $CDCl_3$) *δ*: 172.04 (1C, CO^b_{Fn}), 170.70 (1C, CO^a_{Fn}), 170.25 (1C, CO_{Ac}), 95.00 (1C, C_q-1_{Fn}), 72.73, 72.68 (2C, CH_{Fn}), 72.01 (1C, C_q-6_{Fn}), 71.39, 71.22, 66.72, 66.60, 63.42, 63.38 (6C, CH_{Fn}), 59.13 (1C, $CH_{\alpha\text{-Val}}$), 51.80 (1C, $CH_{3\text{-COOMe}}$), 31.16 (1C, $CH_{\beta\text{-Val}}$), 23.51 (1C, $CH_{3\text{-Ac}}$), 19.50 [$(CH_3)_{\gamma\text{-aVal}}$], 18.49 [$(CH_3)_{\gamma\text{-bVal}}$] ppm.

Ac–L–Leu–NH–Fn–COOMe (10): **IR** (CH_2Cl_2) ν_{max}/cm^{-1}: 3425 m (NH_{free}), 3289 m, 3241 w ($NH_{assoc.}$), 1709 s ($C{=}O_{COOMe}$), 1693 s, 1673 s, 1669 s ($C{=}O_{CONH}$), 1562, 1557, 1538, 1520, 1515, 1467 (amide II). **^{1}H-NMR** (600 MHz, $CDCl_3$) *δ*: 8.07 (s, 1H, NH_{Fn}), 6.33 (d, *J* = 8.3 Hz, 1H, NH_{Ac}), 4.74 (s, 1H, CH_{Fn}), 4.71 (s, 1H, CH_{Fn}), 4.62 (s, 1H, CH_{Fn}), 4.59 (s, 1H, CH_{Fn}), 4.53 (q, *J* = 8.3 Hz, 1H, $CH_{\alpha\text{-Leu}}$), 4.36 (pt, 2H, CH_{Fn}), 4.03 (s, 1H, CH_{Fn}), 4.00 (s, 1H, CH_{Fn}), 3.80 (s, 3H, $CH_{3\text{-COOMe}}$), 2.07 (s, 3H, $CH_{3\text{-Ac}}$), 1.81-1.76 (m, 1H, $CH_{2\text{-}\beta1\text{-Leu}}$), 1.73-1.68 (m, 1H, $CH_{\gamma\text{-Leu}}$), 1.60-1.55 (m, 1H, $CH_{2\text{-}\beta2\text{-Leu}}$), 0.98 [d, *J* = 6.6 Hz, 3H, $(CH_3)_{\delta\text{-a-Leu}}$], 0.96 [d, *J* = 6.6 Hz, 3H, $(CH_3)_{\delta\text{-b-Leu}}$] ppm. **^{13}C-NMR** (150 MHz, $CDCl_3$) *δ*: 171.95 (1C, CO^b_{Fn}), 170.90 (1C, CO^a_{Fn}), 170.78 (1C, CO_{Ac}), 95.17 (1C, C_q-1_{Fn}), 72.70, 72.66 (2C, CH_{Fn}), 72.08 (1C, C_q-6_{Fn}), 71.43, 71.22, 66.72, 66.39, 63.48, 63.21 (6C, CH_{Fn}), 52.29 (1C, $CH_{\alpha\text{-Leu}}$), 51.83 (1C, $CH_{3\text{-COOMe}}$), 40.83 [1C, $(CH_2)_{\beta\text{-Leu}}$], 24.99 (1C, $CH_{\gamma\text{-Leu}}$), 23.38 (1C, $CH_{3\text{-Ac}}$), 23.04, 22.41 [2C, $(CH_3)_{2\text{-}\delta\text{-Leu}}$] ppm.

Ac–D–Phe–NH–Fn–COOMe (11): **IR** (CH_2Cl_2) ν_{max}/cm^{-1}: 3419 m (NH_{free}), 3310 w, 3292 m, 3243 w ($NH_{assoc.}$), 1707 s ($C{=}O_{COOMe}$), 1690 s, 1666 s, 1654 s ($C{=}O_{CONH}$), 1605, 1575, 1558, 1539, 1506, 1497 (amide II). **^{1}H-NMR** (600 MHz, $CDCl_3$) *δ*: 7.77 (d, 1H, NH_{Fn}), 7.32-7.24 (m, 5H, CH_{Phe}), 6.46 (pt, 1H, NH_{Ac}), 4.79-4.75 (m, 1H, $CH_{\alpha\text{-Phe}}$), 4.68 (m, 1H, CH_{Fn}), 4.64 (m, 1H, CH_{Fn}), 4.58-4.55 (m, 1H, CH_{Fn}), 4.46 (m, 1H, CH_{Fn}), 4.29 (m, 2H, CH_{Fn}), 4.03 (s, 1H, CH_{Fn}), 4.00 (s, 1H, CH_{Fn}), 3.76 (s, 3H, $CH_{3\text{-COOMe}}$), 3.17-3.13 (m, 1H, $CH_{\beta1\text{-Phe}}$), 3.10-3.05 (m, 1H, $CH_{\beta2\text{-Phe}}$), 2.02 [s, 3H, CH_{3Ac}] ppm. **^{13}C-NMR** (150 MHz,

$CDCl_3$) δ: 171.99 (1C, CO^b_{Fn}), 170.60 (1C, CO^a_{Fn}), 169.70 (1C, CO_{Ac}), 136.74 (1C, $C_{q\text{-}\gamma Phe}$), 129.47, 129.45, 128.83, 127.19 (5C, CH_{fenil}), 94.38 (1C, $C_q\text{-}1_{Fn}$), 72.72 (1C, CH_{Fn}), 71.98 (1C, $C_q\text{-}6_{Fn}$), 71.40, 71.26, 66.62, 66.57, 66.42, 63.89, 63.44 (7C, CH_{Fn}), 54.96 (1C, $CH_{\alpha\text{-}Phe}$), 51.85 ($CH_{3\text{-}COOMe}$), 38.22 (1C, $CH_{2\beta\text{-}Phe}$), 23.35 (1C, $CH_{3\text{-}Ac}$) ppm.

Ac–D–Val–NH–Fn–COOMe (12): IR (CH_2Cl_2) ν_{max}/cm^{-1}: 3420 m (NH_{free}), 3286 m, 3235 m ($NH_{assoc.}$), 1707 s ($C{=}O_{COOMe}$), 1686 s, 1672 s, 1660 s ($C{=}O_{CONH}$), 1605, 1569, 1539, 1514, 1508, 1489 (amide II). **^{1}H-NMR** (600 MHz, $CDCl_3$) δ: 8.19 (s, 1H, NH_{Fn}), 6.45 (d, J = 8.91 Hz, 1H, NH_{Ac}), 4.74 (s, 1H, CH_{Fn}), 4.72 (s, 1H, CH_{Fn}), 4.61 (s, 1H, CH_{Fn}), 4.59 (s, 1H, CH_{Fn}), 4.40 (t, J = 8.3 Hz, 1H, $CH_{\alpha\text{-}Val}$), 4.37 (pt, 2H, CH_{Fn}), 4.03 (pt, 2H, CH_{Fn}), 3.78 (s, 3H, $CH_{3\text{-}COOMe}$), 2.19-2.13 (m, 1H, $CH_{\beta\text{-}Val}$), 2.10 (s, 3H, $CH_{3\text{-}Ac}$), 1.04 [d, J = 6.8 Hz, 3H, $(CH_3)_{\gamma\text{-}aVal}$], 1.01 [d, J = 6.6 Hz, 3H, $(CH_3)_{\gamma\text{-}bVal}$] ppm. **^{13}C-NMR** (150 MHz, $CDCl_3$) δ: 172.06 (1C, CO^b_{Fn}), 170.66 (1C, CO^a_{Fn}), 170.19 (1C, CO_{Ac}), 94.96 (1C, $C_q\text{-}1_{Fn}$), 72.72, 72.68 (2C, CH_{Fn}), 71.95 (1C, $C_q\text{-}6_{Fn}$), 71.37, 71.20, 66.71, 66.59, 63.39, 63.36 (6C, CH_{Fn}), 59.07 (1C, $CH_{\alpha\text{-}Val}$), 51.82 (1C, $CH_{3\text{-}COOMe}$), 31.16 (1C, $CH_{\beta\text{-}Val}$), 23.53 (1C, $CH_{3\text{-}Ac}$), 19.50 [1C, $(CH_3)_{\gamma\text{-}aVal}$], 18.47 [1C, $(CH_3)_{\gamma\text{-}bVal}$] ppm.

Ac–D–Leu–NH–Fn–COOMe (13): IR (CH_2Cl_2) ν_{max}/cm^{-1}: 3421 m (NH_{free}), 3290 m, 3241 w ($NH_{assoc.}$), 1708 s ($C{=}O_{COOMe}$), 1699 s, 1652 s, 1636 s ($C{=}O_{CONH}$), 1574, 1556, 1541, 1516, 1508, 1488 (amide II). **^{1}H-NMR** (600 MHz, $CDCl_3$) δ: 8.23 (s, 1H, NH_{Fn}), 6.47 (d, J = 8.22 Hz, 1H, NH_{Ac}), 4.73 (pt, 1H, CH_{Fn}), 4.71 (pt, 1H, CH_{Fn}), 4.61 (pt, 2H, CH_{Fn}), 4.54 (q, J = 8.3 Hz, 1H, $CH_{\alpha\text{-}Leu}$), 4.02 (pt, 2H, CH_{Fn}), 4.00 (pt, 2H, CH_{Fn}), 3.79 (s, 3H, $CH_{3\text{-}COOMe}$), 2.05 (s, 3H, $CH_{3\text{-}Ac}$), 1.78-1.74 (m, 1H, $CH_{2\text{-}\beta 1\text{-}Leu}$), 1.71-1.69 (m, 1H, $CH_{\gamma\text{-}Leu}$), 1.60-1.55 (m, 1H, $CH_{2\text{-}\beta 2\text{-}Leu}$), 0.98 [d, J = 6.6 Hz, 3H, $(CH_3)_{\delta\text{-}a\text{-}Leu}$], 0.96 [d, J = 6.6 Hz, 3H, $(CH_3)_{\delta\text{-}b\text{-}Leu}$] ppm. **^{13}C-NMR** (150 MHz, $CDCl_3$) δ: 171.97 (1C, CO^b_{Fn}), 170.90 (1C, CO^a_{Fn}), 169.92 (1C, CO_{Ac}), 95.22 (1C, $C_q\text{-}1_{Fn}$), 72.71, 72.67 (2C, CH_{Fn}), 71.99 (1C, $C_q\text{-}6_{Fn}$), 71.38, 71.19, 66.67, 66.40, 63.34, 63.16 (6C, CH_{Fn}), 52.28 (1C, $CH_{\alpha\text{-}Leu}$), 51.82 (1C, $CH_{3\text{-}COOMe}$), 40.86 [1C, $(CH_2)_{\beta\text{-}Leu}$], 24.97 (1C, $CH_{\gamma\text{-}Leu}$), 23.34 (1C, $CH_{3\text{-}Ac}$), 23.03, 22.39 [2C, $(CH_3)_2\text{-}_{\delta\text{-}Leu}$] ppm.

Synthesis of Ac–L–AA–NH–Fn–COOH (14–19): Esters **8–13** (1 mmol) were dissolved in MeOH (3 mL) and hydrolyzed by heating at reflux in the presence of NaOH (1 mmol) and H_2O (0.1 mL) for 1 h. The reaction mixtures were concentrated *in vacuo* to leave the salts, which were dissolved in 5% $NaHCO_3$ and washed with CH_2Cl_2 to remove the residual esters. The water phases were acidified with 10% HCl and extracted with EtOAc. The organic phases were washed with brine, dried with Na_2SO_4, and concentrated in vacuo to get the crude products of the desired acids **14** (348 mg, 81%), **15** (322 mg, 84%), **16** (342 mg, 86%), **17** (344 mg, 80%), **18** (307 mg, 80%) and **19** (314 mg, 79%) as orange resins.

Ac–L–Phe–NH–Fn–COOH (14): IR (CH_2Cl) ν_{max}/cm^{-1}: 3411 m (NH_{free}), 3285 m, 3256 m ($NH_{assoc.}$), 3143-3073 br (OH, COOH), 1716 s, 1696 s, 1683 s, 1654 s ($C{=}O_{COOH,\ CONH}$), 1575, 1569, 1558, 1540, 1533, 1522, 1508, 1498, 1485, 1474 (amide II).

Ac–L–Val–NH–Fn–COOH (15): IR (CH_2Cl) ν_{max}/cm^{-1}: 3417 m (NH_{free}), 3280 m, 3237 m ($NH_{assoc.}$), 3152-3056 br (OH, COOH), 1712 s, 1684 s, 1656 s ($C{=}O_{COOH,CONH}$), 1574, 1540, 1521, 1478 (amide II).

Ac–L–Leu–NH–Fn–COOH (16): IR (CH_2Cl) ν_{max}/cm^{-1}: 3419 m (NH_{free}), 3287 m, 3277 m ($NH_{assoc.}$), 3161-3070 br (OH, COOH), 1714 s, 1696 s, 1686 s, 1653 s ($C{=}O_{COOH,\ CONH}$), 1569, 1558, 1539, 1533, 1528, 1522, 1509, 1472 (amide II).

Ac–D–Phe–NH–Fn–COOH (17): IR (CH_2Cl) ν_{max}/cm^{-1}: 3419 m (NH_{free}), 3292 m, 3243 m ($NH_{assoc.}$), 3150-3070 br (OH, COOH), 1716 s, 1690 s, 1666 s, 1654 s ($C{=}O_{COOH,\ CONH}$), 1605, 1575, 1558, 1539, 1506, 1497 (amide II).

Ac–D–Val–NH–Fn–COOH (18): IR (CH_2Cl) ν_{max}/cm^{-1}: 3420 m (NH_{free}), 3286 m, 3235 m ($NH_{assoc.}$), 3150-3055 br (OH, COOH), 1715 s, 1686 s, 1672 s, 1660 s ($C{=}O_{COOH,\ CONH}$), 1605, 1569, 1539, 1514, 1508, 1489 (amide II).

Ac–D–Leu–NH–Fn–COOH (19): IR (CH_2Cl) ν_{max}/cm^{-1}: 3421 m (NH_{free}), 3290 m, 3241 m ($NH_{assoc.}$), 3161-3071 br (OH, COOH), 1715 s, 1699 s, 1652 s, 1636 s ($C{=}O_{COOH,\ CONH}$), 1574, 1556, 1541, 1516, 1508, 1488 (amide II).

Synthesis of Ac–L–AA–NH–Fn–NH–Boc (26–31): The carboxylic acid groups of **14–19** were converted to carbamates **26–31** *via* unstable azides **20–25**. The acid precursors **14–19** (1 mmol) were suspended in water (0.5 mL), and sufficient acetone was added to complete the solutions. Triethylamine (1.14 mmol) in acetone (3 mL) was added at 0 °C, and the intermediate amines were transferred to mixed anhydrides by action of ethyl chloroformate (0.75 mmol) in acetone (0.8 mL). After stirring for 30 min at 0 °C, a solution of sodium azide (1.5 mmol) in water (1 mL) was added. The mixtures were stirred for 1 h at 0 °C and concentrated *in vacuo* to afford an unstable azides **20–25** which were then converted *in situ* to the corresponding carbamates by heating with *t*-BuOH (5 mL) at 65 °C for 5 h. The reaction mixtures were concentrated *in vacuo* and purified by preparative chromatography [EtOAc; R_f = 0.78 **(26)**, R_f = 0.59 **(27)**, R_f = 0.80 **(28)**, R_f = 0.75 **(29)**, R_f = 0.52 **(30)**, R_f = 0.82 **(31)**] to give orange solids of acetamides of **26** (147 mg, 29 %), **27** (138 mg, 30%), **28** (166 mg, 35%), **29** (126 mg, 25 %), **30** (133 mg, 29%) and **31** (142 mg, 30%).

Ac–L–Phe–NH–Fn–NH–Boc (26): IR (CH_2Cl_2) ν_{max}/cm^{-1}: 3427 m (NH_{free}), 3318 w ($NH_{assoc.}$), 1704 s, 1683 s, 1673 s ($C{=}O_{CONH}$), 1531, 1507, 1498, 1456 (amide II). **^{1}H-NMR** (600 MHz, $CDCl_3$) δ: 7.77 (s, 1H, NH^a_{Fn}), 7.32-7.20 (m, 5H, CH_{Phe}), 6.30 (d, J = 7.6 Hz, 1H, NH_{Ac}), 5.90 (d, J = 7.6 Hz, 1H, NH^b_{Fn}), 4.70 (q, J = 7.2 Hz, 1H, CH_{Phe}), 4.51 (pt, 2H, CH_{Fn}), 4.32 (pt, 2H, CH_{Fn}), 4.16-4.08 (m, 4H, CH_{Fn}), 3.16-3.08 (m, 2H, $CH_{\beta\text{-}1\text{-}Phe}$, $CH_{\beta\text{-}2\text{-}Phe}$), 1.99 [s, 3H, $CH_{3\text{-}Ac}$], 1.49 [s, 9H, $(CH_3)_{3\text{-}Boc}$] ppm. **^{13}C-NMR** (150 MHz, $CDCl_3$) δ: 170.98 (1C, CO^a_{Fn}), 169.46 (1C, CO_{Ac}), 153.87 (1C, CO^b_{Fn}), 136.75 (1C, $C_{q\text{-}\gamma Phe}$), 129.58, 128.82, 127.20 (5C, CH_{Phe}), 80.62 (1C, C_{qBoc}), 62.63, 62.54, 62.53, 62.51 (8C, CH_{Fn}), 55.06 (1C, $CH_{\alpha\text{-}Phe}$), 38.37 (1C, $CH_{2\beta\text{-}Phe}$), 28.58 [3C, $(CH_3)_{3Boc}$], 23.37 (1C, $CH_{3\text{-}Ac}$) ppm.

Ac–L–Val–NH–Fn–NH–Boc (27): IR (CH_2Cl_2) ν_{max}/cm^{-1}: 3427 m (NH_{free}), 3322 w, 3311 w, 3290 w ($NH_{assoc.}$), 1704 s, 1684 s, 1671 s ($C{=}O_{CONH}$), 1630, 1507, 1457 (amide II). **^{1}H-NMR** (600 MHz, $CDCl_3$) δ: 8.00 (s, 1H, NH^a_{Fn}), 6.25 (s, 1H, NH_{Ac}), 6.02 (s, 1H, NH^b_{Fn}), 4.66 (pt, 2H, CH_{Fn}), 4.55 (pt, 2H, CH_{Fn}), 4.39-4.30 (m, 1H, $CH_{\alpha\text{-}Val}$), 4.12 (s, 2H, CH_{Fn}), 4.05 (s, 2H, CH_{Fn}), 2.19-2.14 (m, 1H, $CH_{\beta\text{-}Val}$), 2.05 (s, 3H, $CH_{3\text{-}Ac}$), 1.50 [s, 9H, $(CH_3)_{3\text{-}Boc}$], 1.01 [d, J = 6.3 Hz, 3H, $(CH_3)_{\gamma\text{-}aVal}$], 0.98 [d, J = 6.2 Hz,3H, $(CH_3)_{\gamma\text{-}bVal}$] ppm. **^{13}C-NMR** (150 MHz, $CDCl_3$) δ: 169.79 (1C, CO^a_{Fn}), 169.15 (1C, CO_{Ac}), 153.12 (1C, CO^b_{Fn}), 79.95 (1C, C_{qBoc}), 74.45 (1C, C_q-1_{Fn}), 72.93, 71.87, 70.81, 70.69, 67.45, 66.27, 63.54, 62.14 (8C, CH_{Fn}), 58.45 (1C, $CH_{\alpha\text{-}Val}$), 30.47 (1C, $CH_{\beta\text{-}Val}$), 27.87 [3C, $(CH_3)_{3Boc}$], 22.81 (1C, $CH_{3\text{-}Ac}$), 18.81 [1C, $(CH_3)_{\gamma\text{-}aVal}$], 17.71 [1C, $(CH_3)_{\gamma\text{-}bVal}$] ppm.

Ac–L–Leu–NH–Fn–NH–Boc (28): IR (CH_2Cl_2) ν_{max}/cm^{-1}: 3428 m ($NH_{slobodni}$), 3306 w ($NH_{assoc.}$), 1704 s, 1682 s, 1673 s ($C{=}O_{CONH}$), 1531, 1507, 1480, 1458, 1438, 1420 (amide II). **^{1}H-NMR** (600 MHz, $CDCl_3$) δ: 8.02 (s, 1H, NH_{Fn}), 6.20 (s, 1H, NH_{Ac}), 6.02 (s, 1H, $NH^b{}_{Fn}$), 4.69-4.54 (m, 2H, CH_{Fn}), 4.32 (s, 2H, CH_{Fn}), 4.49-4.46 (m, 1H, $CH_{\alpha\text{-Leu}}$), 4.23-4.13 (m, 4H, CH_{Fn}), 2.02 (s, 3H, $CH_{3\text{-Ac}}$), 1.75-1.51 [m, 3H, $(CH_2)_{\beta\text{-Leu}}$, $CH_{\gamma\text{-Leu}}$], 1.49 [s, 9H, $(CH_3)_{3\text{-Boc}}$], 0.98-0.96 [m, 6H, $(CH_3)_{\delta\text{-a-Leu}}$, $(CH_3)_{\delta\text{-b-Leu}}$] ppm. **^{13}C-NMR** (150 MHz, $CDCl_3$) δ: 170.62 (1C, $CO^a{}_{Fn}$), 170.52 (1C, CO_{Ac}), 150.30 (1C, $CO^b{}_{Fn}$), 80.56 (1C, C_{qBoc}), 70.76 (1C, C_q-1_{Fn}), 67.73, 63.05, 63.02, 62.82, 62.80 (8C, CH_{Fn}), 52.36 (1C, $CH_{\alpha\text{-Leu}}$), 41.29 [1C, $(CH_2)_{\beta\text{-Leu}}$], 28.57 [3C, $(CH_3)_{3Boc}$], 24.99 (1C, $CH_{\gamma\text{-Leu}}$), 23.38 (1C, $CH_{3\text{-Ac}}$), 23.09, 22.41 [2C, $(CH_3)_2$-$_{\delta\text{-Leu}}$] ppm.

Ac–D–Phe–NH–Fn–NH–Boc (29): IR (CH_2Cl_2) ν_{max}/cm^{-1}: 3427 m (NH_{free}), 3316 w ($NH_{assoc.}$), 1701 s, 1680 s, 1670 s ($C{=}O_{CONH}$), 1530, 1505, 1499, 1455 (amide II). **^{1}H-NMR** (600 MHz, $CDCl_3$) δ: 7.90 (s, 1H, $NH^a{}_{Fn}$), 7.32-7.23 (m, 5H, CH_{Phe}), 6.42 (s, 1H, NH_{Ac}), 5.93 (s 1H, $NH^b{}_{Fn}$), 4.72-4.71 (m, 1H, CH_{Phe}), 4.49 (s, 2H, CH_{Fn}), 4.30 (s, 2H, CH_{Fn}), 4.14-4.01 (m, 4H, CH_{Fn}), 3.16-3.07 (m, 2H, $CH_{\beta\text{-1-Phe}}$, $CH_{\beta\text{-2-Phe}}$), 1.98 [s, 3H, $CH_{3\text{-Ac}}$], 1.48 [s, 9H, $(CH_3)_{3\text{-Boc}}$] ppm. **^{13}C-NMR** (150 MHz, $CDCl_3$) δ: 170.37 (1C, $CO^a{}_{Fn}$), 169.61 (1C, CO_{Ac}), 153.90 (1C, $CO^b{}_{Fn}$), 136.69 (1C, $C_{q\text{-}\gamma Phe}$), 129.55, 128.78, 127.17 (5C, CH_{Phe}), 80.59 (1C, C_{qBoc}), 74.97 (1C, C_q-1_{Fn}), 69.67, 66.37, 65.71, 64.70, 64.49, 92.90, 62.60, 60.54 (8C, CH_{Fn}), 55.04 (1C, $CH_{\alpha\text{-Phe}}$), 38.42 (1C, $CH_{2\beta\text{-Phe}}$), 28.54 [3C, $(CH_3)_{3Boc}$], 23.36 (1C, $CH_{3\text{-Ac}}$) ppm.

Ac–D–Val–NH–Fn–NH–Boc (30): IR (CH_2Cl_2) ν_{max}/cm^{-1}: 3425 m (NH_{free}), 3320 w, 3309 w, 3288 w ($NH_{assoc.}$), 1703 s, 1680 s, 1671 s ($C{=}O_{CONH}$), 1629, 1506, 1456 (amide II). **^{1}H-NMR** (600 MHz, $CDCl_3$) δ: 8.04 (s, 1H, $NH^a{}_{Fn}$), 6.31 (s, 1H, NH_{Ac}), 6.00 (s,1H, $NH^b{}_{Fn}$), 4.74 (pt, 2H, CH_{Fn}), 4.60 (pt, 2H, CH_{Fn}), 4.29-4.20 (s, 4H, CH_{Fn}), 4.11 (q, J =7.2 Hz, 14.4 Hz, 1H, $CH_{\alpha\text{-Val}}$), 2.17-2.12 (m, 1H, $CH_{\beta\text{-Val}}$), 2.04 (s, 3H, $CH_{3\text{-Ac}}$), 1.48 [s, 9H, $(CH_3)_{3\text{-Boc}}$], 1.01 [d, J = 6.1 Hz, 3H, $(CH_3)_{\gamma\text{-aVal}}$], 0.96 [d, J = 6.1 Hz, 3H, $(CH_3)_{\gamma\text{-bVal}}$] ppm. **^{13}C-NMR** (150 MHz, $CDCl_3$) δ: 170.52(1C, $CO^a{}_{Fn}$), 169.98 (1C, CO_{Ac}), 153.80 (1C, $CO^b{}_{Fn}$), 80.58 (1C, C_{qBoc}), 74.45 (1C, C_q-1_{Fn}), 67.07, 66.59, 66.06, 64.59, 62.97, 62.90, 62.82, 62.76 (8C, CH_{Fn}), 60.54 (1C, $CH_{\alpha\text{-Val}}$), 31.26 (1C, $CH_{\beta\text{-Val}}$), 28.53 [3C, $(CH_3)_{3Boc}$], 23.48 (1C, $CH_{3\text{-Ac}}$), 19.47 [1C, $(CH_3)_{\gamma\text{-aVal}}$], 18.41 [1C, $(CH_3)_{\gamma\text{-bVal}}$] ppm.

Ac–D–Leu–NH–Fn–NH–Boc (31): IR (CH_2Cl_2) ν_{max}/cm^{-1}: 3426 m ($NH_{slobodni}$), 3305 w ($NH_{assoc.}$), 1701 s, 1688 s, 1670 s ($C{=}O_{CONH}$), 1530, 1505, 1436, 1420 (amide II). **^{1}H-NMR** (600 MHz, $CDCl_3$) δ: 8.16 (s, 1H, NH_{Fn}), 6.35 (s, 1H, NH_{Ac}), 6.07 (s, 1H, $NH^b{}_{Fn}$), 4.68-4.50 (m, 2H, CH_{Fn}), 4.30 (s, 2H, CH_{Fn}), 4.48-4.46 (m, 1H, $CH_{\alpha\text{-Leu}}$), 4.20-4.13 (m, 4H, CH_{Fn}), 2.01 (s, 3H, $CH_{3\text{-Ac}}$), 1.73-1.66 [m, 3H, $(CH_2)_{\beta\text{-Leu}}$, $CH_{\gamma\text{-Leu}}$], 1.48 [s, 9H, $(CH_3)_{3\text{-Boc}}$], 0.96-0.94 [m, 6H, $(CH_3)_{\delta\text{-a-Leu}}$, $(CH_3)_{\delta\text{-b-Leu}}$] ppm. **^{13}C-NMR** (150 MHz, $CDCl_3$) δ: 170.80 (1C, $CO^a{}_{Fn}$), 170.61 (1C, CO_{Ac}), 154.16 (1C, $CO^b{}_{Fn}$), 80.50 (1C, C_{qBoc}), 71.79 (1C, C_q-1_{Fn}), 68.17, 66.65, 66.16, 65.90, 64.80, 64.18, 63.13, 62.82 (8C, CH_{Fn}), 52.33 (1C, $CH_{\alpha\text{-Leu}}$), 41.38 [1C, $(CH_2)_{\beta\text{-Leu}}$], 28.48 [3C, $(CH_3)_{3Boc}$], 24.94 (1C, $CH_{\gamma\text{-Leu}}$), 23.36 (1C, $CH_{3\text{-Ac}}$), 23.06, 22.38 [2C, $(CH_3)_2$-$_{\delta\text{-Leu}}$] ppm.

DFT Data

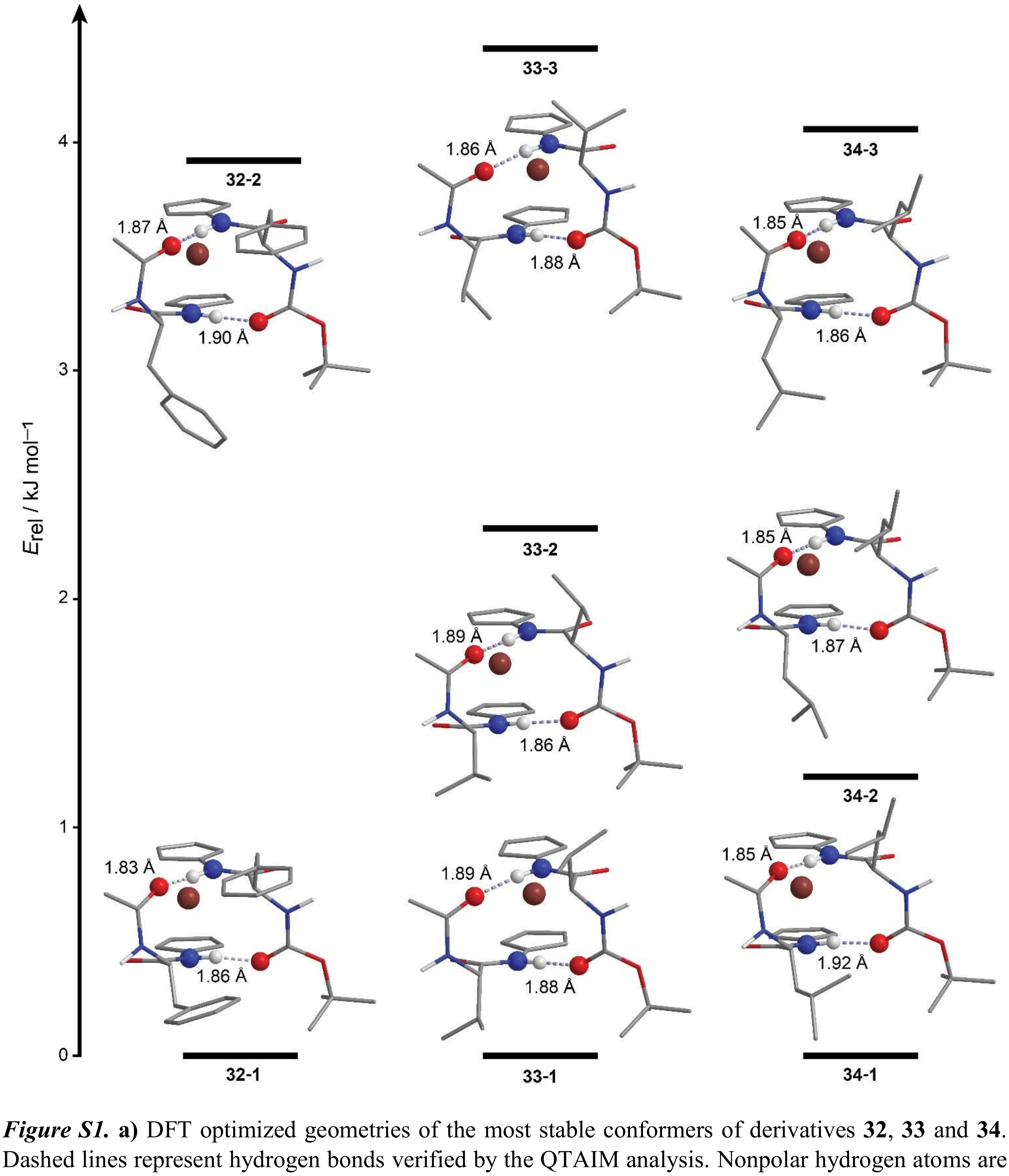

Figure S1. **a)** DFT optimized geometries of the most stable conformers of derivatives **32**, **33** and **34**. Dashed lines represent hydrogen bonds verified by the QTAIM analysis. Nonpolar hydrogen atoms are omitted for clarity.

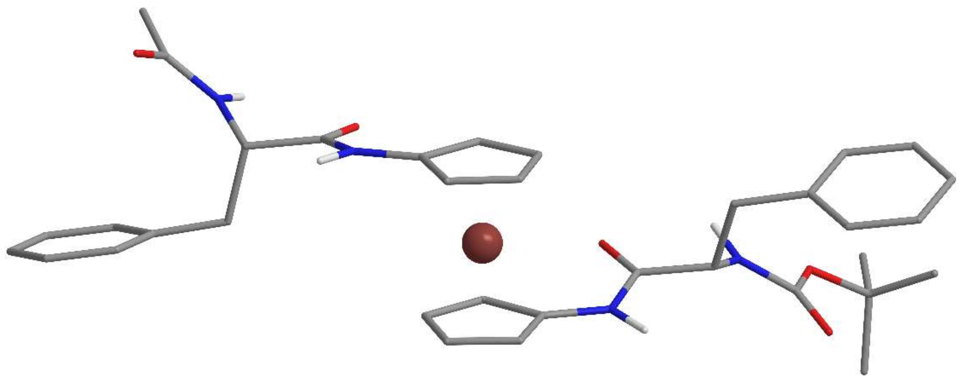

32-*open* (ΔE = 52.36 kJ mol^{-1})

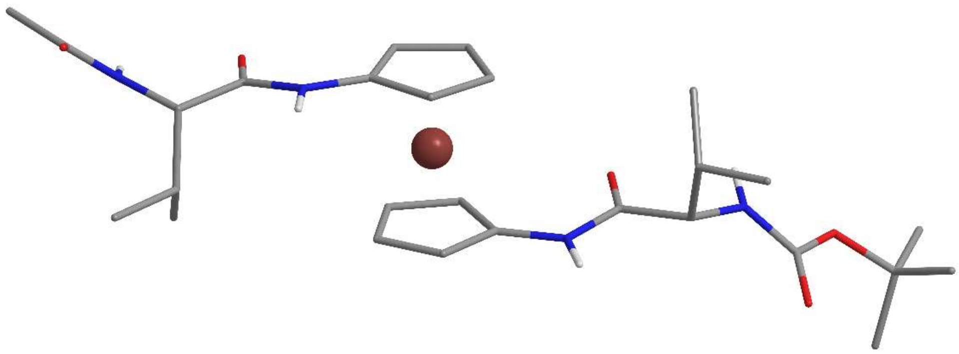

33-*open* (ΔE = 46.33 kJ mol^{-1})

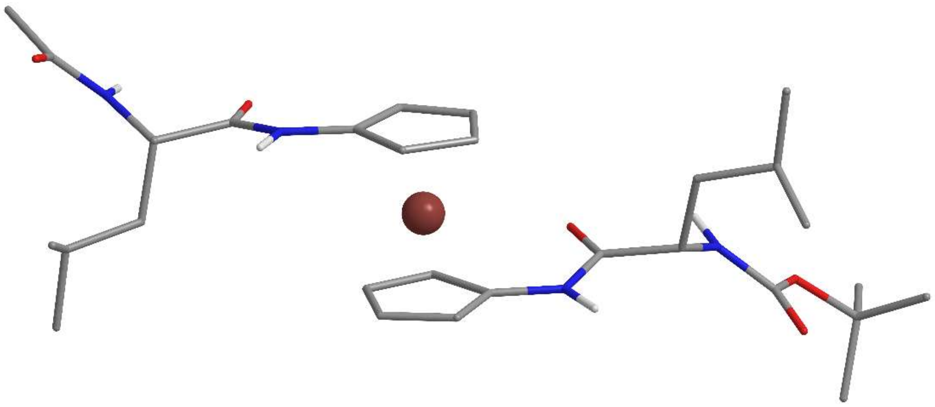

34-*open* (ΔE = 52.53 kJ mol^{-1})

Figure S1. **b)** DFT optimized *open* conformers geometries (no hydrogen bonds) of derivatives **32**, **33** and **34**. Nonpolar hydrogen atoms are omitted for clarity.

Table S1. Cartesian coordinates (in Angstroms) of the most stable conformers of derivatives **32**, **33** and **34**.

**32-1**			
atom	*x*	*y*	*z*
Fe	-4.197245	-0.218724	0.086764
C	-3.452806	1.309279	-1.090064
C	-4.876498	1.263422	-1.185850
C	-5.242898	-0.019191	-1.688963
C	-4.051407	-0.775894	-1.907171
C	-2.944161	0.054502	-1.547391
H	-5.551887	2.055029	-0.899658
H	-6.248804	-0.376079	-1.850041
H	-3.982311	-1.791675	-2.253221
H	-2.856238	2.125930	-0.712619
C	-3.651303	-1.825894	1.270777
C	-5.059907	-1.608386	1.352901
C	-5.273370	-0.290798	1.853451
C	-4.001220	0.316295	2.082974
C	-2.998522	-0.642656	1.734682
H	-5.823256	-2.312594	1.059520
H	-6.230128	0.185376	2.005475
H	-3.813557	1.316390	2.430316
H	-3.153547	-2.710184	0.902689
N	-1.576141	-0.258374	-1.545303
H	-0.959683	0.448814	-1.145162
N	0.909716	-2.841927	-1.214359
C	0.498111	-1.507111	-1.608374
C	-1.025157	-1.461112	-1.845037
O	-1.641596	-2.430489	-2.272649
C	1.277423	-1.052373	-2.858275
H	1.297551	-3.451677	-1.919457
H	0.741098	-0.826867	-0.794196
C	0.634746	-3.352320	0.001584
O	0.067820	-2.688059	0.880188
C	1.066691	-4.774437	0.257727
H	1.652595	-4.801418	1.178992
H	0.172951	-5.384172	0.414352
H	1.653101	-5.200556	-0.557857
C	-0.919387	0.629895	2.068928
N	-1.602065	-0.499407	1.755441
O	-1.429361	1.673233	2.464016
H	-1.065070	-1.282736	1.378882
C	0.607230	0.499844	1.891147
H	0.807478	-0.230711	1.109555
C	0.895462	2.297485	0.269191
N	1.181625	1.769275	1.484312
O	0.341917	1.671613	-0.631943
H	1.461066	2.418309	2.206561
O	1.329561	3.560643	0.177184
C	1.123784	4.373687	-1.049879
C	1.890840	3.753391	-2.216294
H	1.856140	4.435200	-3.070275
H	2.938351	3.598488	-1.945353

H	1.457589	2.800529	-2.515825
C	-0.370881	4.513127	-1.336095
H	-0.898719	4.852748	-0.441164
H	-0.515702	5.257979	-2.123179
H	-0.805175	3.570992	-1.666790
C	1.729582	5.716028	-0.649592
H	1.203647	6.133783	0.212330
H	1.649539	6.420437	-1.480881
H	2.785382	5.600454	-0.392416
C	1.280334	0.001327	3.185252
H	0.756638	-0.903792	3.502394
H	1.068691	-1.740526	-3.683151
H	0.893895	-0.071297	-3.148128
H	1.144433	0.749295	3.972289
C	5.505278	-0.879273	-1.976148
C	4.632544	0.072171	-1.444695
C	3.275110	0.022950	-1.749588
C	2.764028	-0.976833	-2.587463
C	3.646180	-1.923121	-3.116677
C	5.008969	-1.875527	-2.815323
H	6.562366	-0.843931	-1.736399
H	5.007744	0.847324	-0.785653
H	2.599276	0.759227	-1.330088
H	3.267092	-2.701268	-3.772142
H	5.680052	-2.617152	-3.235477
C	5.460656	-0.816167	2.455609
C	4.476185	-1.688367	1.987414
C	3.132546	-1.428599	2.244828
C	2.747944	-0.293492	2.970041
C	3.741050	0.572477	3.436121
C	5.089483	0.313763	3.182813
H	6.507286	-1.015715	2.253181
H	4.755662	-2.566781	1.415850
H	2.367016	-2.103008	1.876342
H	3.459755	1.455240	4.001910
H	5.847409	0.996380	3.552246

**33-1**			
atom	*x*	*y*	*z*
Fe	3.024222	-1.238065	-0.334388
C	1.531032	-2.001676	-1.542151
C	2.777654	-2.624687	-1.851717
C	3.688457	-1.607249	-2.261131
C	3.013736	-0.349374	-2.208857
C	1.674274	-0.598859	-1.773466
H	2.994235	-3.677965	-1.759927
H	4.722758	-1.751213	-2.534599
H	3.430345	0.618822	-2.421680
H	0.642935	-2.486010	-1.165624
C	3.392384	0.208880	1.097231
C	4.540654	-0.623640	0.933070
C	4.160134	-1.963266	1.237597
C	2.776568	-1.969603	1.591671
C	2.306983	-0.620173	1.516586
H	5.514827	-0.293820	0.606003

H	4.795233	-2.834299	1.180308
H	2.175685	-2.826191	1.839374
H	3.333187	1.271152	0.915458
N	0.641852	0.321779	-1.533996
H	-0.236467	-0.068851	-1.194951
N	-0.183782	3.659181	-0.397497
C	-0.513001	2.395637	-1.034160
C	0.779336	1.671734	-1.463284
O	1.811590	2.281039	-1.719353
C	-1.501543	2.569480	-2.213141
H	-0.083882	4.476526	-0.981557
H	-1.010558	1.777490	-0.289700
C	0.335089	3.715588	0.848541
O	0.439720	2.713848	1.568490
C	0.773148	5.073646	1.338742
H	1.856699	5.053494	1.483501
H	0.520235	5.883115	0.652005
H	0.311603	5.262856	2.310211
C	-2.854846	3.081878	-1.707088
H	-3.576592	3.127007	-2.527798
H	-3.262108	2.423560	-0.934303
H	-2.766419	4.087385	-1.284145
C	-0.109470	-0.874833	1.891322
N	1.015581	-0.124826	1.759440
O	-0.135915	-2.100699	1.867336
H	0.894660	0.885450	1.674108
C	-1.391159	-0.043060	2.100533
H	-1.298287	0.879054	1.528010
C	-2.657529	-1.005620	0.259022
N	-2.545230	-0.766128	1.591111
O	-1.975043	-0.446973	-0.595274
H	-2.982886	-1.446659	2.195708
O	-3.628991	-1.894372	0.009041
C	-3.953662	-2.326785	-1.373945
C	-4.470432	-1.134850	-2.178335
H	-4.837937	-1.486747	-3.146069
H	-5.298435	-0.651827	-1.652613
H	-3.684738	-0.400402	-2.350625
C	-2.732558	-2.985940	-2.015184
H	-2.323217	-3.752002	-1.351537
H	-3.035148	-3.467518	-2.948862
H	-1.953585	-2.258551	-2.237451
C	-5.062189	-3.349335	-1.139281
H	-5.407769	-3.747189	-2.096318
H	-4.697056	-4.179050	-0.529069
H	-5.910371	-2.886989	-0.628328
C	-0.951429	3.447845	-3.343877
H	-1.647121	3.447903	-4.187608
H	-0.831612	4.490467	-3.028871
H	0.016493	3.091321	-3.704531
C	-1.572900	0.364202	3.583775
H	-0.653386	0.897480	3.845664
C	-2.745490	1.340778	3.733798
H	-2.813978	1.696532	4.765927
H	-2.620605	2.211731	3.083437
H	-3.696097	0.862303	3.480285

C	-1.713888	-0.832504	4.533770
H	-1.737547	-0.482479	5.569571
H	-2.644756	-1.383881	4.360881
H	-0.883141	-1.534658	4.433174
H	-1.650918	1.560283	-2.608940

**34-1**			
atom	*x*	*y*	*z*
Fe	3.581406	0.471259	-0.057794
C	2.494150	2.037734	0.739895
C	3.891665	2.316591	0.824527
C	4.498419	1.291355	1.607659
C	3.483212	0.371533	2.012009
C	2.241096	0.843272	1.482918
H	4.397548	3.144875	0.352547
H	5.549935	1.201751	1.834600
H	3.614419	-0.528489	2.585663
H	1.754910	2.600096	0.189870
C	3.435299	-1.446434	-0.817825
C	4.763519	-0.950281	-0.985658
C	4.695351	0.227506	-1.786051
C	3.327156	0.469010	-2.117397
C	2.550356	-0.577762	-1.527955
H	5.654922	-1.381176	-0.556118
H	5.527687	0.853424	-2.070132
H	2.932938	1.294186	-2.682847
H	3.134285	-2.307345	-0.240337
N	0.969438	0.257249	1.571770
H	0.236356	0.699307	1.017940
N	-0.926959	-2.787080	1.930392
C	-0.808833	-1.338809	1.982631
C	0.670077	-0.932484	2.150876
O	1.463990	-1.629258	2.773686
C	-1.656035	-0.760886	3.125452
H	-1.137648	-3.274288	2.789623
H	-1.167942	-0.936806	1.037435
C	-0.461302	-3.509174	0.888716
O	-0.018079	-2.976002	-0.135855
C	-0.520077	-5.010737	1.022302
H	-1.016462	-5.422896	0.141387
H	-1.040198	-5.343548	1.922152
H	0.503535	-5.394567	1.038179
C	0.250333	0.085158	-2.100778
N	1.159340	-0.761395	-1.554940
O	0.525580	1.124031	-2.692258
H	0.798420	-1.545882	-1.009583
C	-1.209459	-0.389277	-1.947663
H	-1.285404	-0.998207	-1.047805
C	-1.955518	1.627789	-0.787355
N	-2.102565	0.752486	-1.815077
O	-1.263219	1.411181	0.201744
H	-2.502352	1.130737	-2.662970
O	-2.693160	2.729987	-0.985614
C	-2.631129	3.889859	-0.059911
C	-3.176587	3.493925	1.311138

H	-3.255340	4.387748	1.936085
H	-4.173309	3.056493	1.212985
H	-2.521668	2.779659	1.807262
C	-1.199721	4.421056	0.009543
H	-0.807439	4.588040	-0.996886
H	-1.197759	5.375505	0.542606
H	-0.541376	3.730410	0.534265
C	-3.552733	4.896089	-0.743706
H	-3.178262	5.145785	-1.739409
H	-4.561992	4.488678	-0.842133
H	-3.604176	5.812802	-0.151454
C	-1.610611	-1.235998	-3.165510
H	-0.869428	-2.033987	-3.277414
H	-1.268695	-1.144424	4.076966
H	-1.499385	0.322314	3.136078
H	-1.532778	-0.605815	-4.059575
C	-3.166607	-1.049346	3.030763
H	-3.313265	-2.135822	3.071047
C	-3.781844	-0.536536	1.72251
H	-4.861907	-0.712278	1.712311
H	-3.611942	0.537520	1.606593
H	-3.363222	-1.035279	0.84523
C	-3.879847	-0.433543	4.241575
H	-4.948454	-0.668964	4.230026
H	-3.464037	-0.804222	5.183975
H	-3.778550	0.657646	4.237463
C	-3.016338	-1.860957	-3.092629
H	-3.741146	-1.057257	-2.915408
C	-3.358072	-2.518927	-4.435759
H	-4.364329	-2.948925	-4.417974
H	-2.653772	-3.327287	-4.66313
H	-3.315613	-1.797499	-5.257928
C	-3.139352	-2.876022	-1.949266
H	-4.145655	-3.305314	-1.921657
H	-2.425078	-3.695258	-2.08279
H	-2.948003	-2.424716	-0.973047

Table S2. Relative energies (reported energies refer to standard Gibbs free energies at 298 K in kJ mol−1) of the most stable conformers of compounds **32**–**34**. Optimizations performed at the B3LYP-D3/6-311+G(d,p), LanL2DZ for Fe, level of theory, SMD model for solvent effects. Stereochemical descriptors and helicity determined from the value of pseudo-torsion angles, X–Y distances (in Å) of the selected X–H···Y hydrogen bonds connecting the 10-membered rings.

type	stereochemical descriptors	ΔE / kJ mol-1	ω / ° pseudotorsion angle	NHFc···O=C_{Boc} 10-membered	NHFc···O=C_{Ac} 10-membered
**32-1**	P-1,1'	0.00	+23.7	2.87	2.89
**32-2**	P-1,1'	3.86	+31.3	2.91	2.88
**32-3**	P-1,2'	6.89	+36.1	2.91	2.86
**32-4**	P-1,1'	7.03	+25.6	2.86	2.86
**33-1**	P-1,1'	0.00	+25.7	2.88	2.90
**33-2**	P-1,1'	2.32	+26.2	2.87	2.91
**33-3**	P-1,1'	4.39	+31.0	2.89	2.88
**34-1**	P-1,1'	0.00	+22.9	2.86	2.88
**34-2**	P-1,1'	1.22	+25.8	2.88	2.87
**34-3**	P-1,1'	4.06	+34.1	2.93	2.86

Ac–L–Phe–NH–Fn–NH–L–Phe–Boc (32)

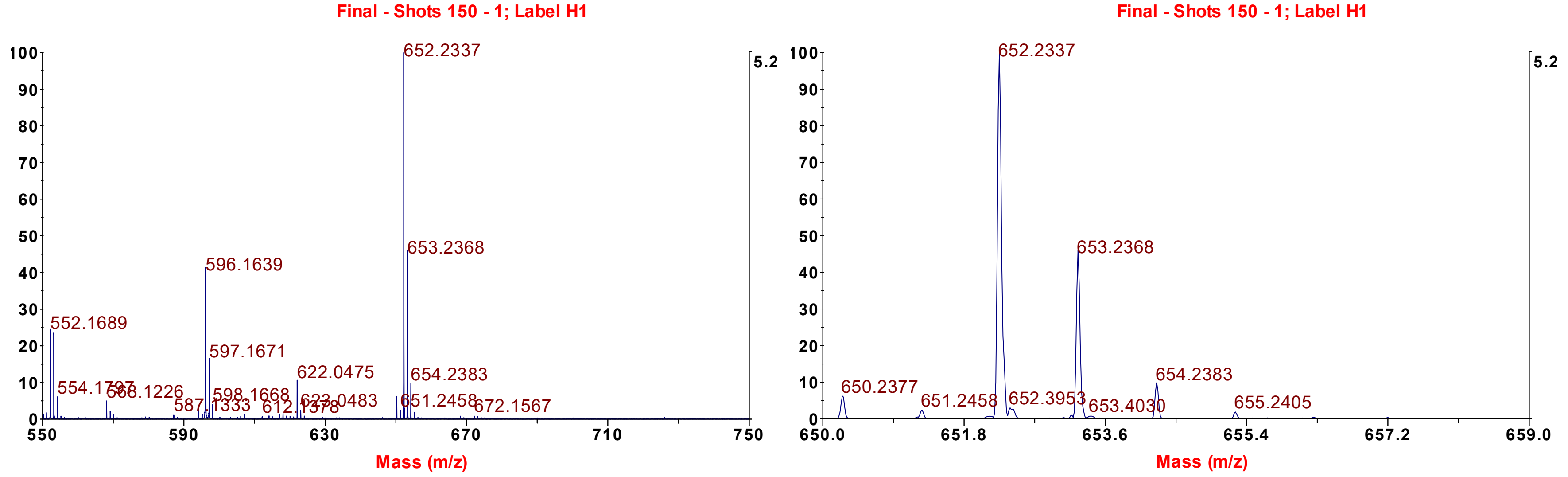

Figure S2. HRMS spectrum of compound **32.**

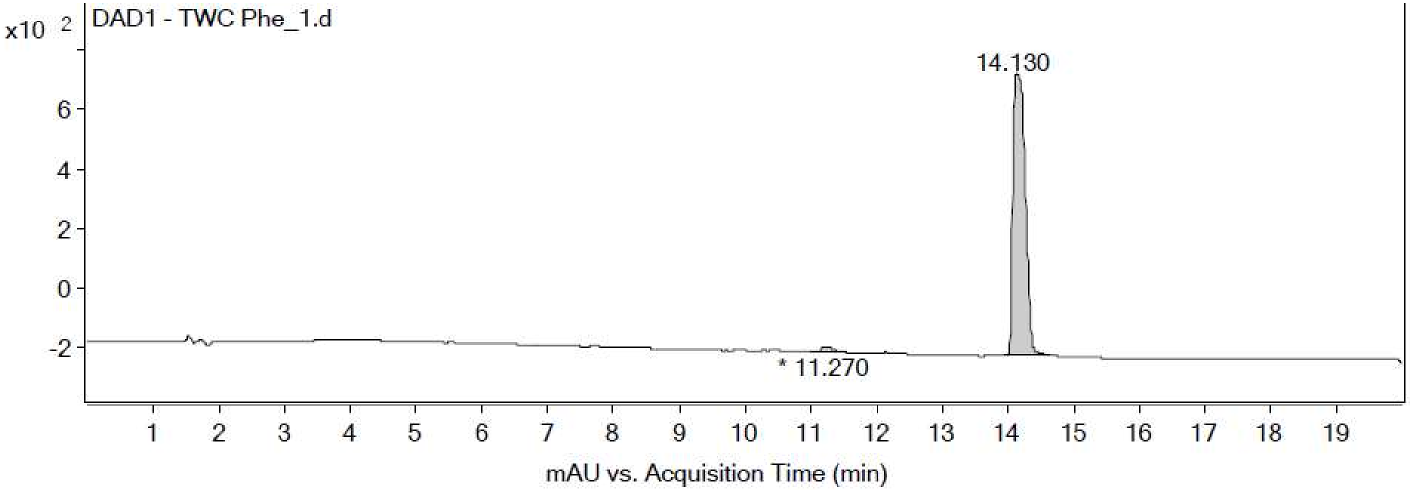

Integration Peak List

Peak	Start	RT	End	Height	Area	Area %
1	10.984	11.27	11.55	16.37	194.88	1.61
2	13.964	14.13	14.638	943.99	12117.92	100

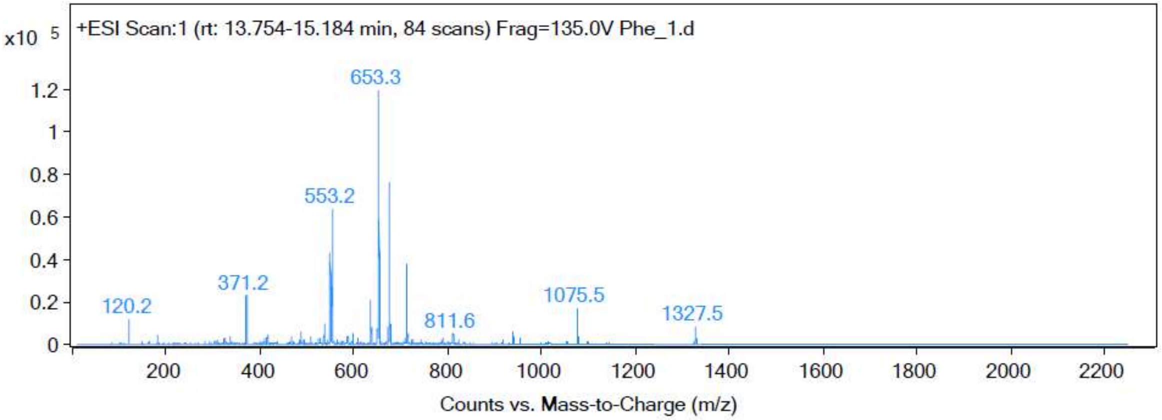

Peak List

m/z	z	Abund
371.2	1	23139.37
549.3	1	42951.14
553.2	1	63879.39
554.2	1	24076.97
652.3		97588.08
653.3	1	119554.73
654.2	1	42921.98
675.3	1	76776.57
676.2	1	34435.55
712.3	1	38095.04

Figure S3. HPLC-ESI spectra of compound **32.**

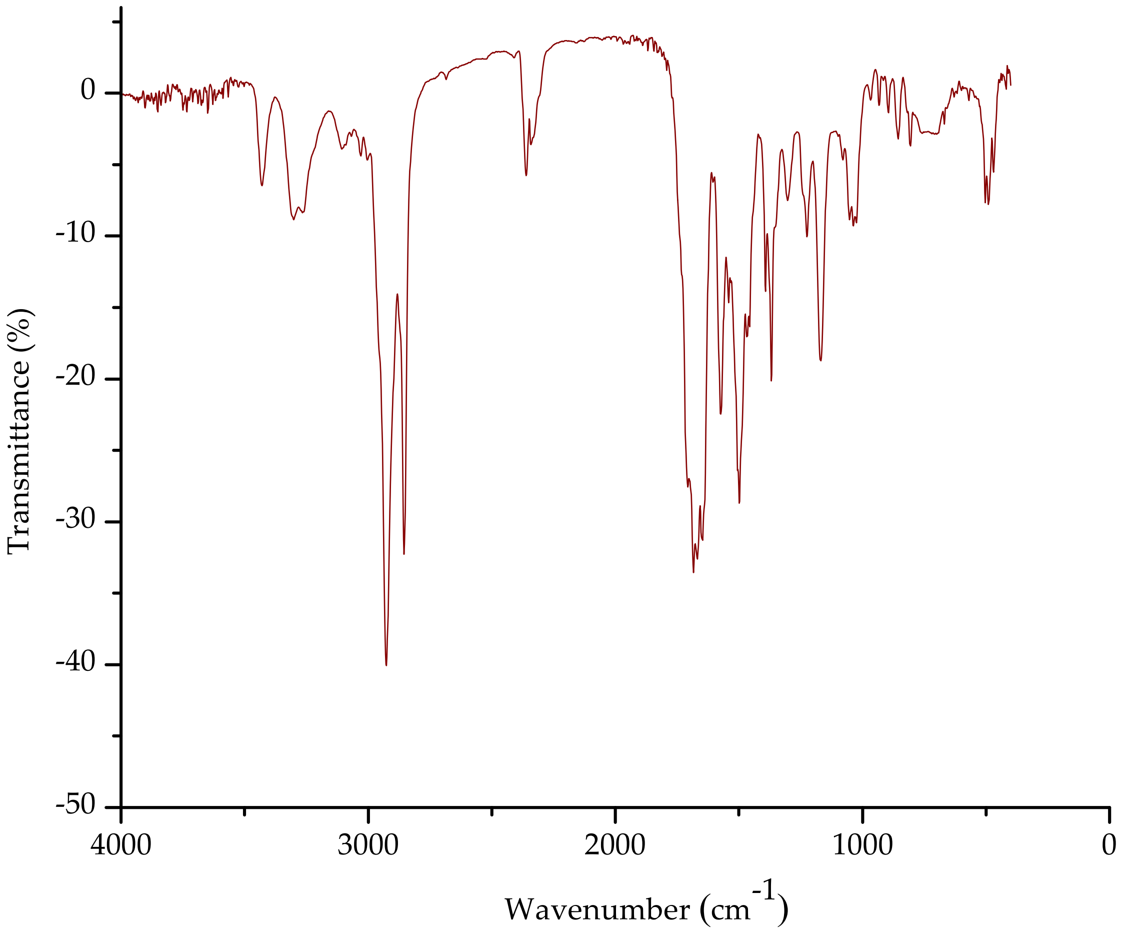

Figure S4. IR spectrum of compound **32** (c = 5 × 10^{-2} M) in DCM.

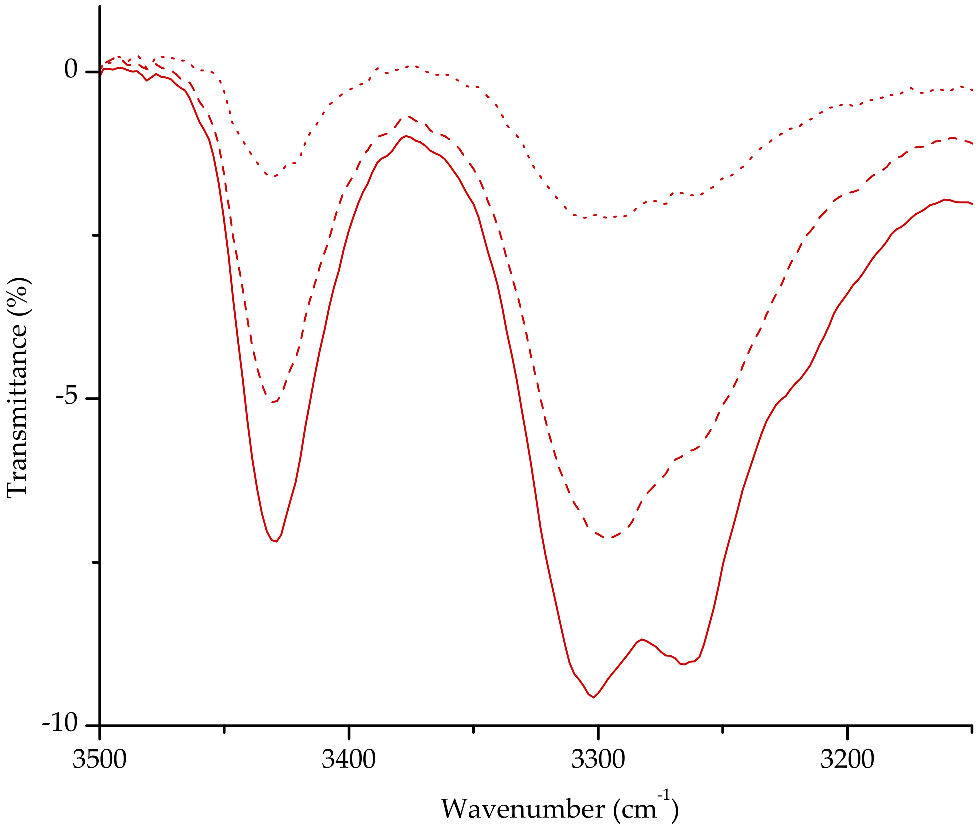

Figure S5. The NH stretching vibrations in concentration-dependent IR spectra of **32** in DCM [(—) $c = 5 \times 10^{-2}$ M, (——) $c = 2.5 \times 10^{-2}$ M, (· · ·) $c = 1.25 \times 10^{-2}$ M.

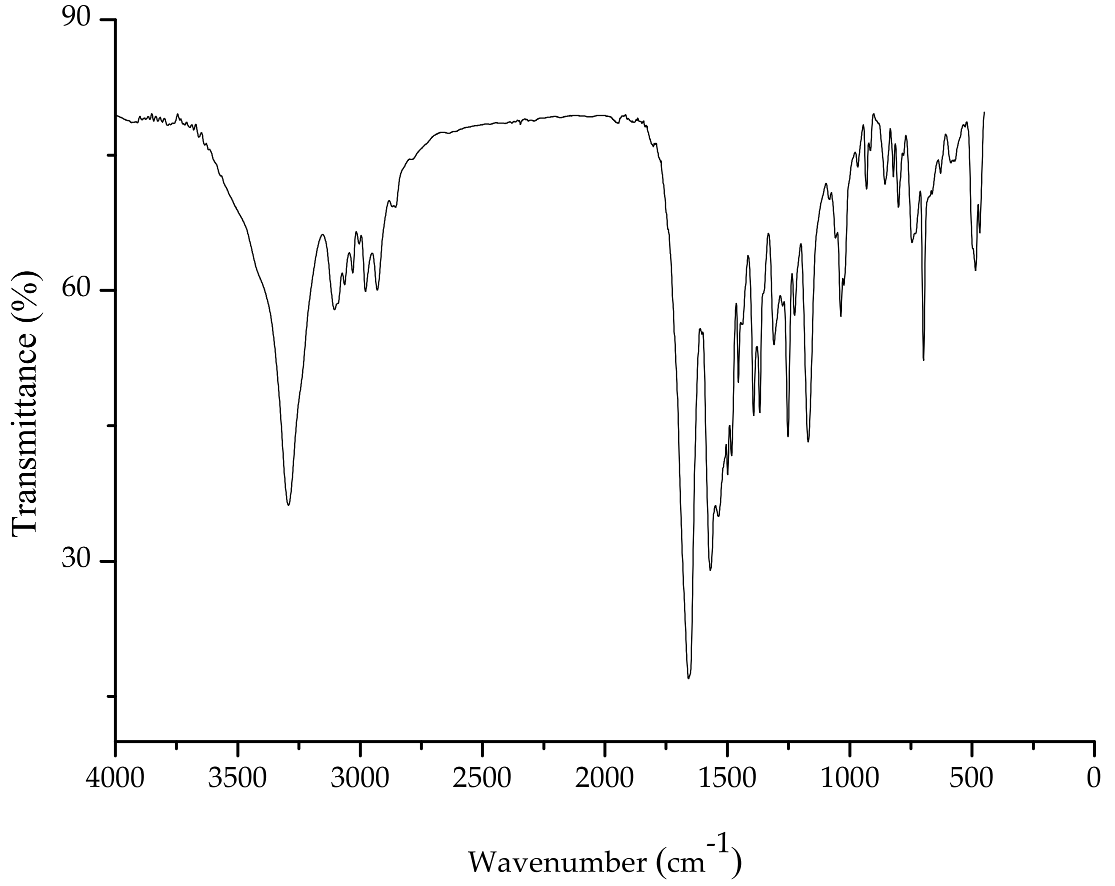

Figure S6. IR spectrum of compound **32** (2 mg) in KBr (200 mg).

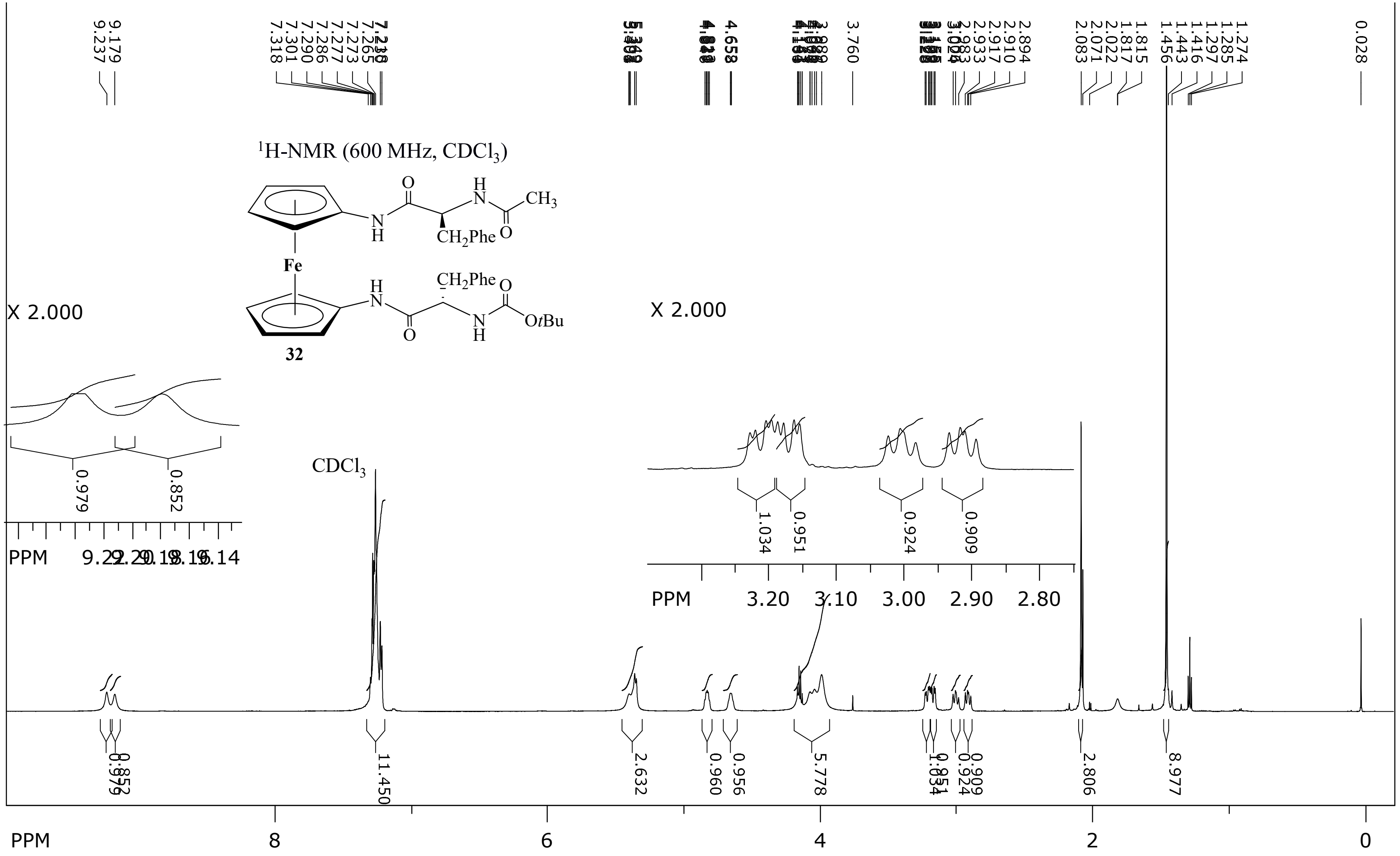

Figure S7. ^{1}H NMR spectrum of compound **32** ($c = 5 \times 10^{-2}$ M).

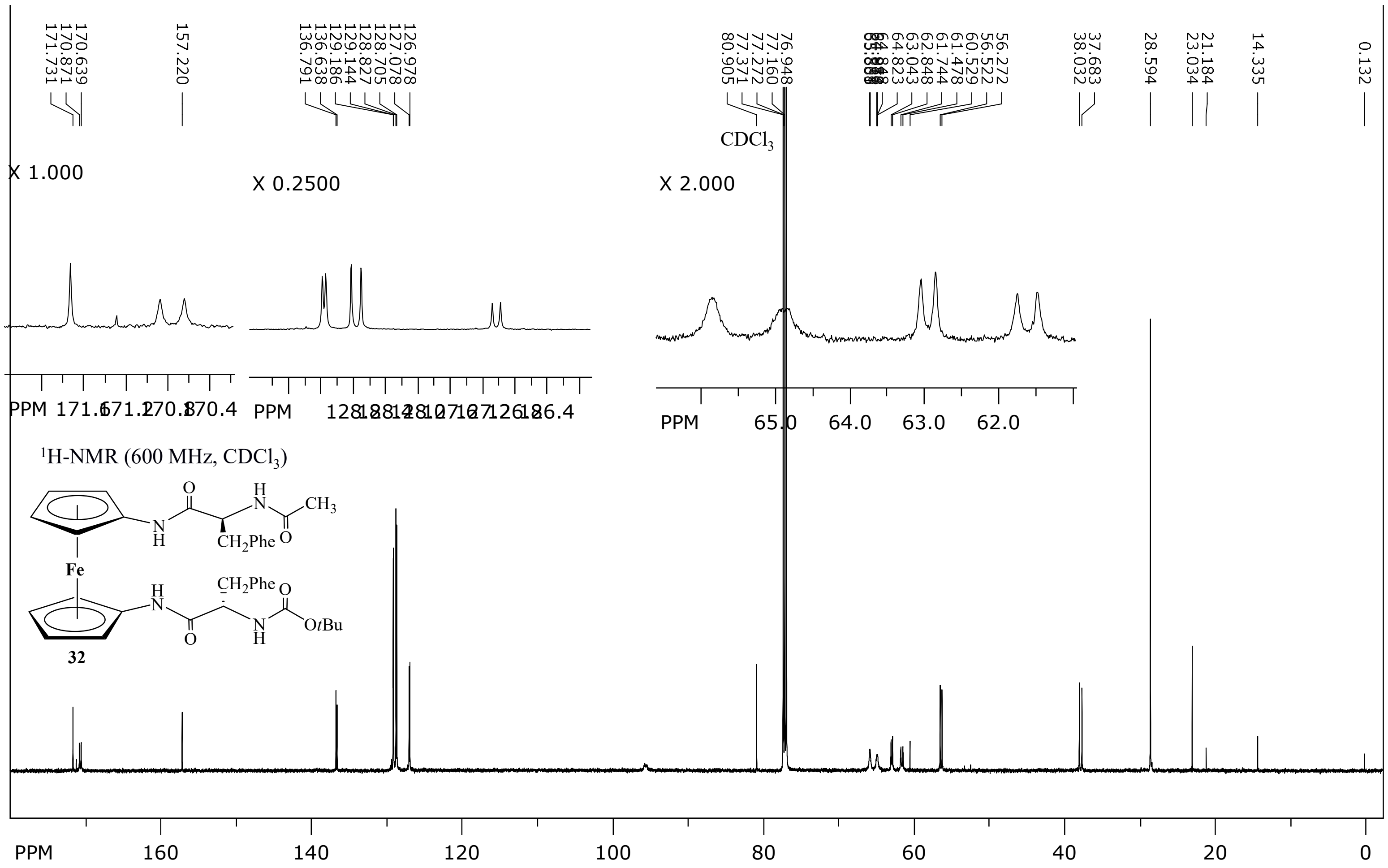

Figure S8. $^{13}C\{^{1}H\}$ NMR spectrum of compound **32** ($c = 5 \times 10^{-2}$ M).

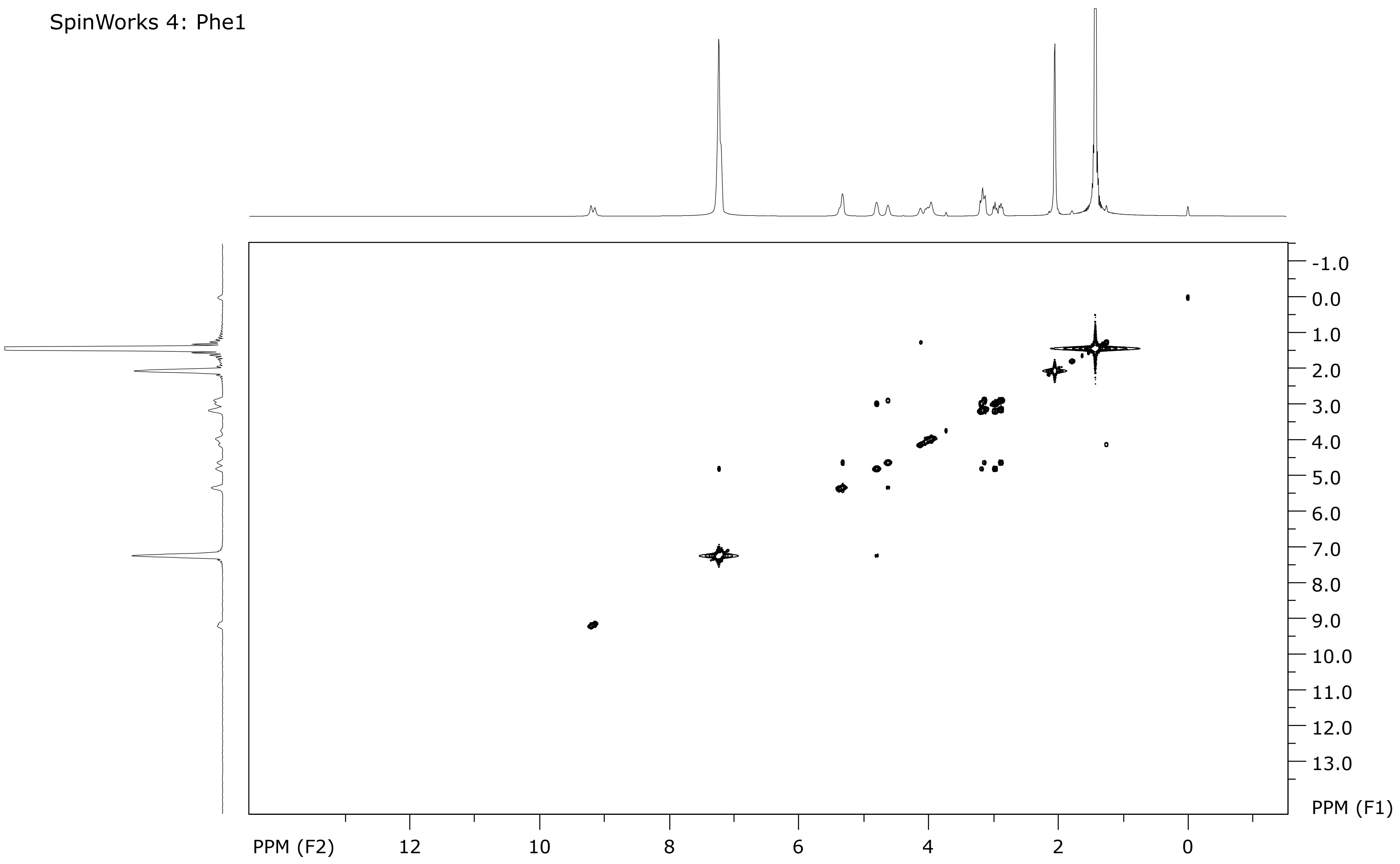

Figure S9. ^{1}H-^{1}H COSY NMR spectrum of compound **32** (c = 5 × 10^{-2} M).

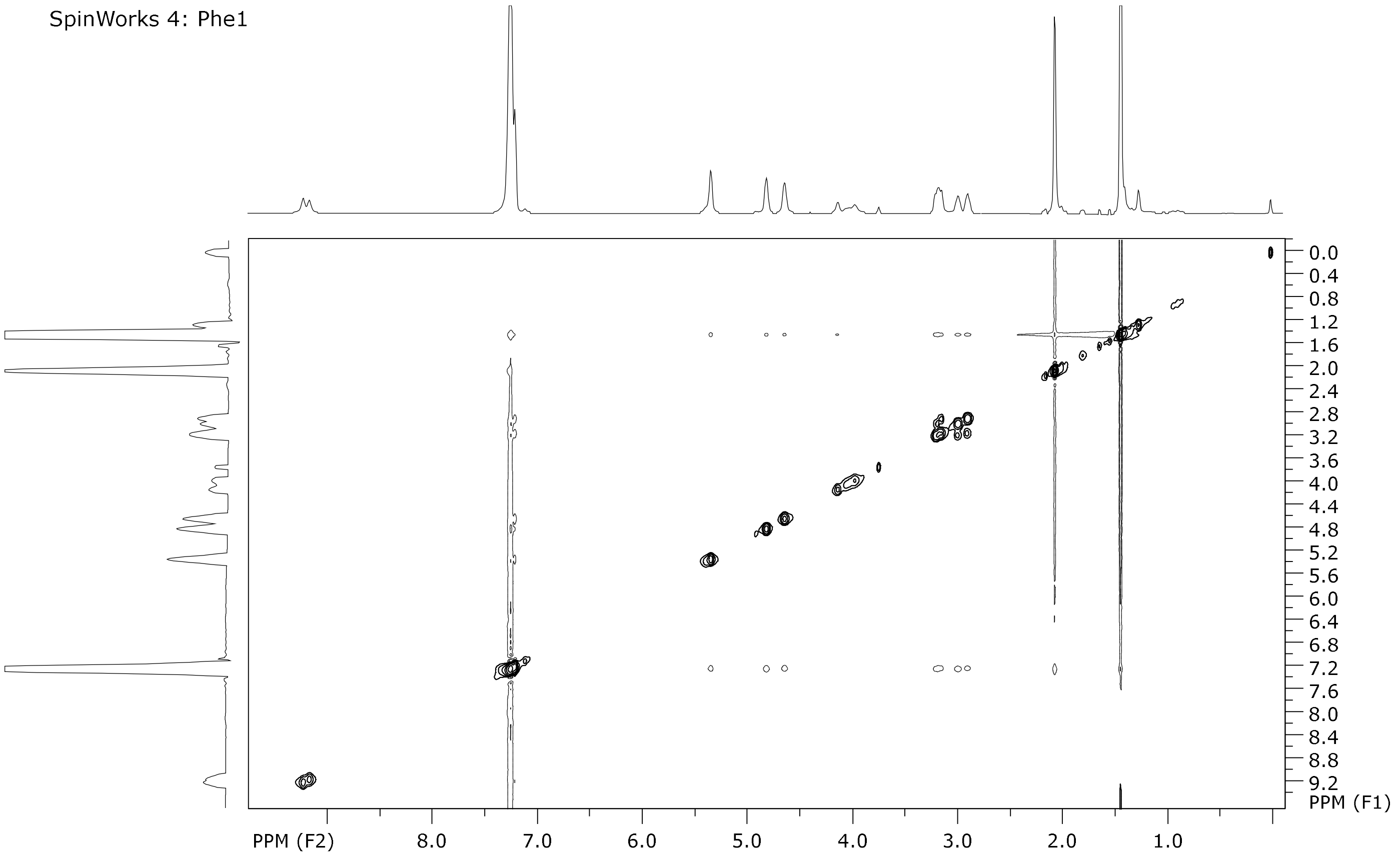

Figure S10. ^{1}H-^{1}H NOESY NMR spectrum of compound **32** ($c = 5 \times 10^{-2}$ M).

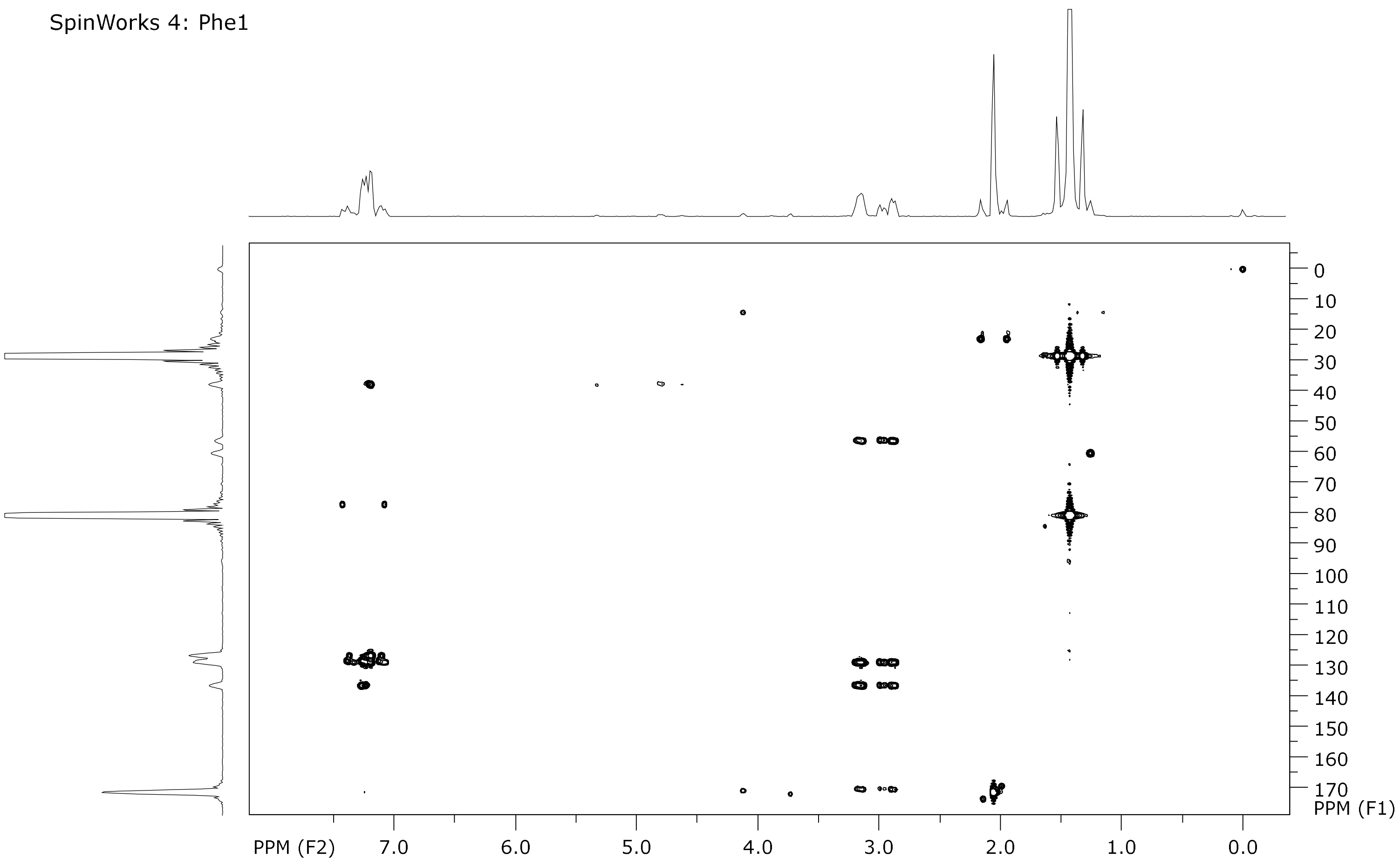

Figure S11. ^{1}H-^{13}C HMQC spectrum of compound **32** ($c = 5 \times 10^{-2}$ M).

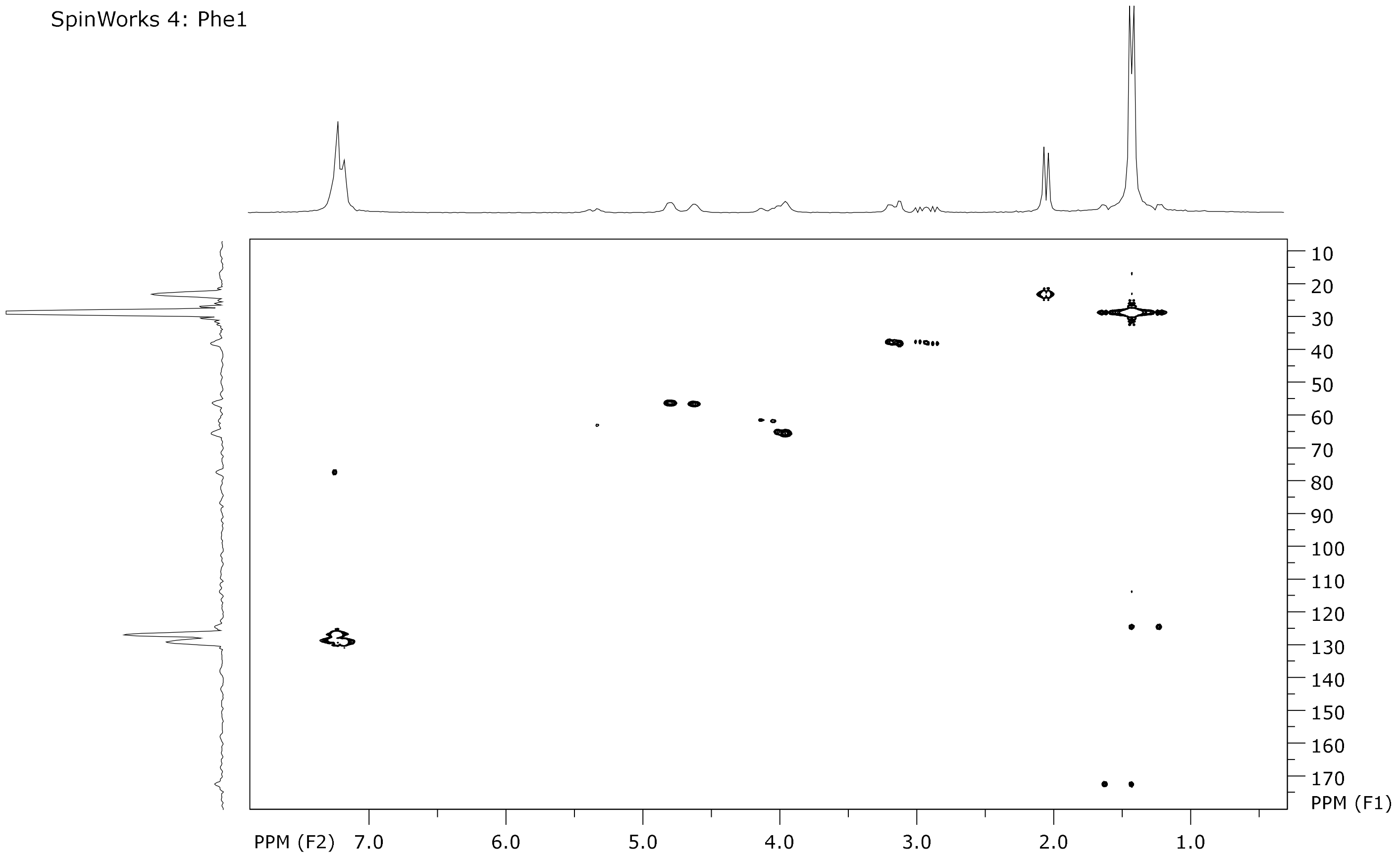

Figure S12. ^{1}H-^{13}C HMBC spectrum of compound **32** ($c = 5 \times 10^{-2}$ M).

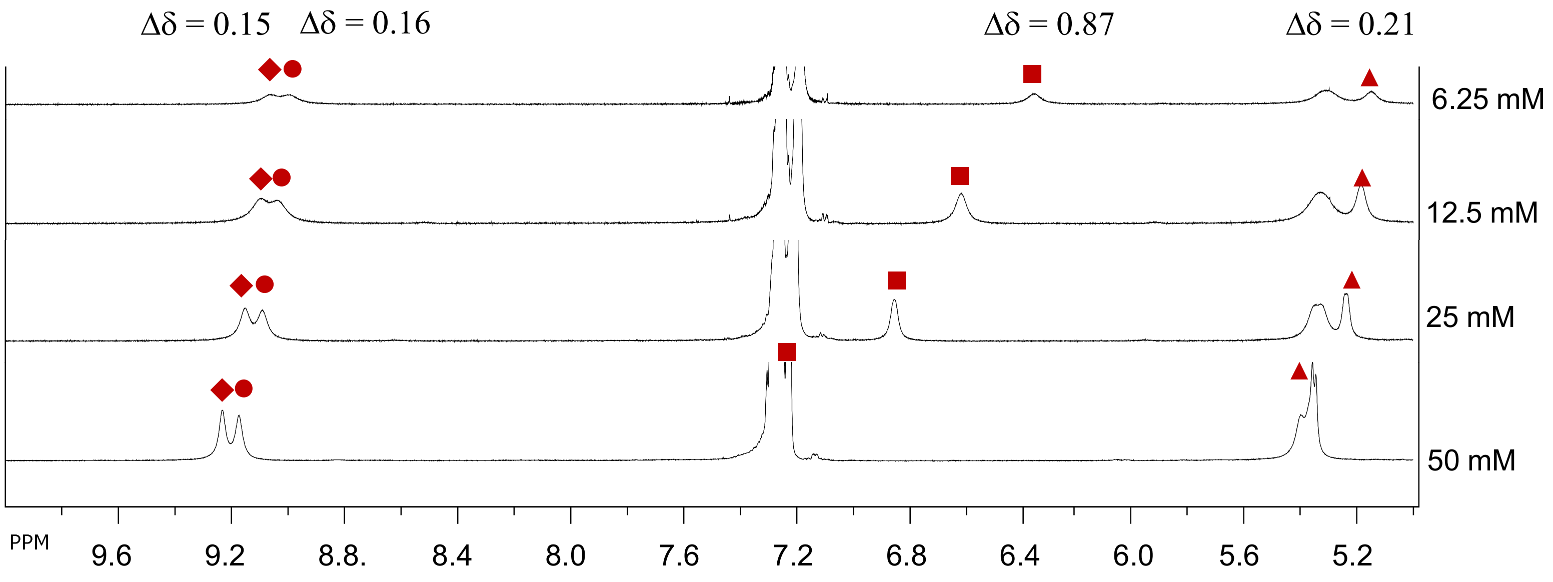

Figure S13. Concentration-dependent NH chemical shifts of compound **32** in $CDCl_3$.

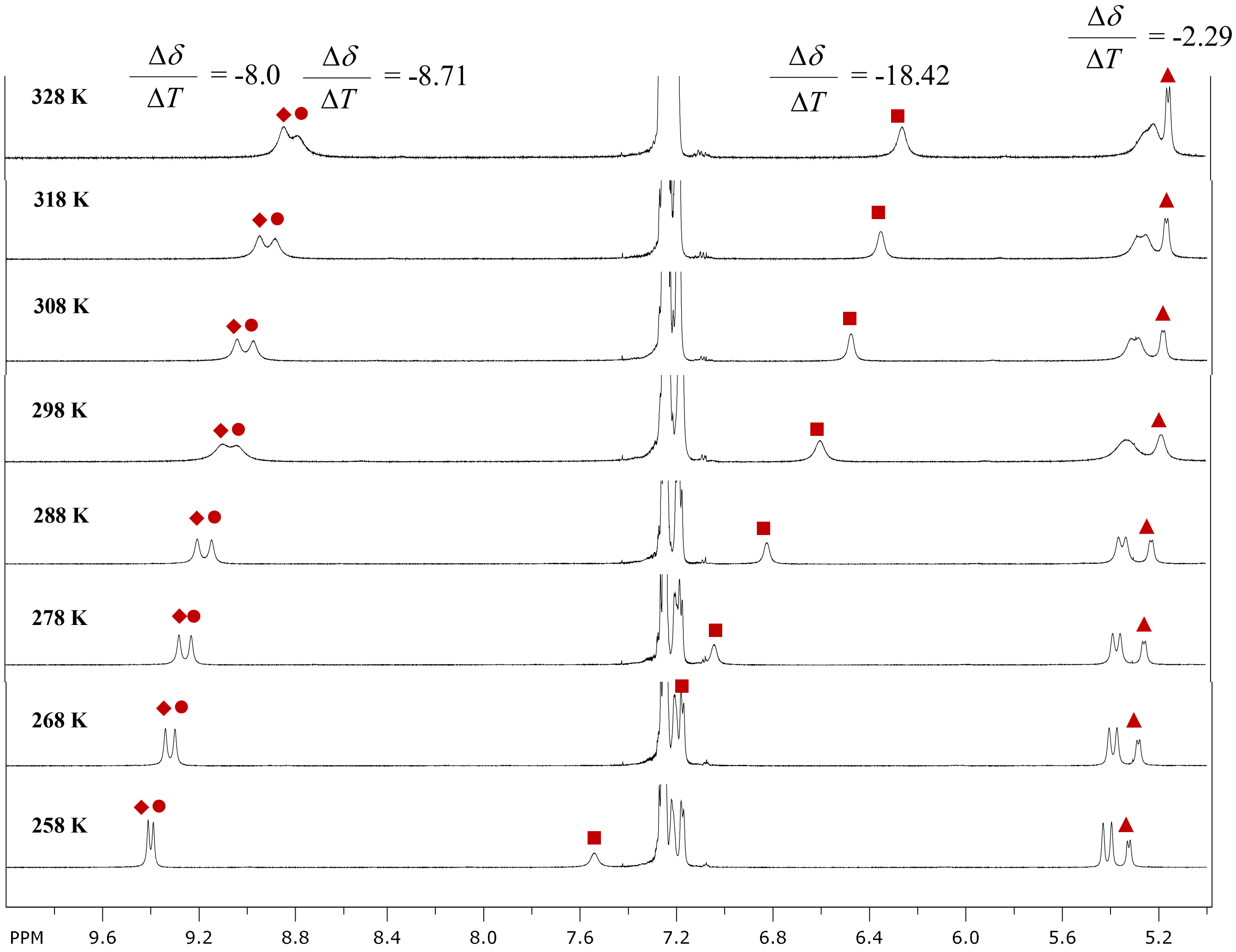

Figure S14. Temperature-dependent NH chemical shifts of compound **32** ($c = 1 \times 10^{-2}$ M).

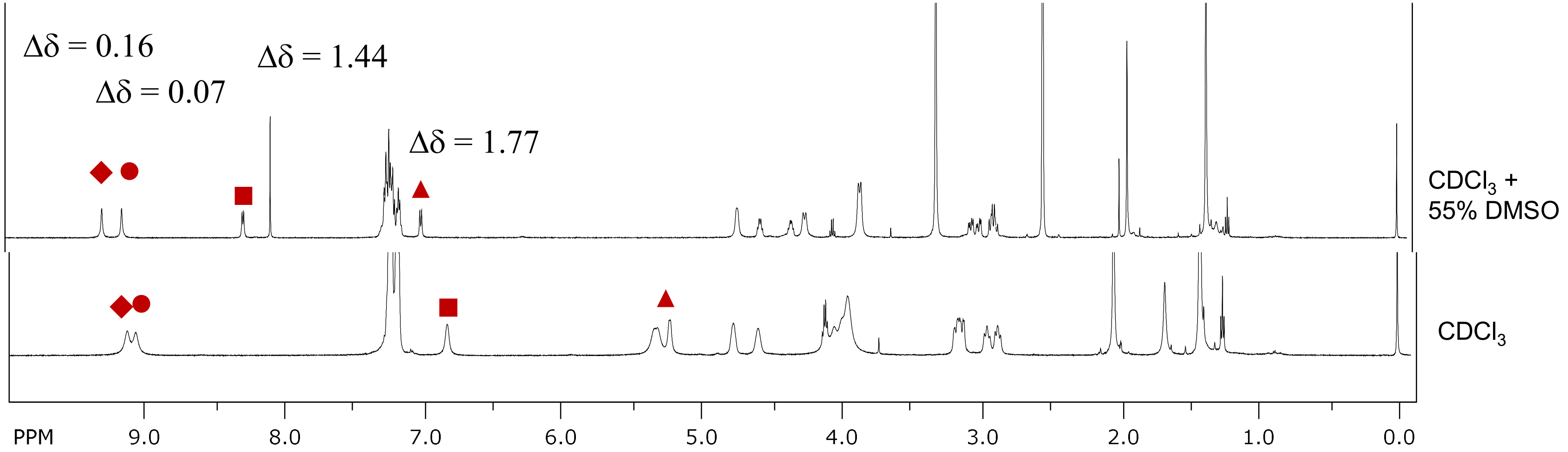

Figure S15. ^{1}H NMR spectra of compound **32** at varying concentrations of DMSO in $CDCl_3$ ($c = 2.5 \times 10^{-2}$ M).

Ac–D–Phe–NH–Fn–NH–D–Phe–Boc (35)

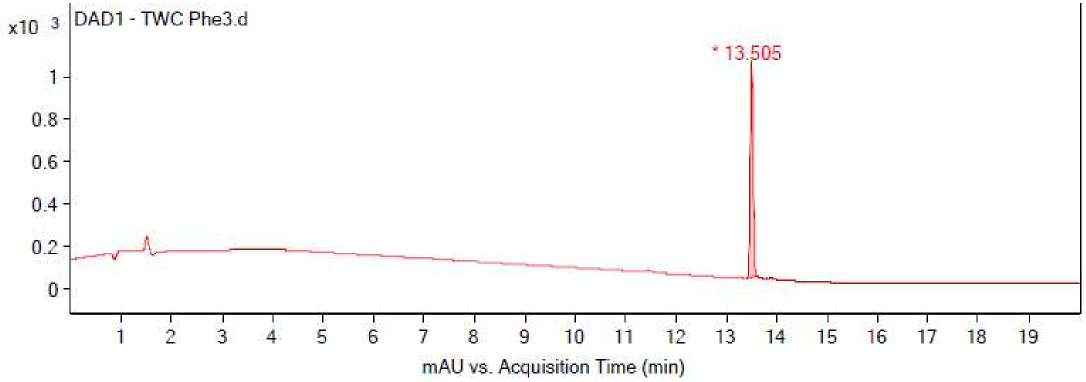

Integration Peak List

Peak	Start	RT	End	Height	Area	Area %
1	13.398	13.505	13.625	1015.37	3759.71	100

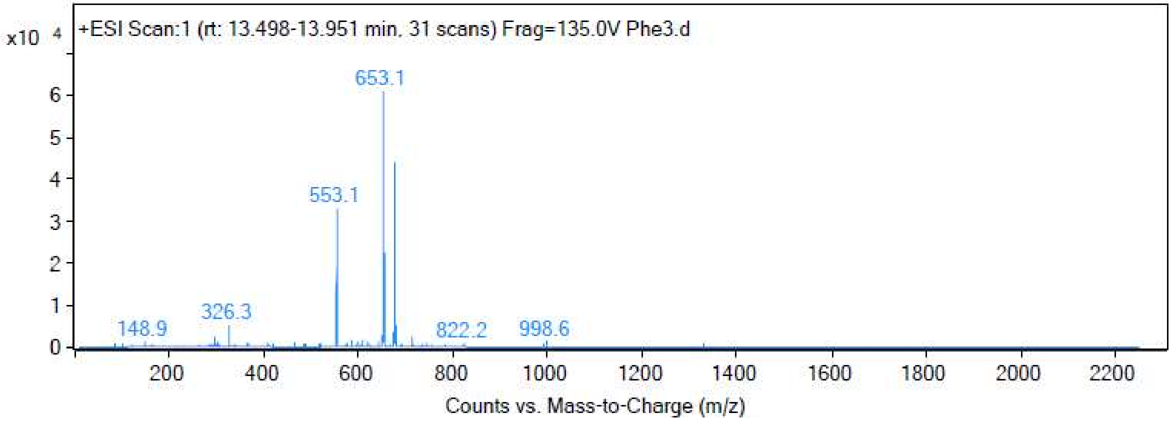

Peak List

m/z	z	Abund
326.3	1	5074.83
553.1	1	32871.92
554.1	1	12461.7
652.1		51132.72
653.1	1	60920.72
654.1	1	22339.01
655.1	1	4982.88
675.1	1	44003.48
676.1	1	18700.43
677.1	1	5240.64

Figure S16. HPLC-ESI spectra of compound **35.**

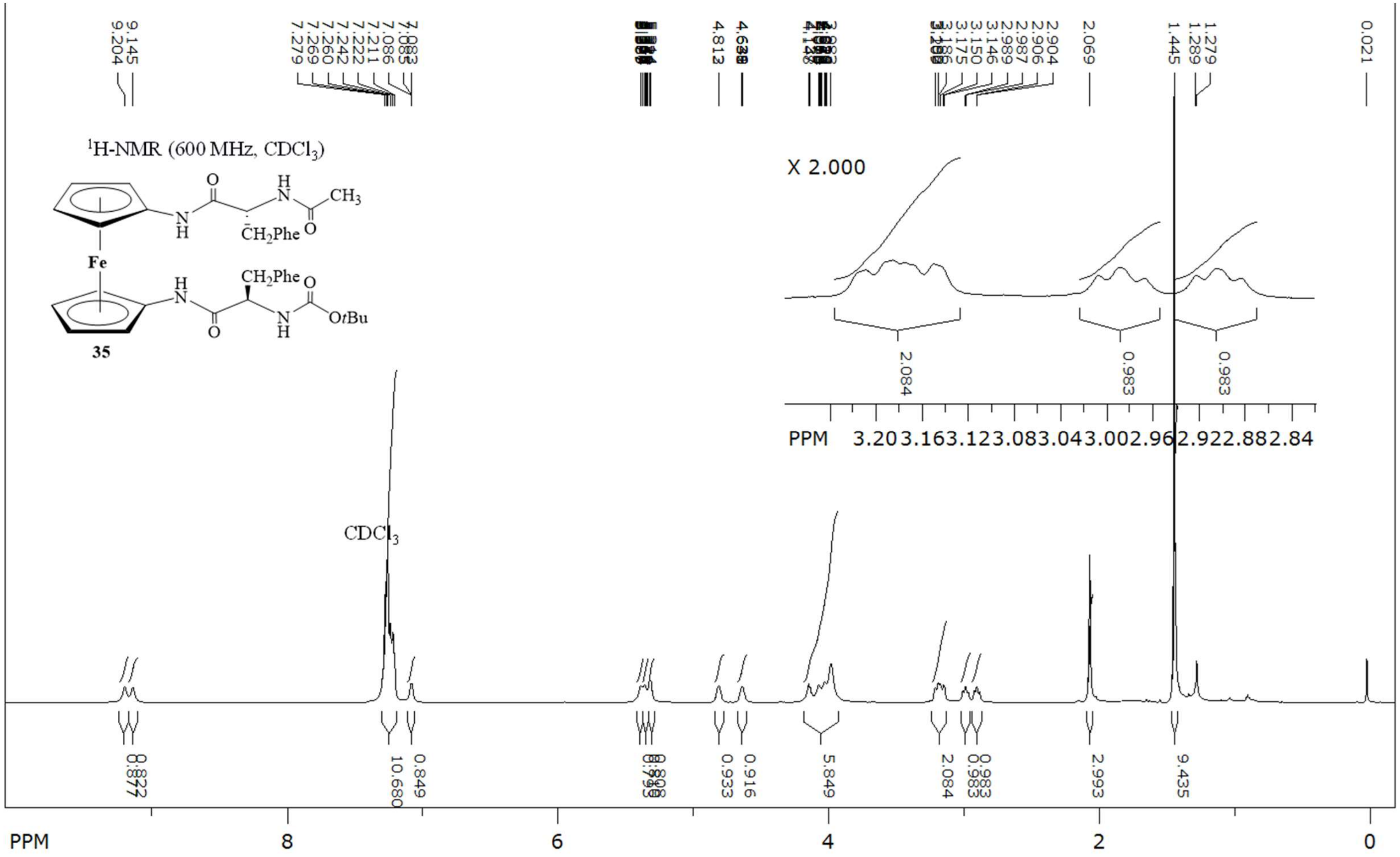

Figure S17. ^{1}H NMR spectrum of compound **35** ($c = 5 \times 10^{-2}$ M).

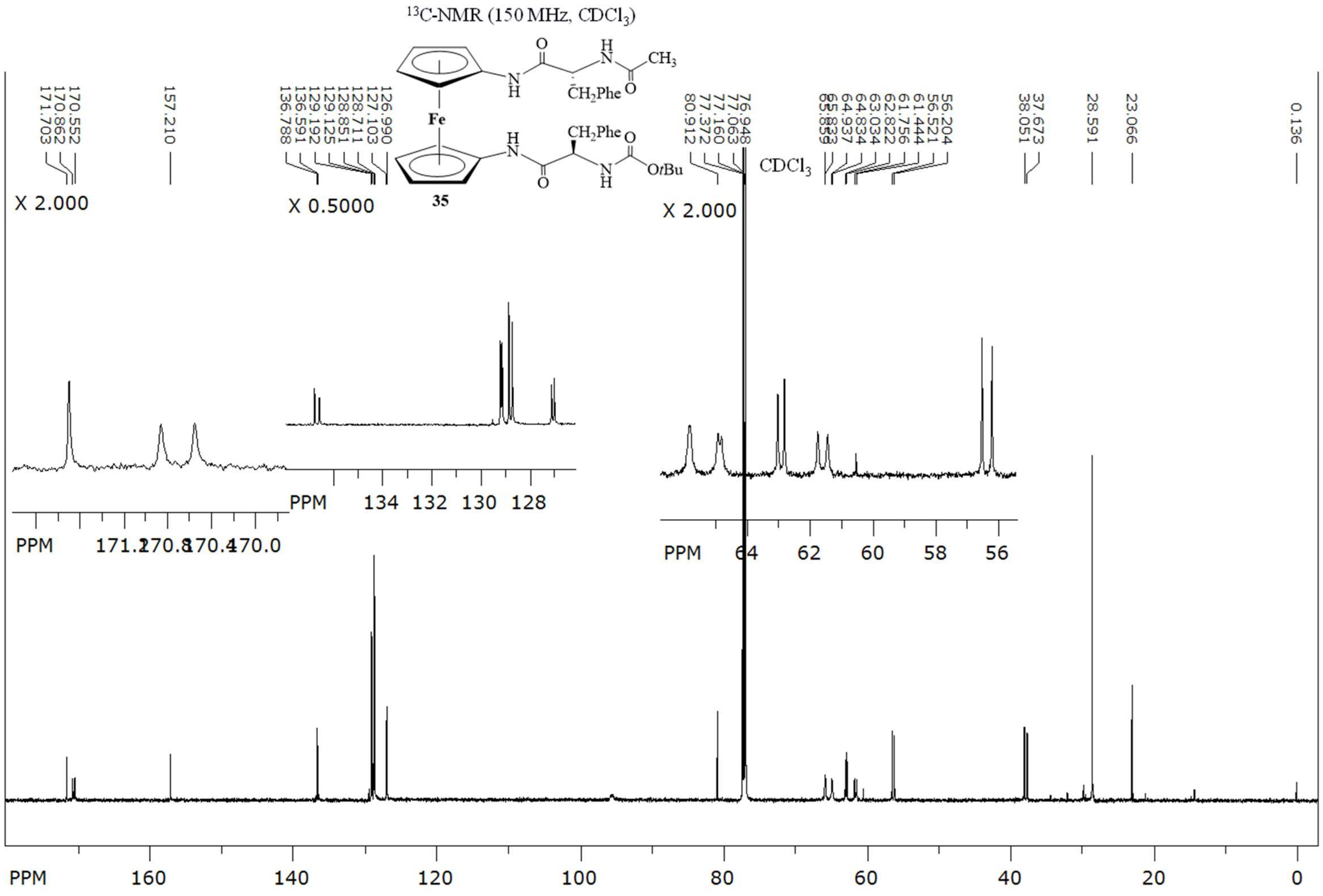

Figure S18. $^{13}C\{^{1}H\}$ NMR spectrum of compound **35** ($c = 5 \times 10^{-2}$ M).

Ac–L–Val–NH–Fn–NH–L–Val–Boc (33)

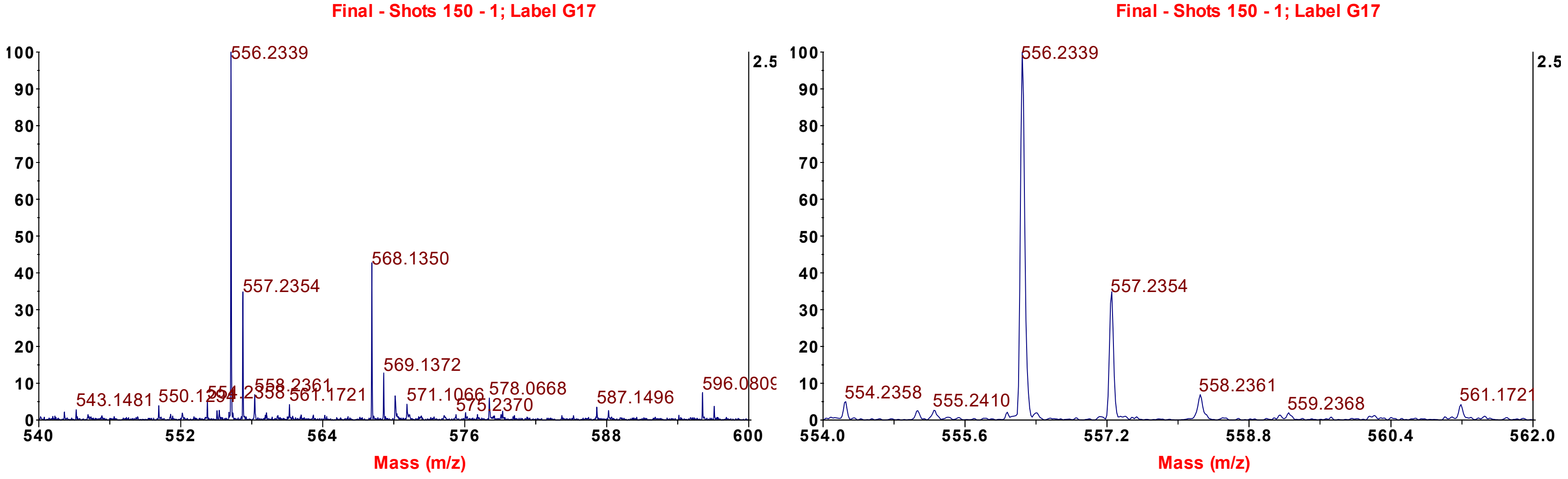

Figure S19. HRMS spectrum of compound **33.**

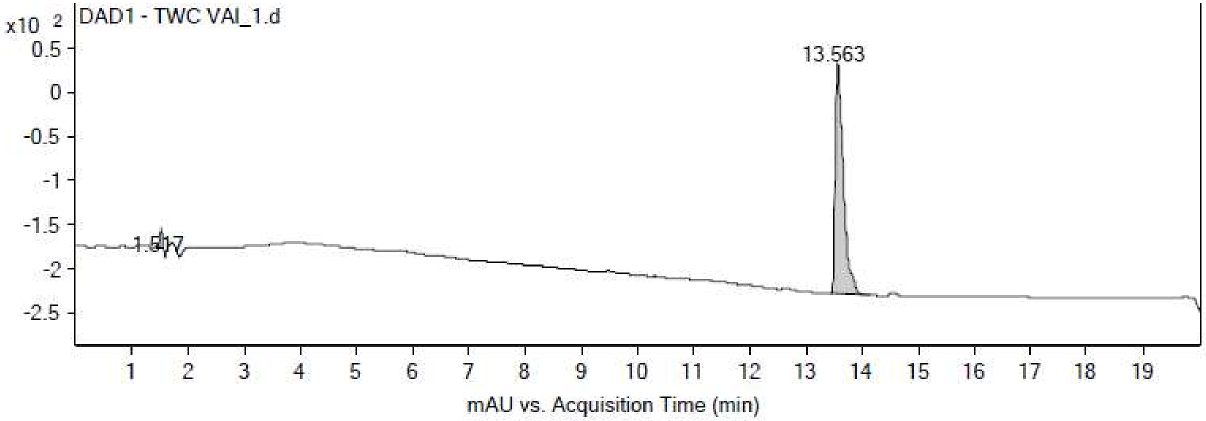

Integration Peak List

Peak	Start	RT	End	Height	Area	Area %
1	1.43	1.517	1.555	21.23	65.66	2.53
2	13.43	13.563	14.126	261.36	2593.39	100

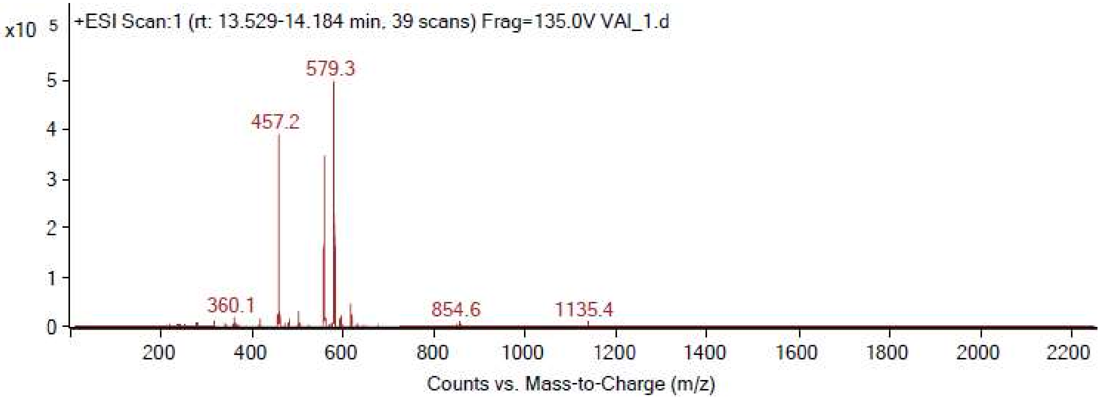

Peak List

m/z	z	Abund
457.2	1	387985.22
458.2	1	99976.73
556.3		176509.63
557.3	1	346304.03
558.2	1	107409.55
577.2		34858.21
579.3	1	497104.09
580.3	1	164582.95
581.3	1	32016.14
616.3	1	46668.61

Figure S20. HPLC-ESI spectra of compound **33.**

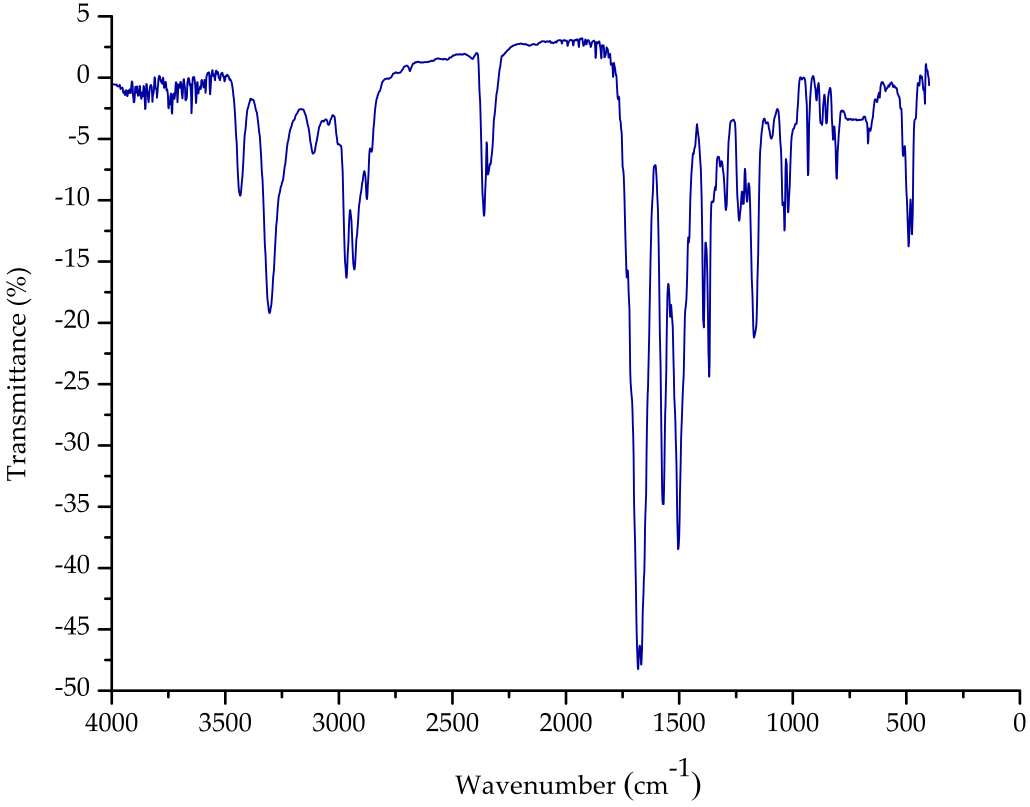

Figure S21. IR spectrum of compound **33** ($c = 5 \times 10^{-2}$ M) in DCM.

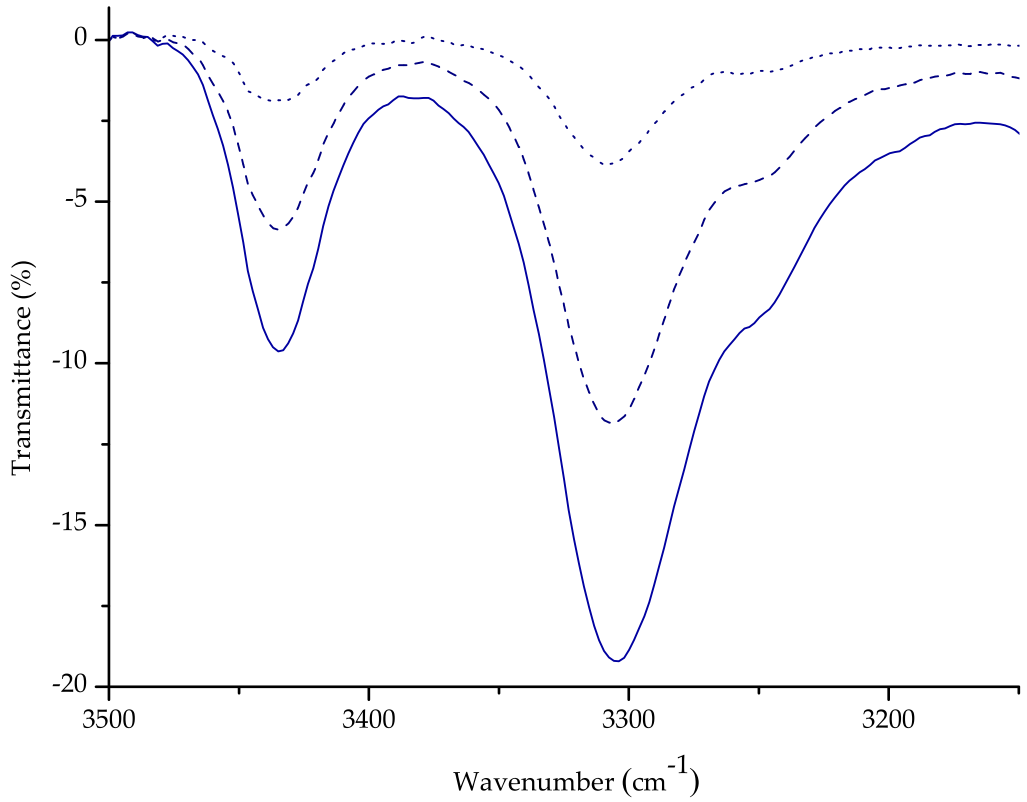

Figure S22. The NH stretching vibrations in concentration-dependent IR spectra of **33** in DCM [(—) $c = 5 \times 10^{-2}$ M, (———) $c = 2.5 \times 10^{-2}$ M, (· · ·) $c = 1.25 \times 10^{-2}$ M.

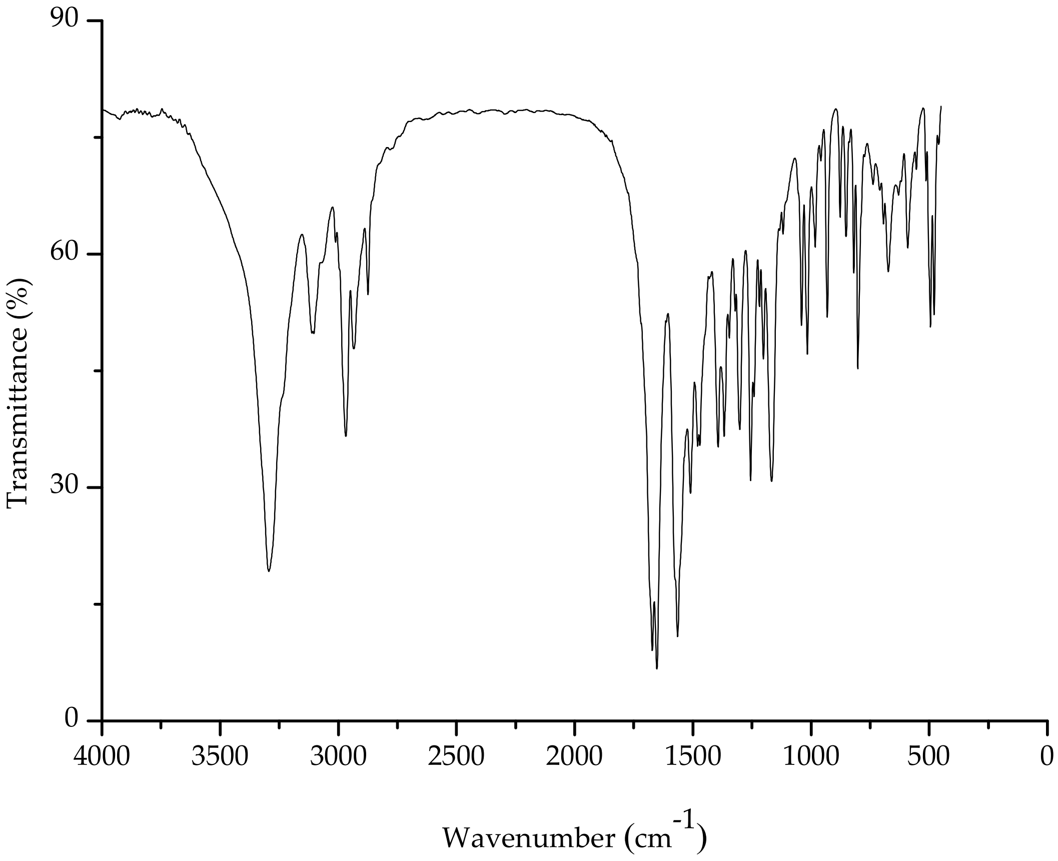

Figure S23. IR spectrum of compound **33** (2 mg) in KBr (200 mg).

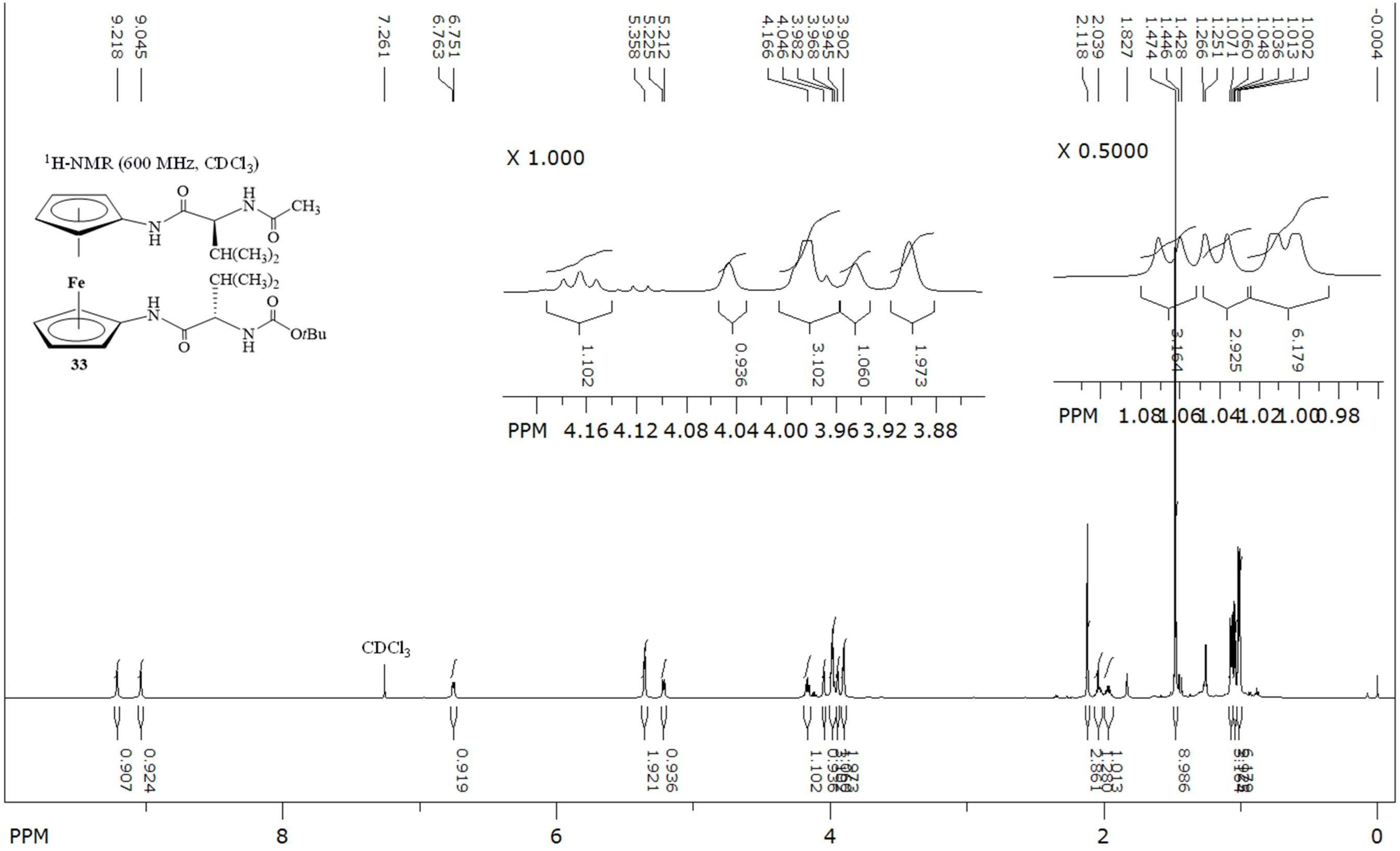

Figure S24. ^{1}H NMR spectrum of compound **33** ($c = 5 \times 10^{-2}$ M).

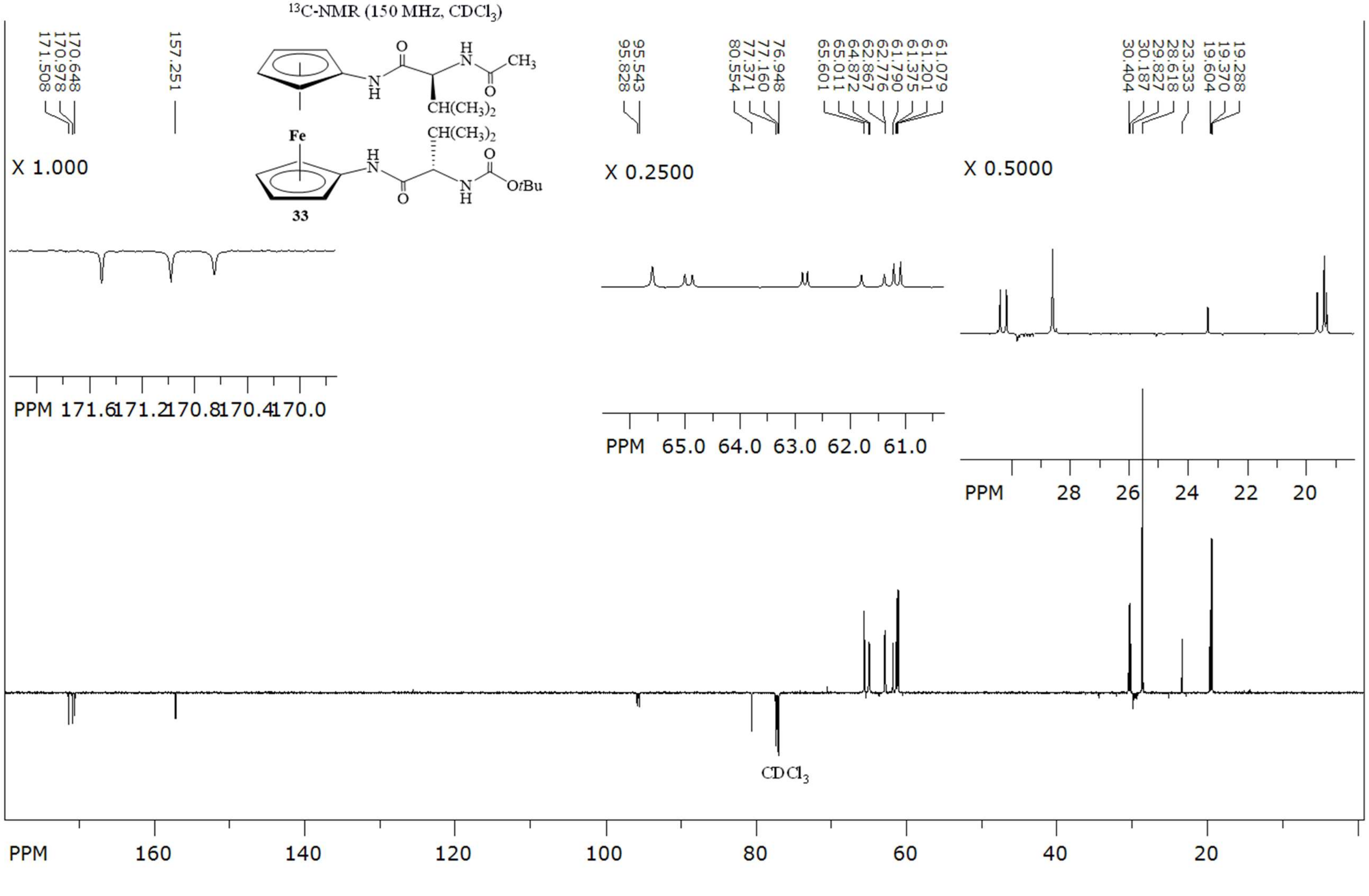

Figure S25. $^{13}C\{^{1}H\}$ NMR spectrum of compound **33** ($c = 5 \times 10^{-2}$ M).

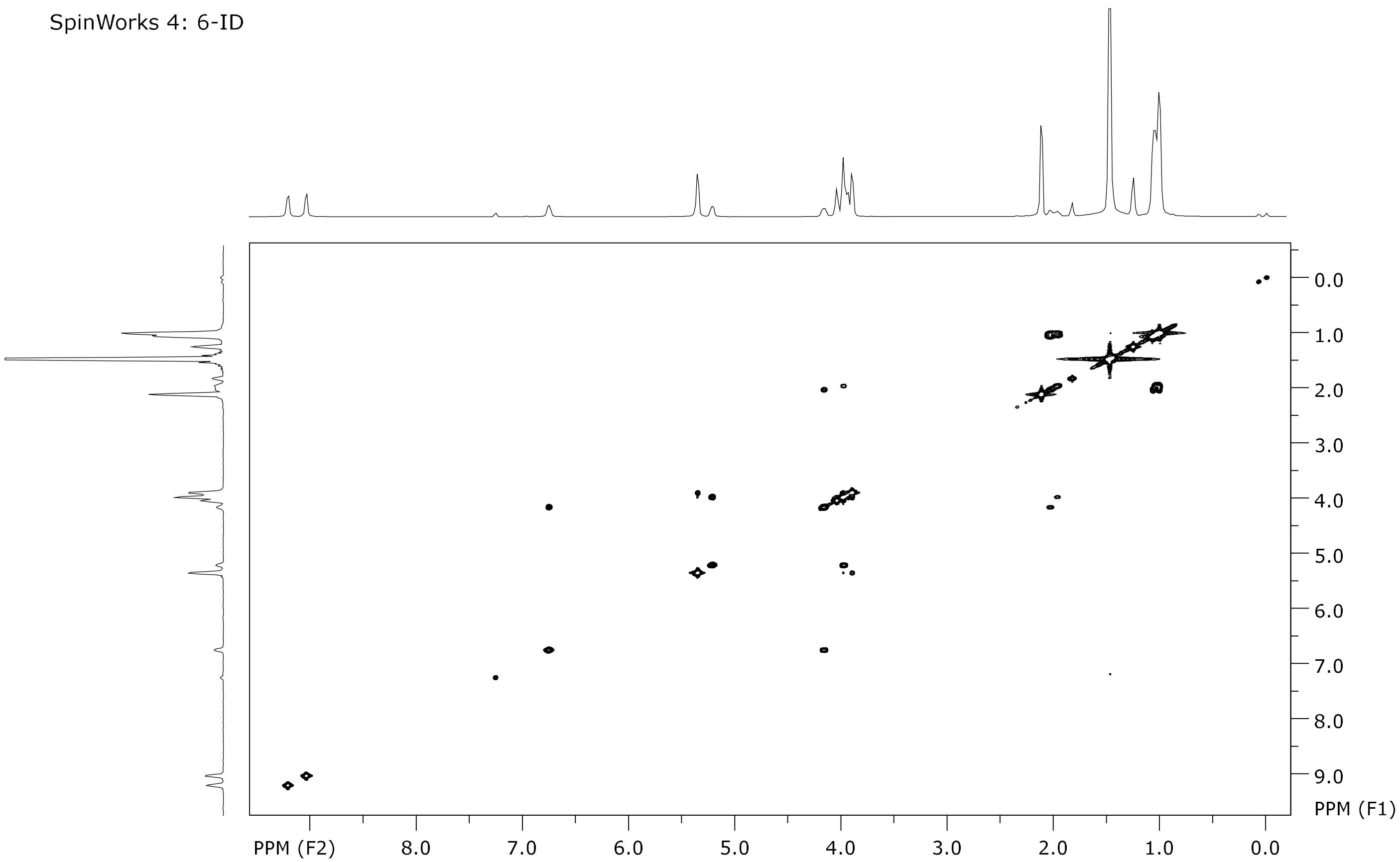

Figure S26. ^{1}H-^{1}H COSY NMR spectrum of compound **33** ($c = 5 \times 10^{-2}$ M).

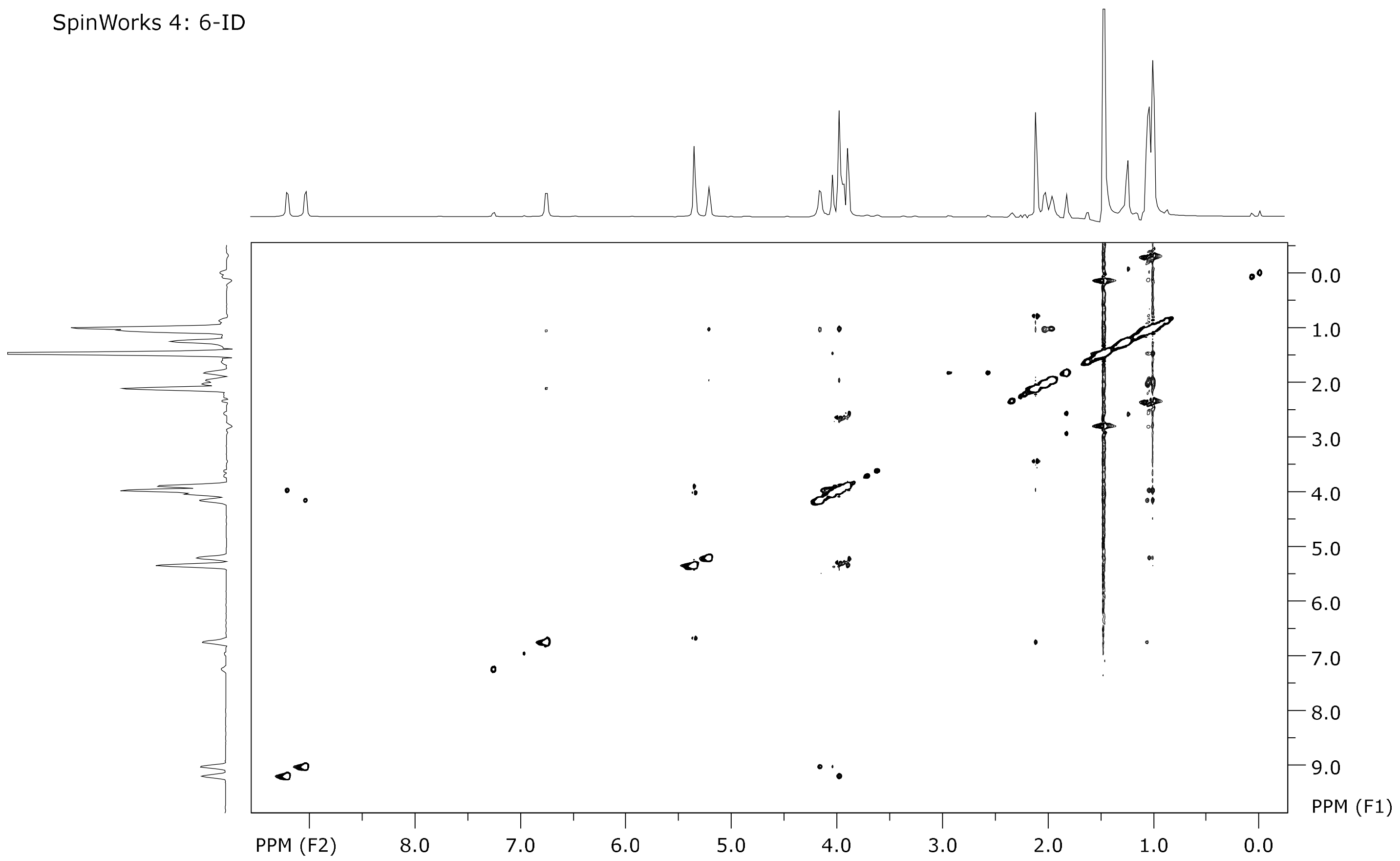

Figure S27. ^1H-^1H NOESY NMR spectrum of compound **33** ($c = 5 \times 10^{-2}$ M).

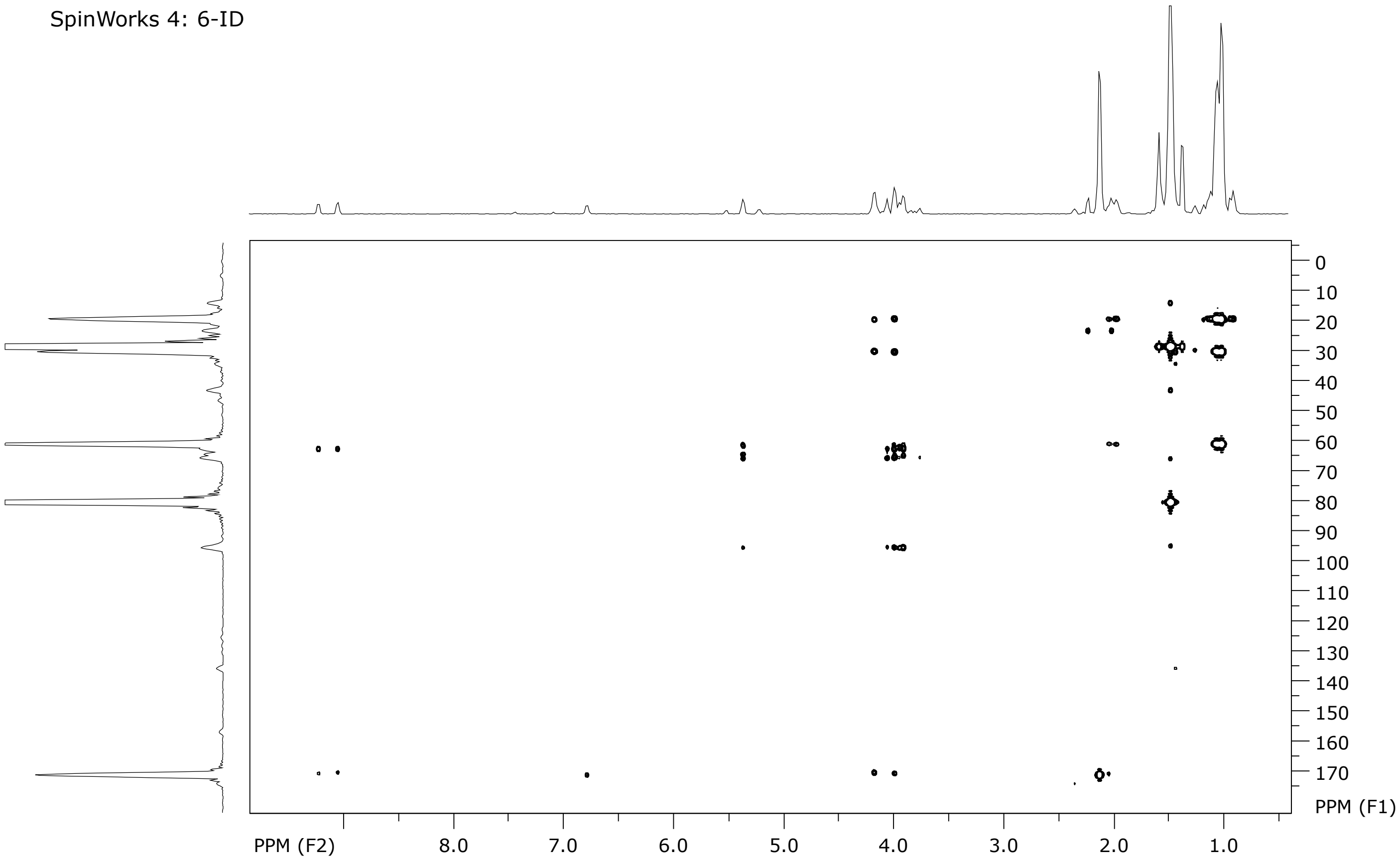

Figure S28. ^{1}H-^{13}C HMQC spectrum of compound **33** ($c = 5 \times 10^{-2}$ M).

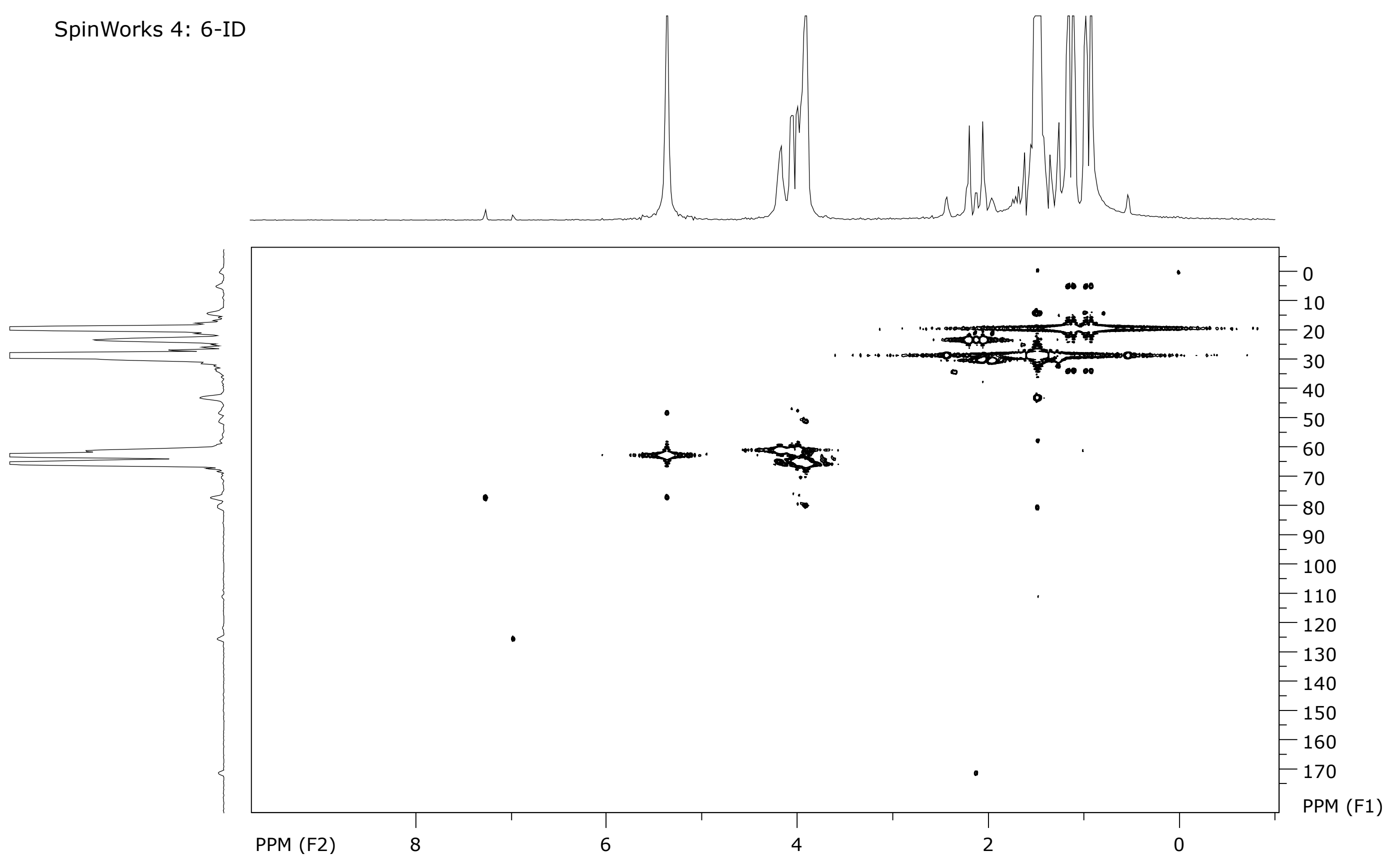

Figure S29. ^{1}H-^{13}C HMBC spectrum of compound **33** ($c = 5 \times 10^{-2}$ M).

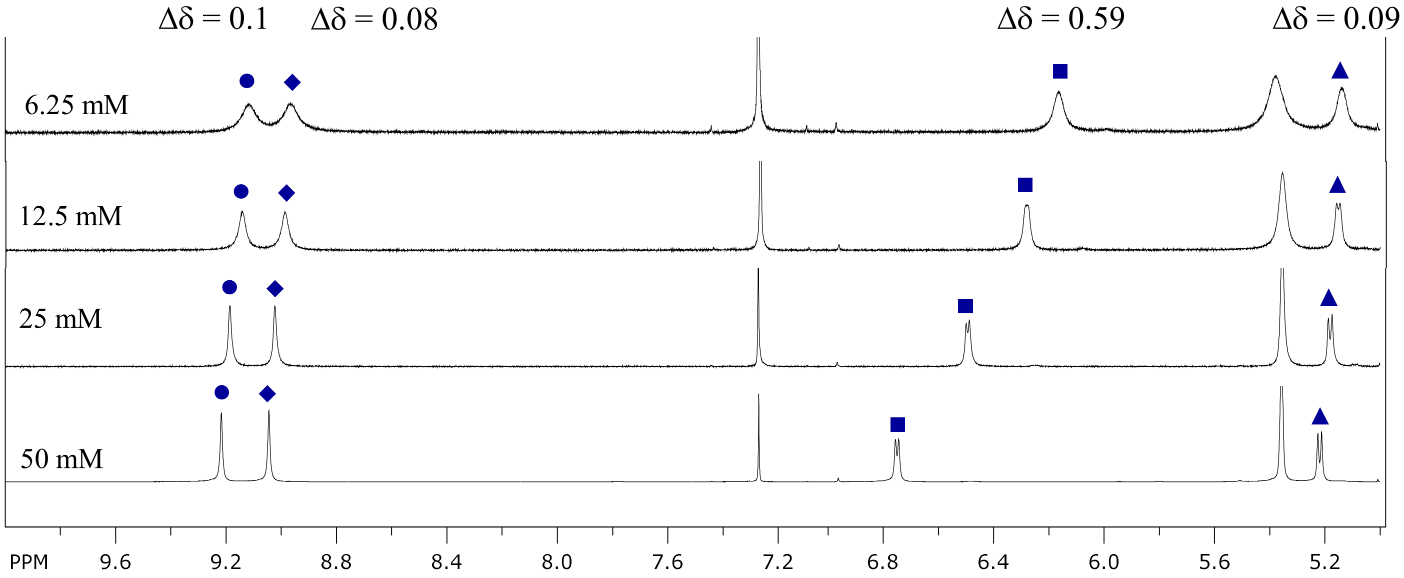

Figure S30. Concentration-dependent NH chemical shifts of compound **33** in $CDCl_3$.

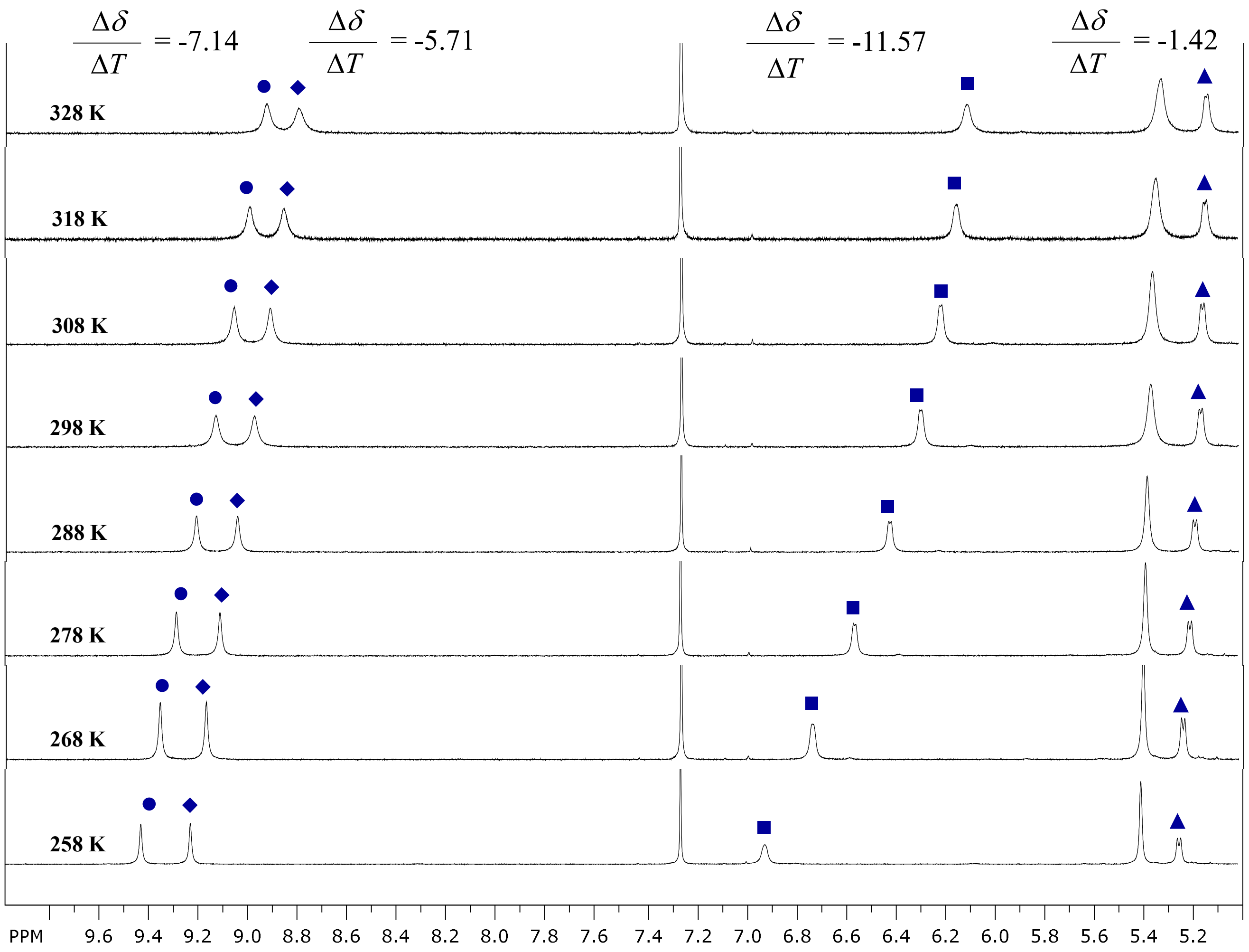

Figure S31. Temperature-dependent NH chemical shifts of compound **33** ($c = 1 \times 10^{-2}$ M).

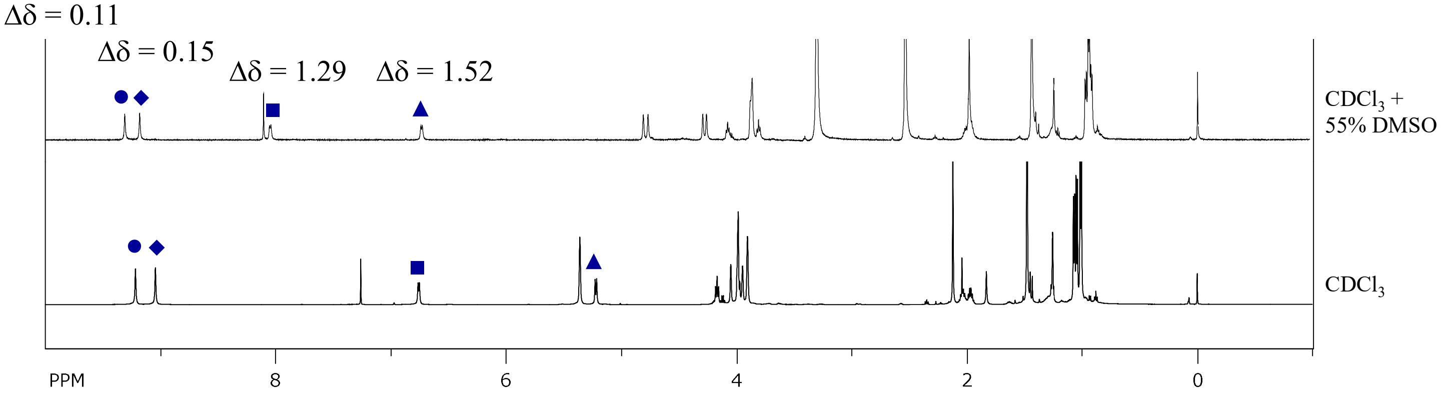

Figure S32. ^{1}H NMR spectra of compound **33** at varying concentrations of DMSO in $CDCl_3$ ($c = 2.5 \times 10\text{-}2$ M).

Ac–D–Val–NH–Fn–NH–D–Val–Boc (36)

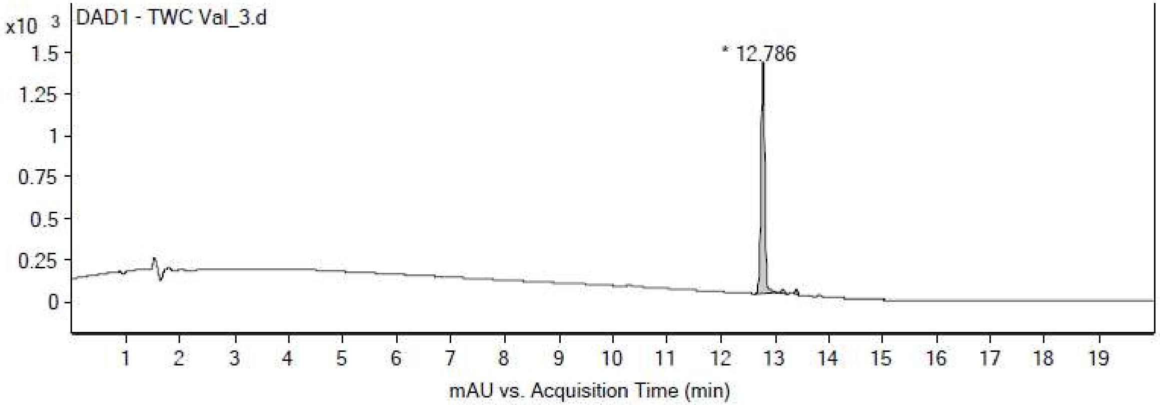

Integration Peak List

Peak	Start	RT	End	Height	Area	Area %
1	12.626	12.786	13.033	1388.83	7292.07	100
2	13.046	13.146	13.226	28.08	103.81	1.42
3	13.339	13.399	13.453	33.67	98.4	1.35

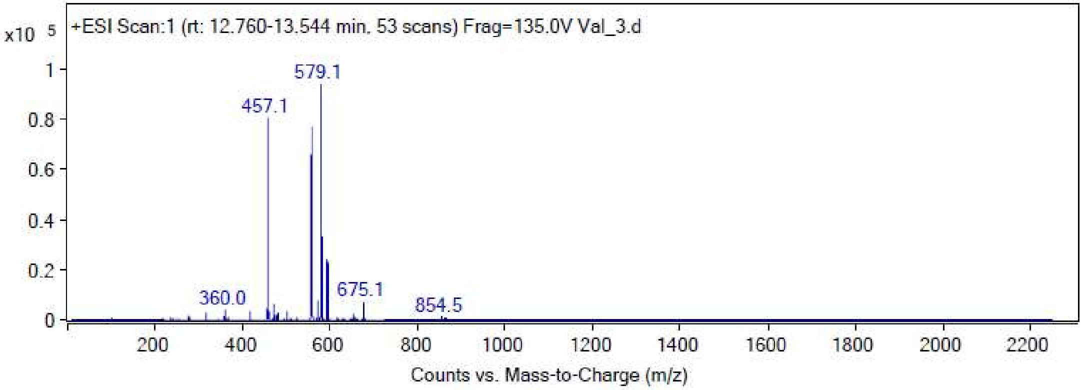

Peak List

m/z	z	Abund
457.1	1	80791.95
458.1	1	23177.79
556.1		66362.38
557.1	1	77432.63
558.1	1	24279.56
579.1	1	94219.45
580.1	1	33641.09
581.1	1	7447.52
593.1	1	23926.74
594.1	1	8303.44

Figure S33. HRMS spectrum of compound **36.**

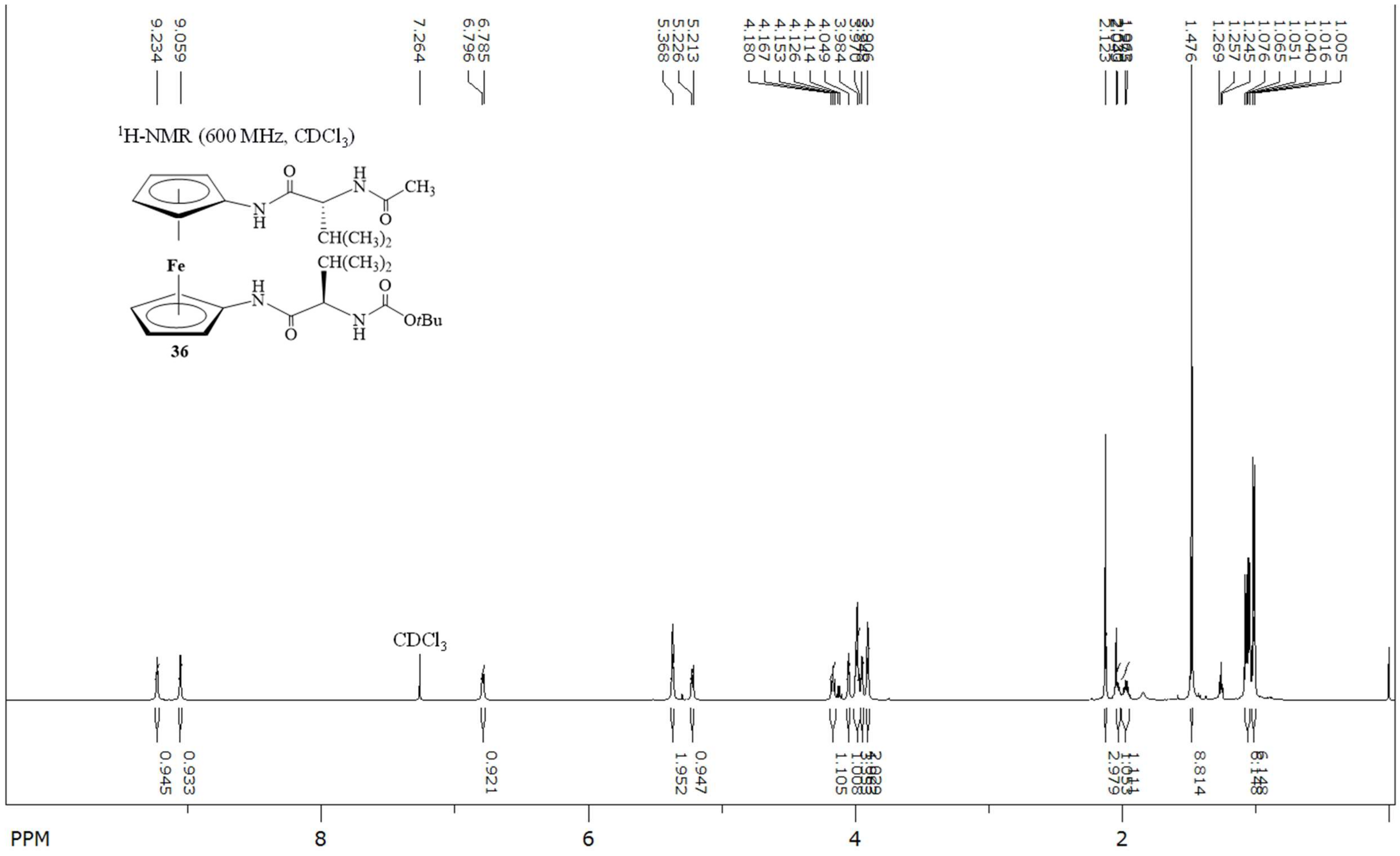

Figure S34. ^{1}H NMR spectrum of compound **36** ($c = 5 \times 10^{-2}$ M).

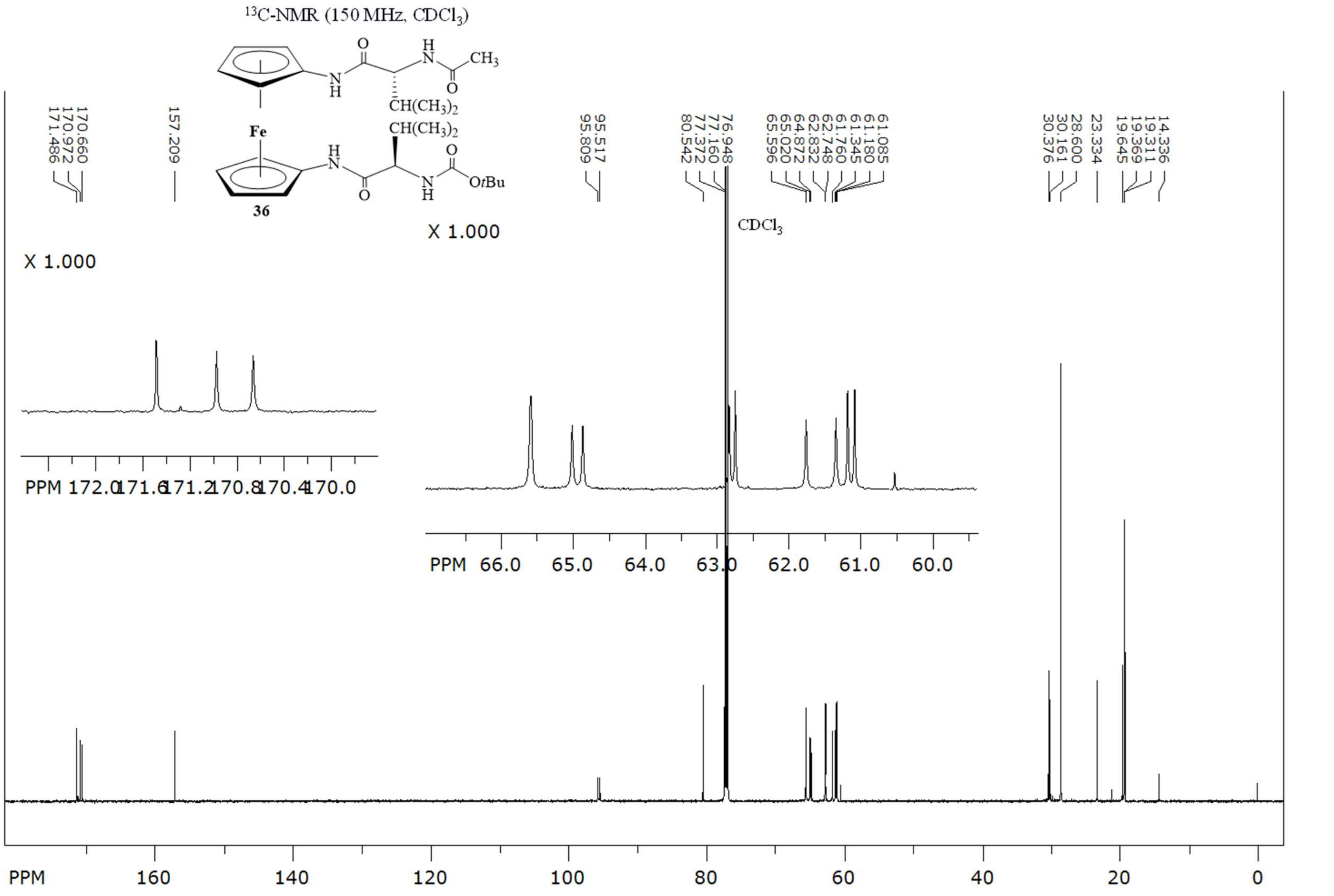

Figure S35. $^{13}C\{^1H\}$ NMR spectrum of compound **36** ($c = 5 \times 10^{-2}$ M).

Ac–L–Leu–NH–Fn–NH–L–Leu–Boc (34)

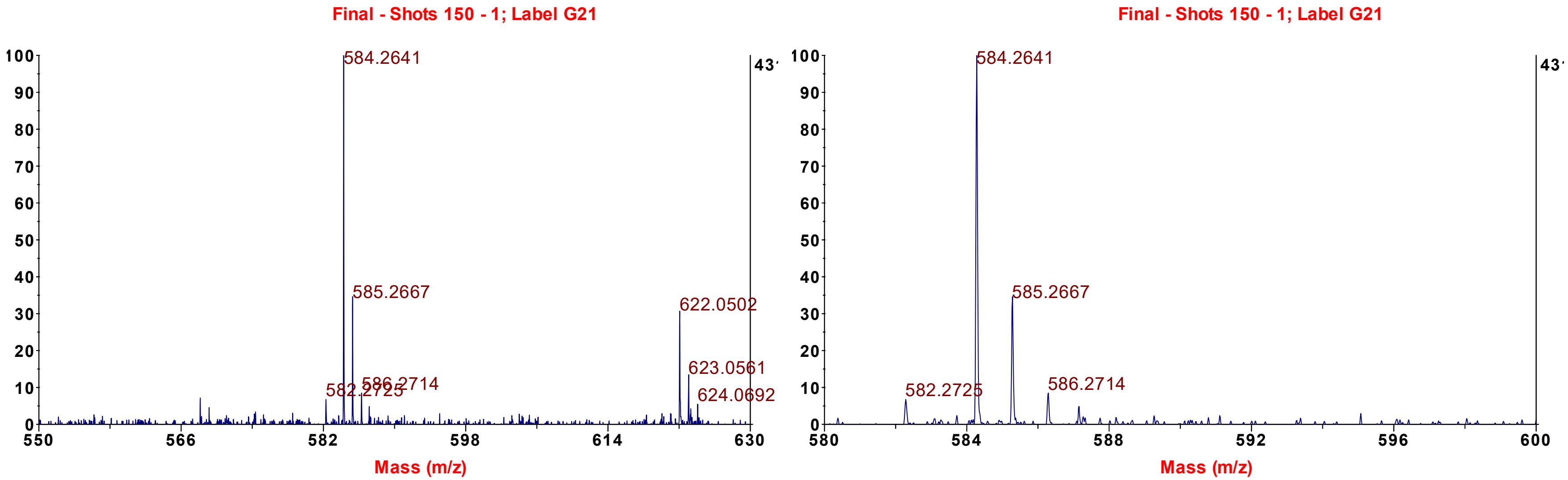

Figure S36. HRMS spectrum of compound **34.**

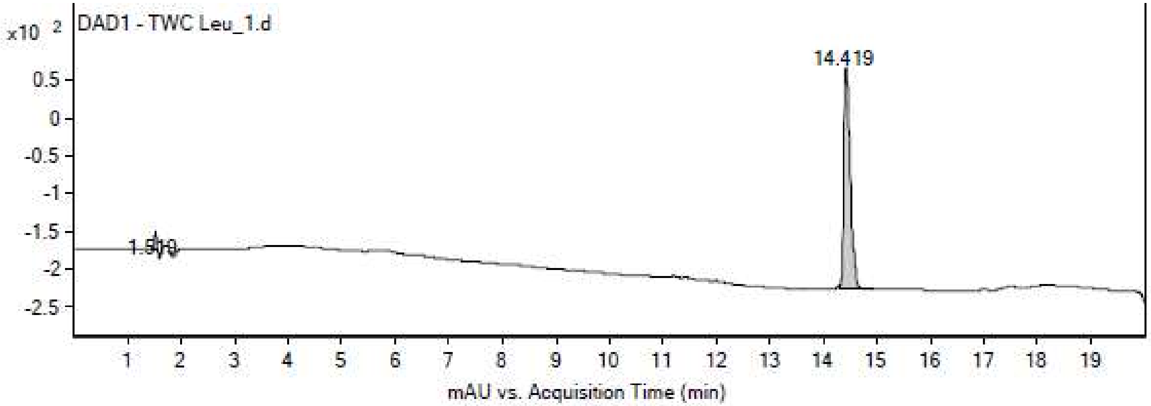

Integration Peak List

Peak	Start	RT	End	Height	Area	Area %
1	1.426	1.519	1.557	22.26	69.86	2.78
2	14.279	14.419	14.918	294.37	2510.88	100

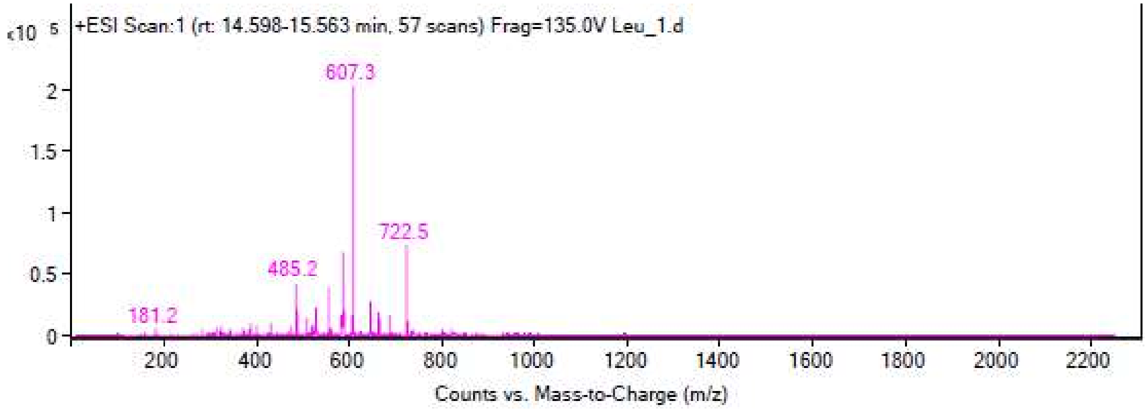

Peak List

m/z	z	Abund
485.2	1	42451.25
526.5	1	22780.45
554.5	1	38813.14
584.3		66515.44
585.3	1	68292.15
607.3	1	203895.69
608.3	1	68822.19
644.3	1	27674.21
722.5	1	73843.47
723.5	1	36041.13

Figure S37. HPLC-ESI spectra of compound **34.**

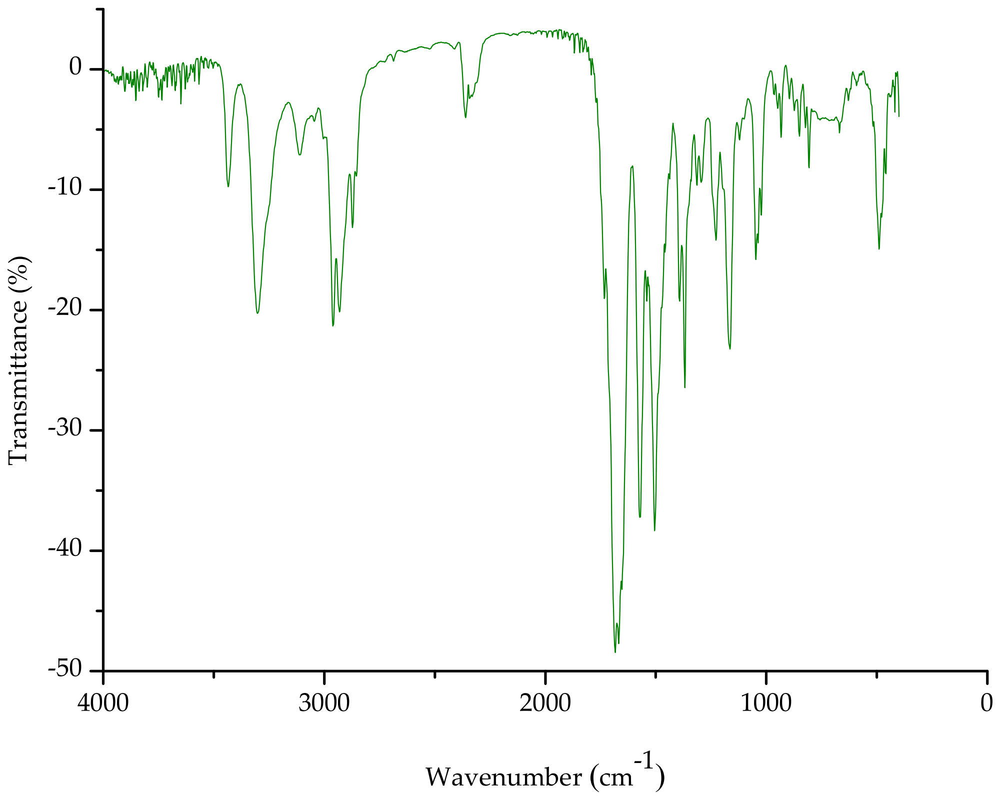

Figure S38. IR spectrum of compound **34** (c = 5 × 10^{-2} M) in DCM.

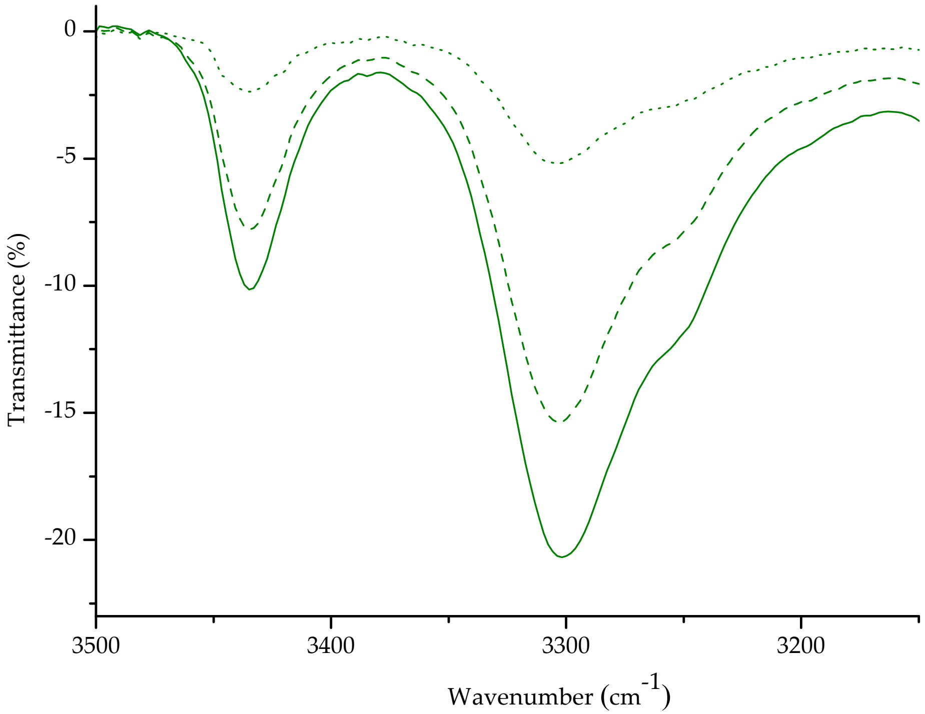

Figure S39. The NH stretching vibrations in concentration-dependent IR spectra of **34** in DCM [(—) $c = 5 \times 10^{-2}$ M, (——) $c = 2.5 \times 10^{-2}$ M, (· · ·) $c = 1.25 \times 10^{-2}$ M.

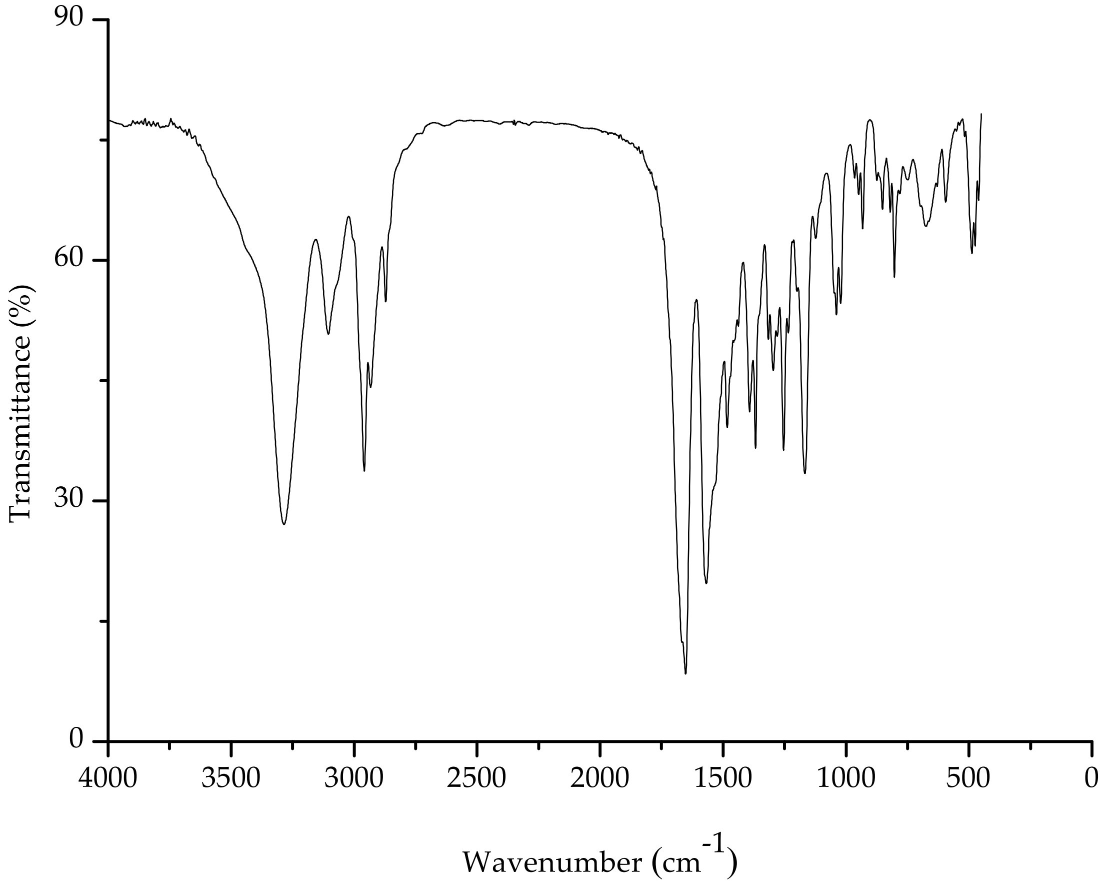

Figure S40. IR spectrum of compound **34** (2 mg) in KBr (200 mg).

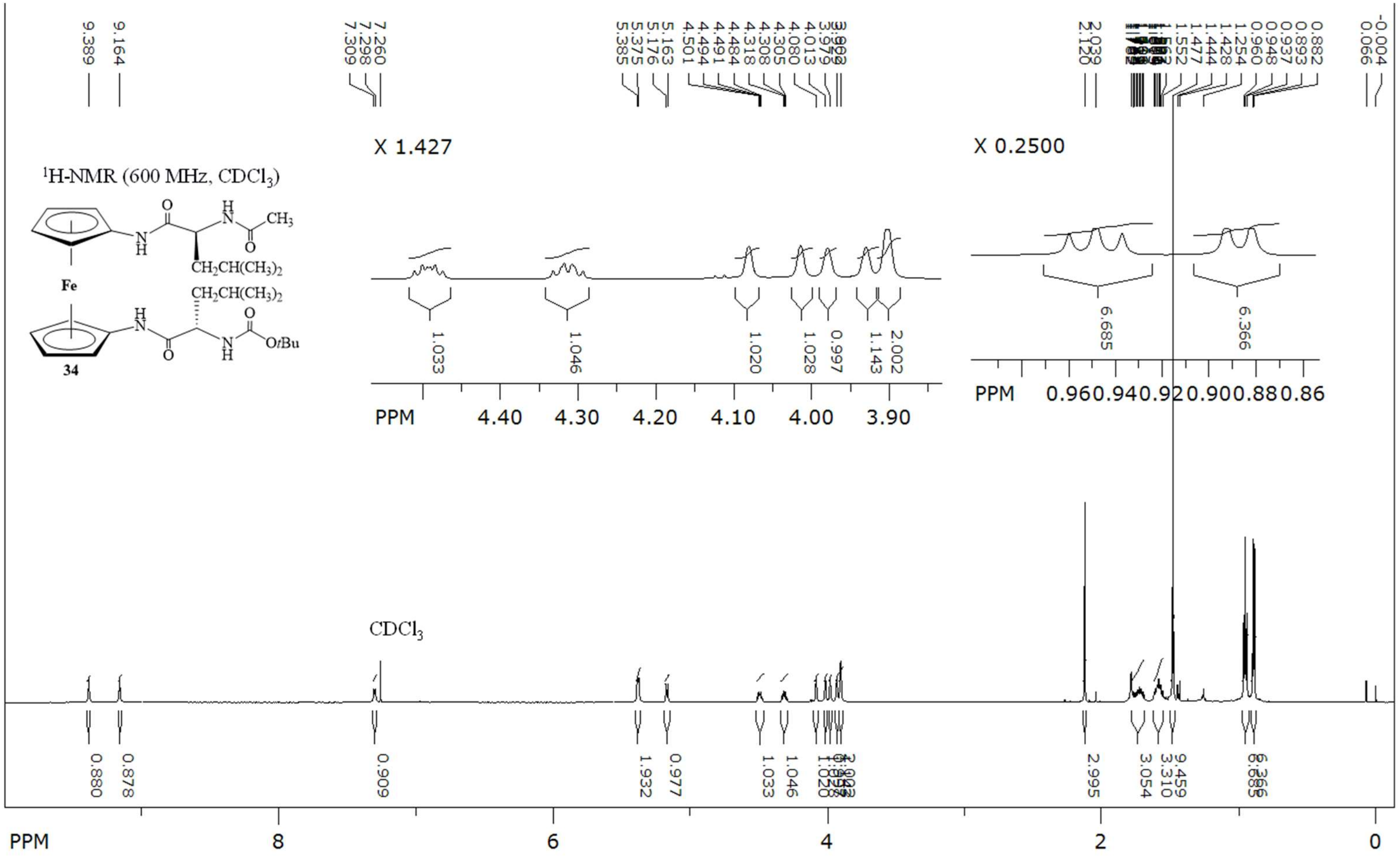

Figure S41. ^{1}H NMR spectrum of compound **34** ($c = 5 \times 10^{-2}$ M).

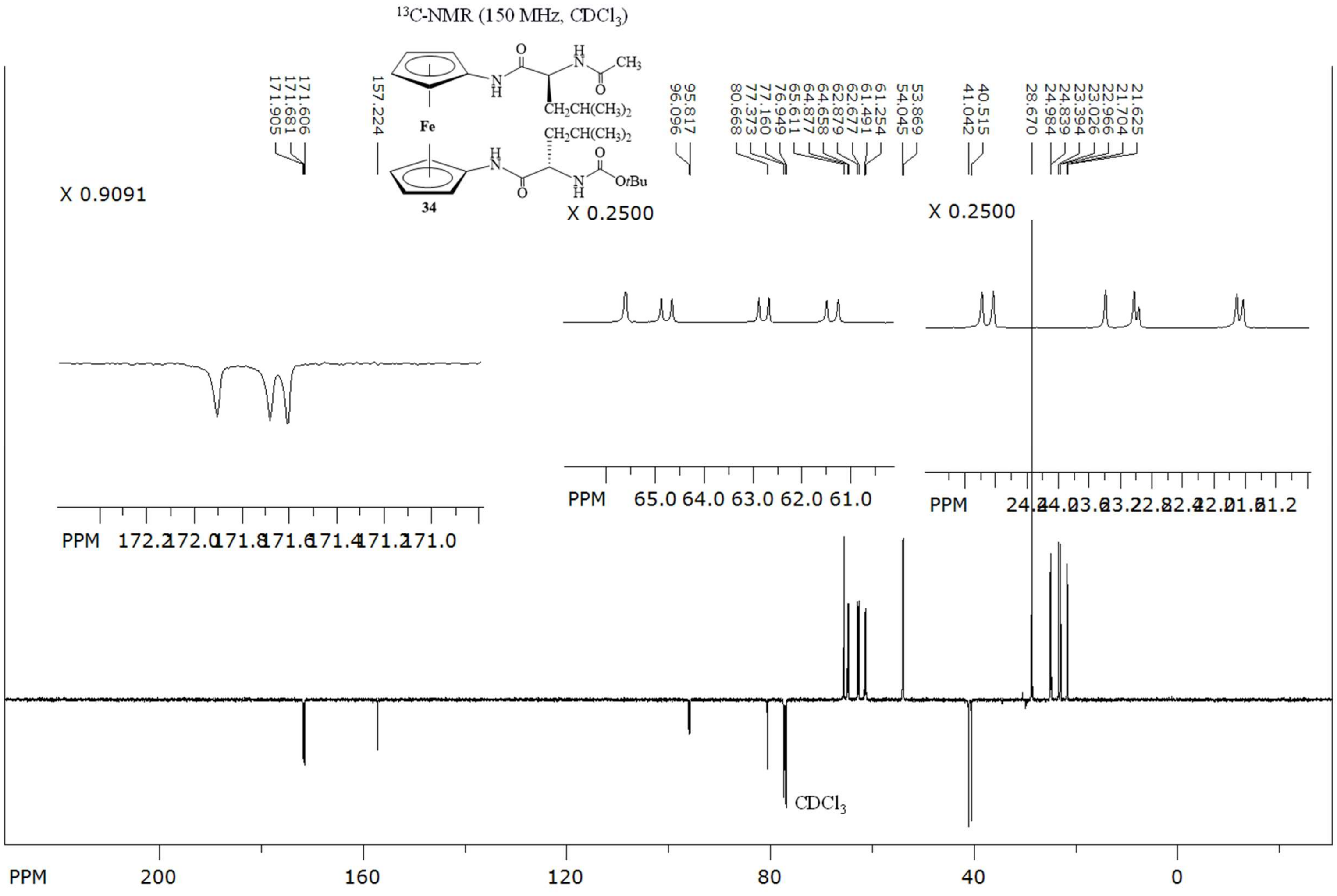

Figure S42. $^{13}C\{^{1}H\}$ NMR spectrum of compound **34** ($c = 5 \times 10^{-2}$ M).

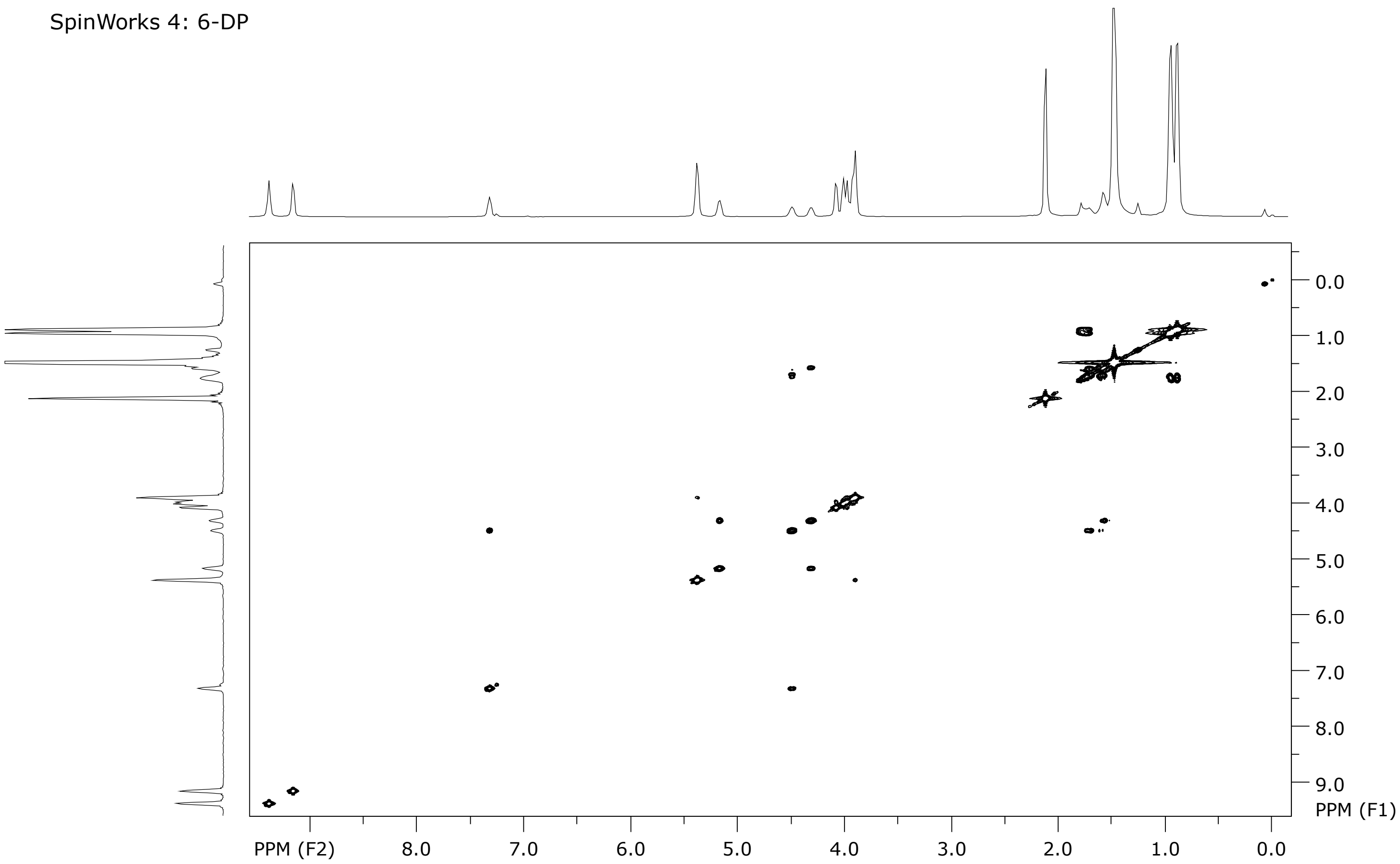

Figure S43. ^{1}H-^{1}H COSY NMR spectrum of compound **34** ($c = 5 \times 10^{-2}$ M).

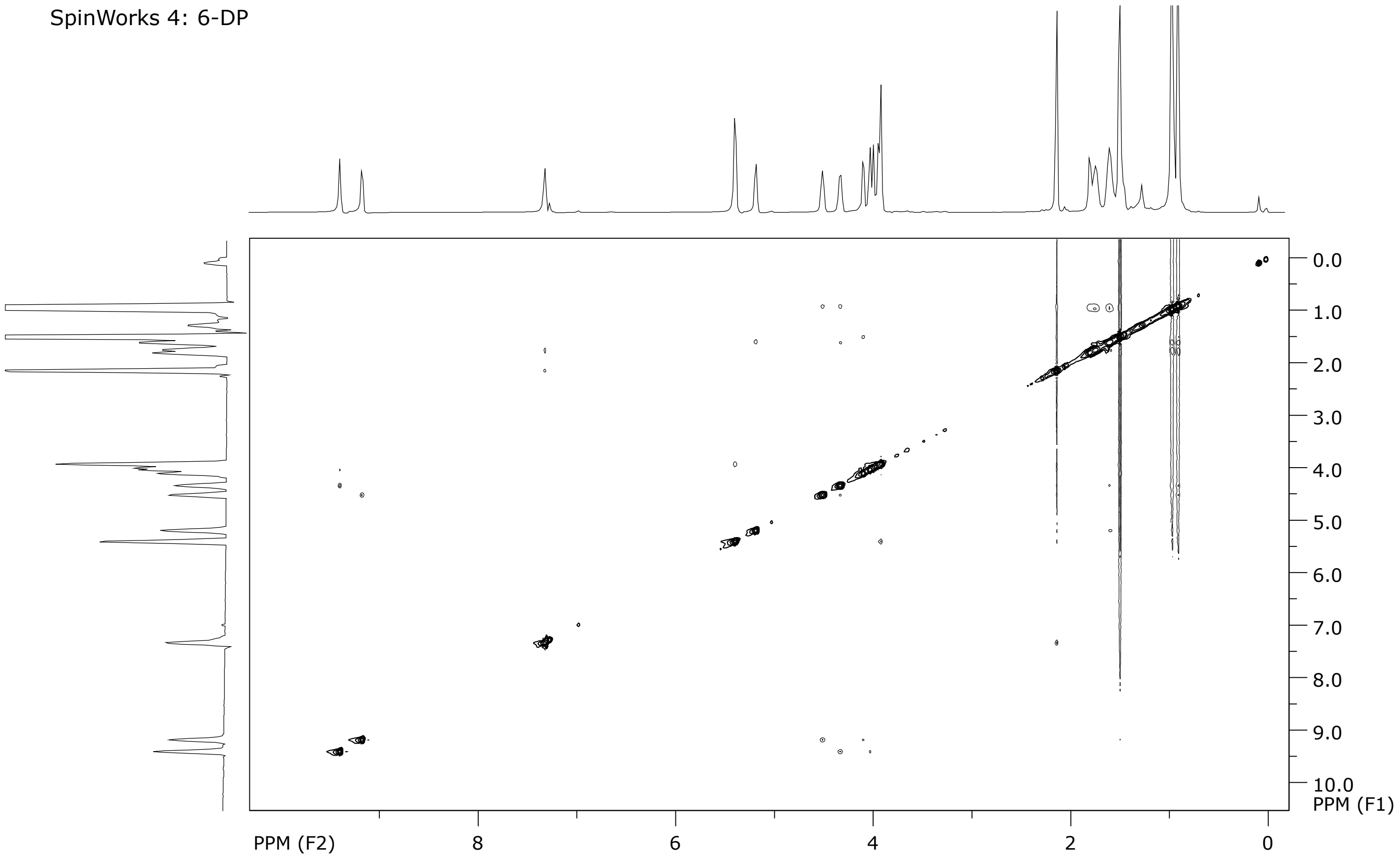

Figure S44. ^{1}H-^{1}H NOESY NMR spectrum of compound **34** ($c = 5 \times 10^{-2}$ M).

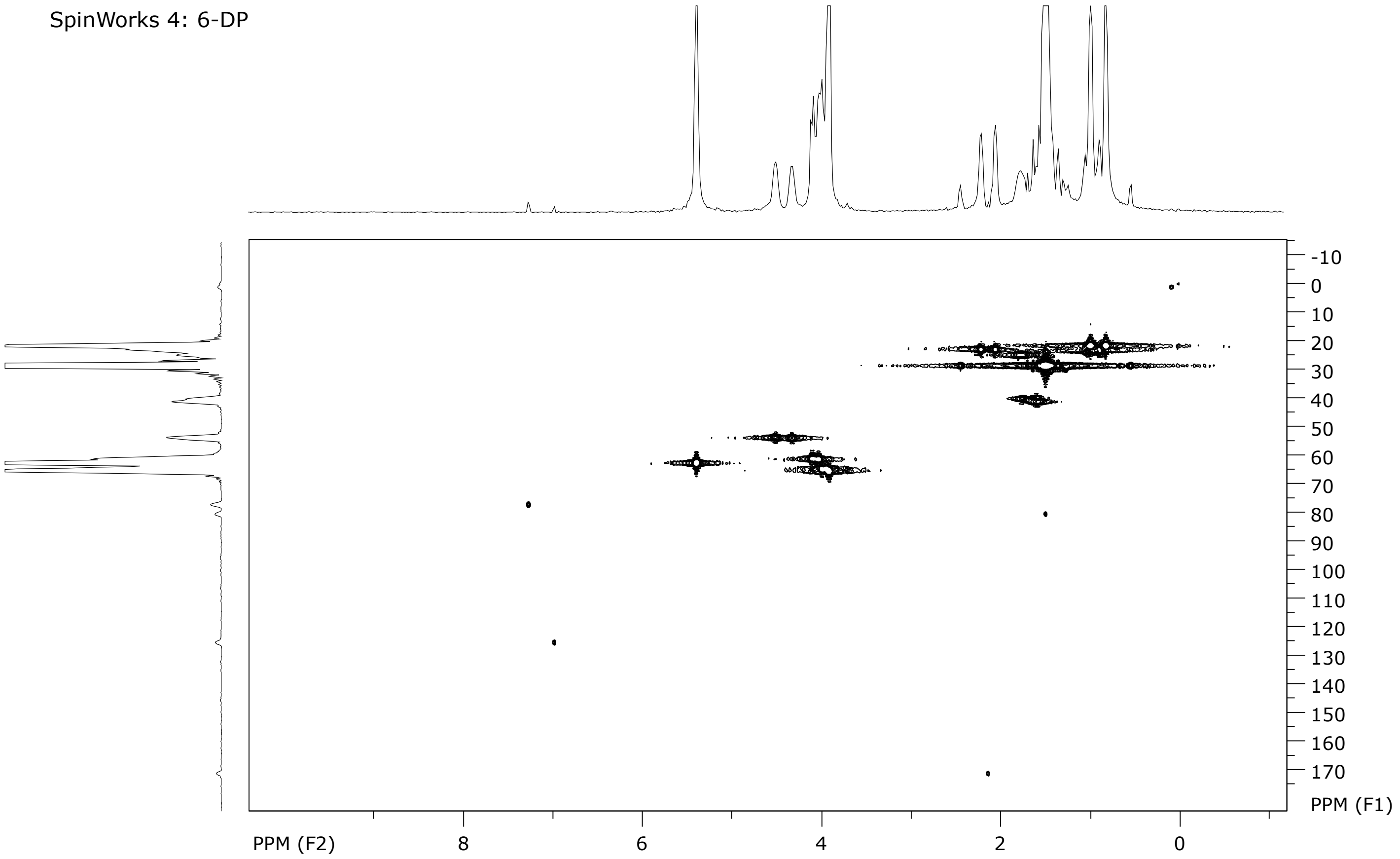

Figure S45. ^{1}H-^{13}C HMQC spectrum of compound **34** ($c = 5 \times 10^{-2}$ M).

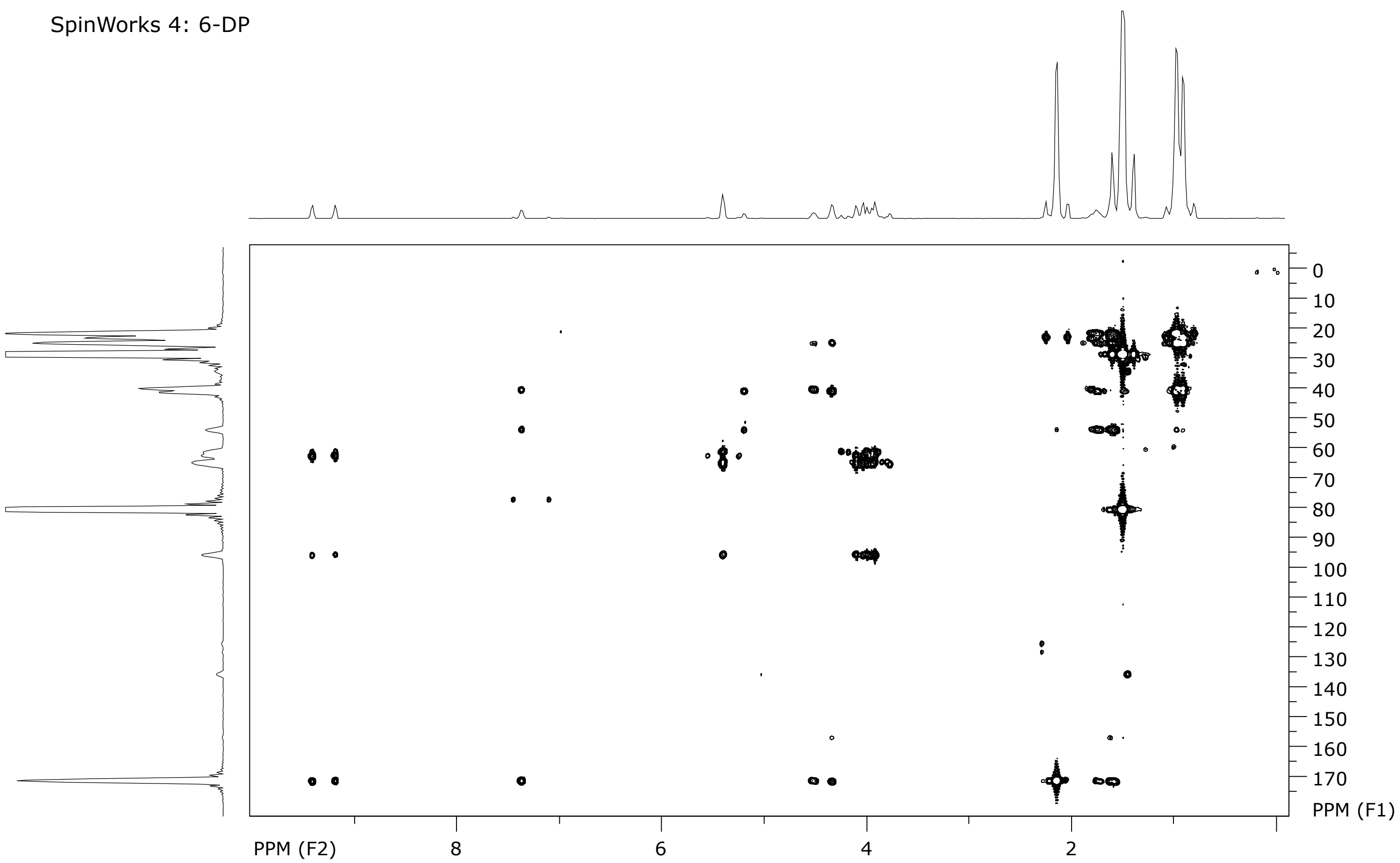

Figure S46. ^{1}H-^{13}C HMBC spectrum of compound **34** ($c = 5 \times 10^{-2}$ M).

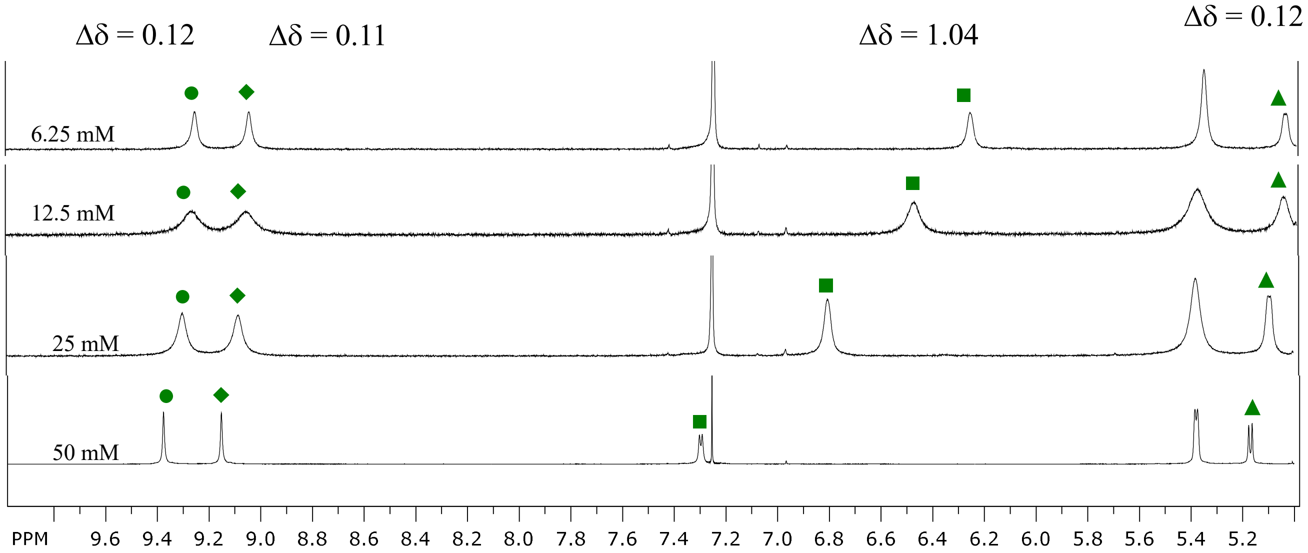

Figure S47. Concentration-dependent NH chemical shifts of compound **34** in $CDCl_3$.

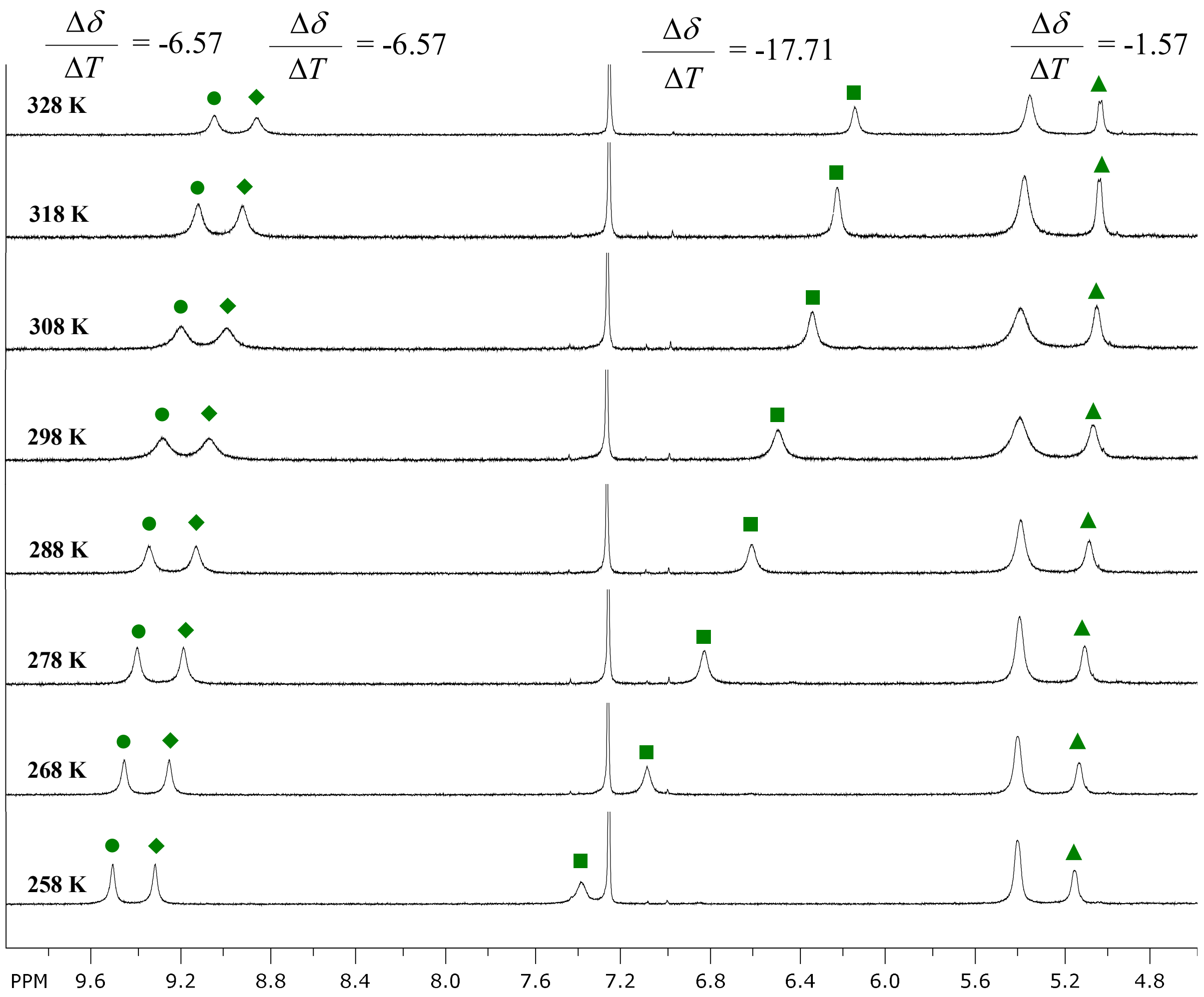

Figure S48. Temperature-dependent NH chemical shifts of compound **34** ($c = 1 \times 10^{-2}$ M).

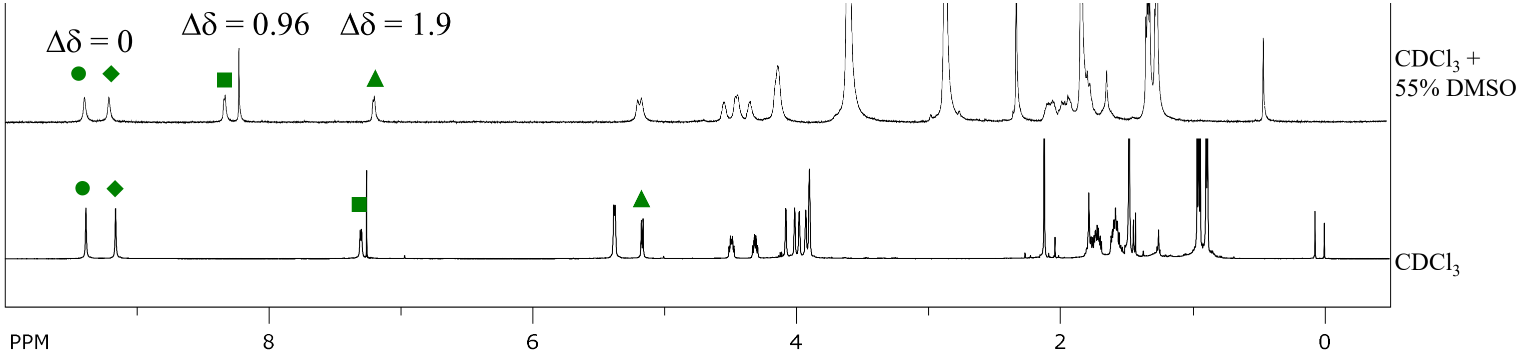

Figure S49. ^{1}H NMR spectra of compound **34** at varying concentrations of DMSO in $CDCl_3$
(c = 2.5 × 10-2 M).

Ac–D–Leu–NH–Fn–NH–D–Leu–Boc (37)

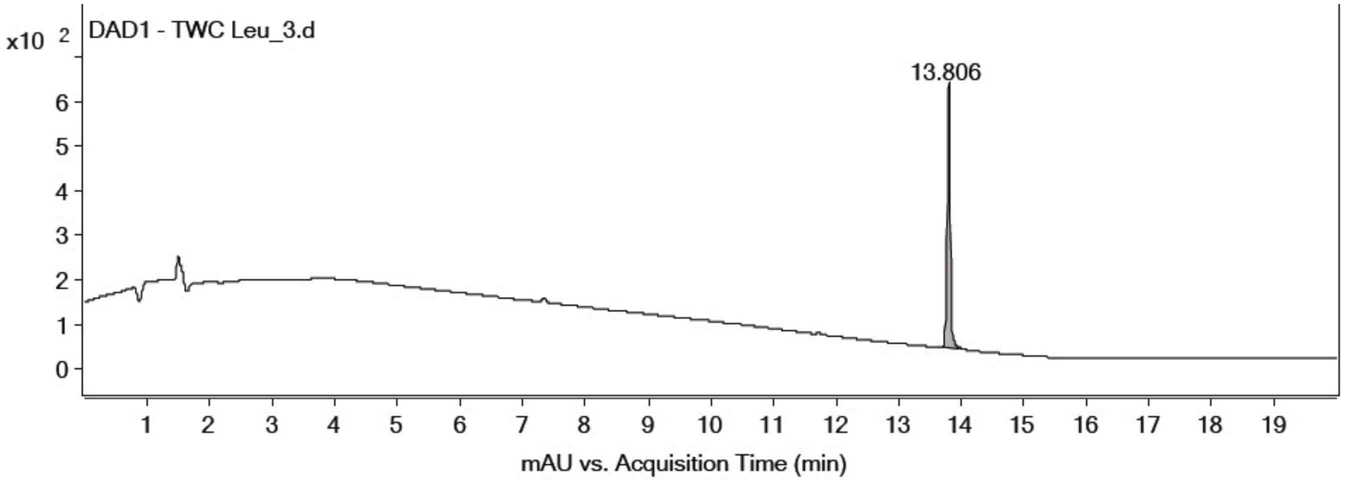

Integration Peak List

Peak	Start	RT	End	Height	Area	Area %
1	13.706	13.806	13.973	593.73	2177.34	100

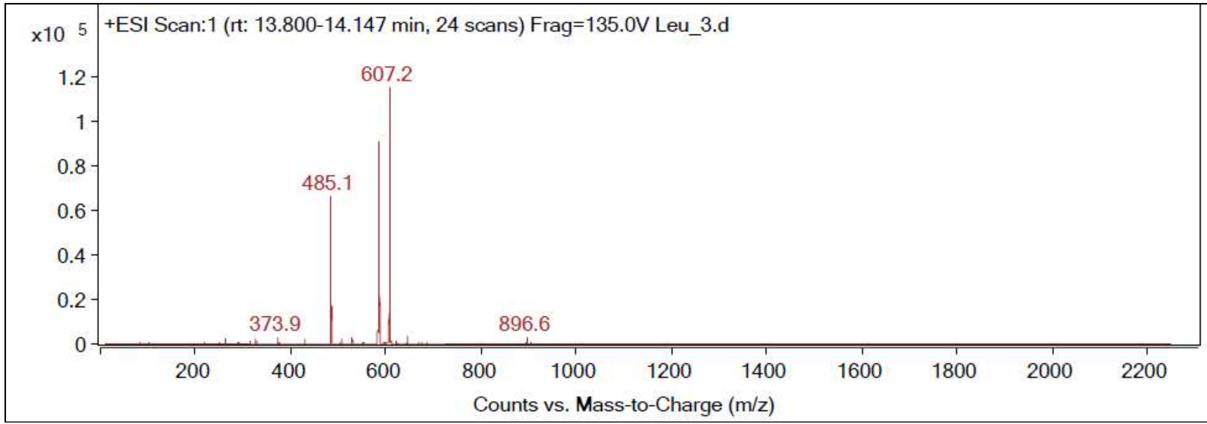

Peak List

m/z	z	Abund
485.1	1	66519.8
486.1	1	16861.59
583.1		6170.95
584.1		70845.26
585.1	1	90658.1
586.1	1	28849.23
605.1		8747.15
607.2	1	115051.34
608.1	1	40762.71
609.1	1	9568.72

Figure S50. HPLC-ESI spectra of compound **37.**

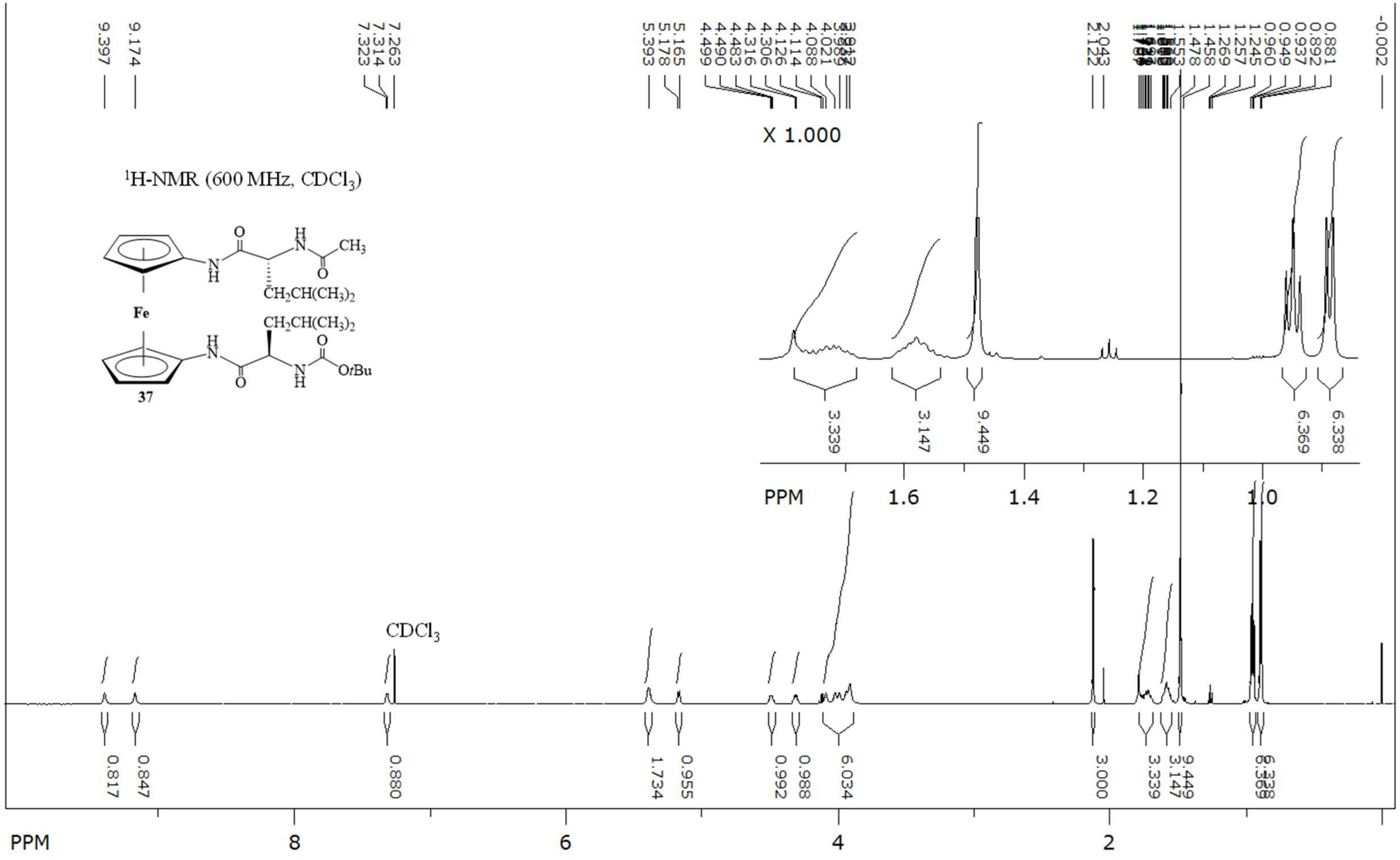

Figure S51. ^{1}H NMR spectrum of compound **37** ($c = 5 \times 10^{-2}$ M).

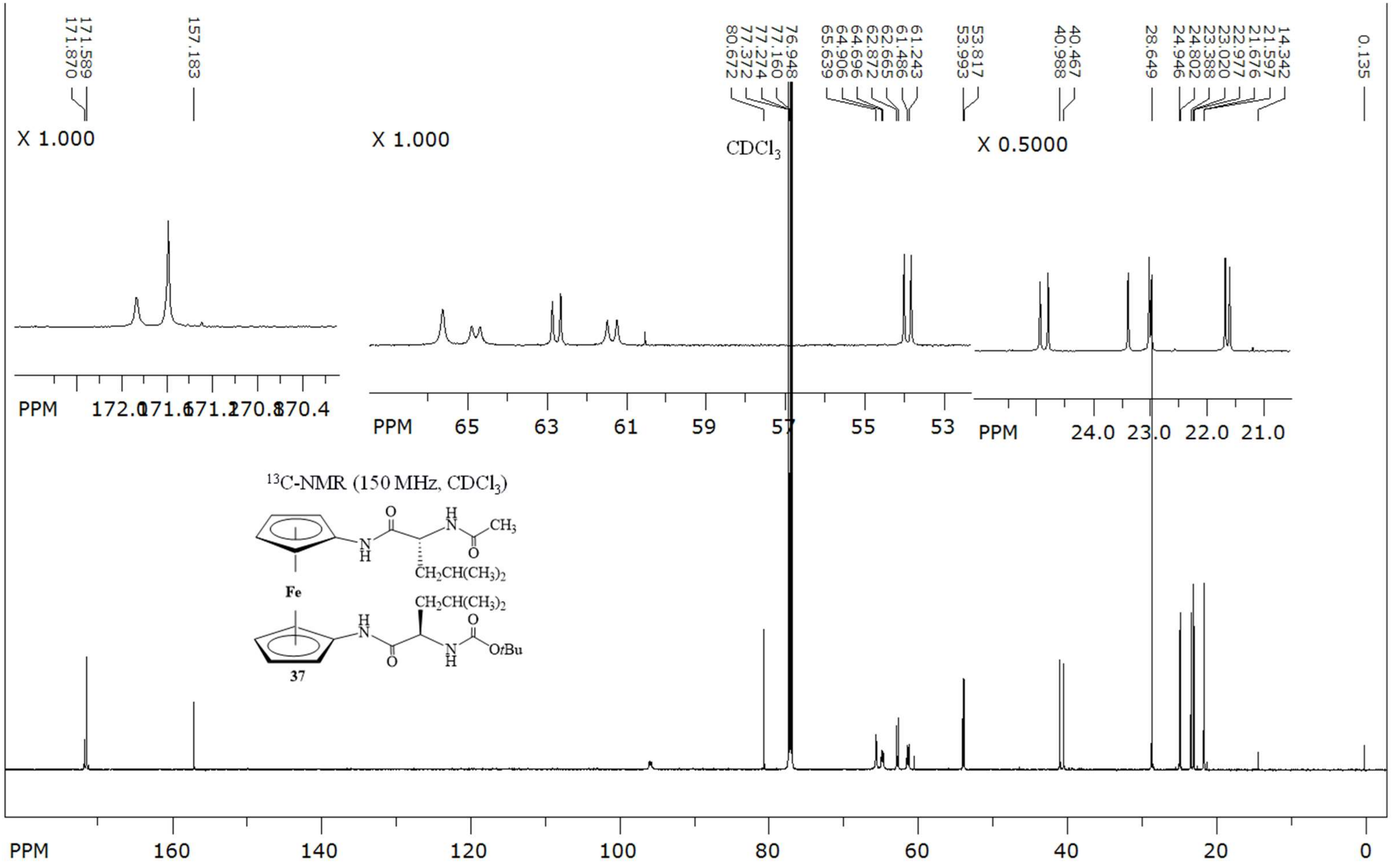

Figure S52. $^{13}C\{^1H\}$ NMR spectrum of compound **37** ($c = 5 \times 10^{-2}$ M).

Table S2. IR (ν in cm^{-1}) and NMR (δ in ppm) spectroscopic data of reference (Fn–NH–Ac and Fn–NH–Boc), model **(III)** and L-peptides **32–34.**

	ν (NH)		ν (CO)	δ			
	Free	Assoc.	Amide I	NH^{a}_{Fn} (●)	NH^{b}_{Fn} (◆)	NH_{Ac} (■)	NH_{Boc} (▲)
Fn–NH–Ac*	3436		1684			6.49	
Fn–NH–Boc*	3436		1723				5.55
Ac–L–Ala–NH^{a}–Fn–NH^{b}–L–Ala–Boc **(III)***	3439	3310<br>3253	1684<br>1665	9.02	9.06	6.85	5.21
Ac–L–Phe–NH^{a}–Fn–NH^{b}–L–Phe–Boc **(32)**	3430	3302<br>3266	1731<br>1706<br>1683<br>1668	9.15	9.21	7.21	5.43
Ac–L–Val–NH^{a}–Fn–NH^{b}–L–Val–Boc **(33)**	3434	3305<br>3249	1733<br>1716<br>1683<br>1666	9.21	9.04	6.75	5.21
Ac–L–Leu–NH^{a}–Fn–NH^{b}–L–Leu–Boc **(34)**	3434	3301<br>3253	1733<br>1684<br>1668	9.39	9.16	7.30	5.17

*Adapted from (19).

Table S3. Changes in chemical shifts (Δδ) observed for NH_{Fn} and NH_{Ac} in model **(III)** and L-peptides **32–34** at high (50 mM) vs. low concentrations (6.25 mM), from 258 – 328 K and at varying concentrations of DMSO in $CDCl_3$.

	NH^{a}_{Fn} (●)	NH^{b}_{Fn} (◆)	NH_{Ac} (■)	NH^{a}_{Fn} (●)	NH^{b}_{Fn} (◆)	NH_{Ac} (■)	NH^{a}_{Fn} (●)	NH^{b}_{Fn} (◆)	NH_{Ac} (■)	NH^{a}_{Fn} (●)	NH^{b}_{Fn} (◆)	NH_{Ac} (■)
	Concentration-dependent Δδ (ppm)			Temperature-dependent Δδ (ppm)			Temperature coefficients Δδ/ΔT (ppb K^{-1})			Solvent-dependent Δδ (ppm)		
Ac–L–Ala–NH^{a}–Fn–NH^{b}–L–Ala–Boc **(III)***	0.06	0.07	0.69	0.72	0.72	0.7	-10.33	-10.33	-10.0	0.13	0.11	1.71
Ac–L–Phe–NH^{a}–Fn–NH^{b}–L–Phe–Boc **(32)**	0.16	0.15	0.87	0.61	0.56	1.29	-8.0	-8.71	-18.42	0.07	0.16	1.44
Ac–L–Val–NH^{a}–Fn–NH^{b}–L–Val–Boc **(33)**	0.08	0.1	0.59	0.4	0.5	0.81	-7.14	-5.71	-11.57	0.15	0.11	1.29
Ac–L–Leu–NH^{a}–Fn–NH^{b}–L–Leu–Boc **(34)**	0.11	0.12	1.04	0.46	0.46	1.24	-6.57	-6.57	-17.71	0	0.08	0.96

*Adapted from (19).

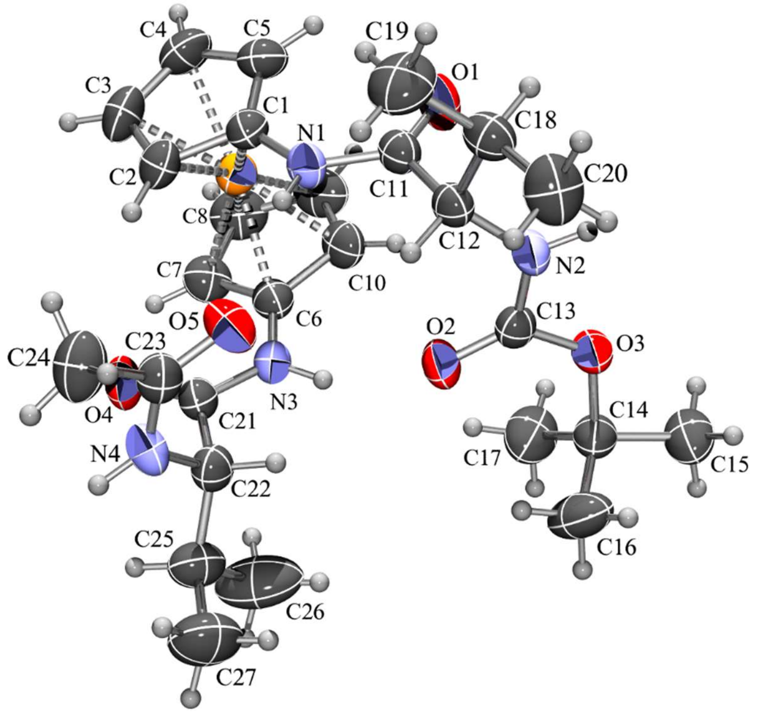

33

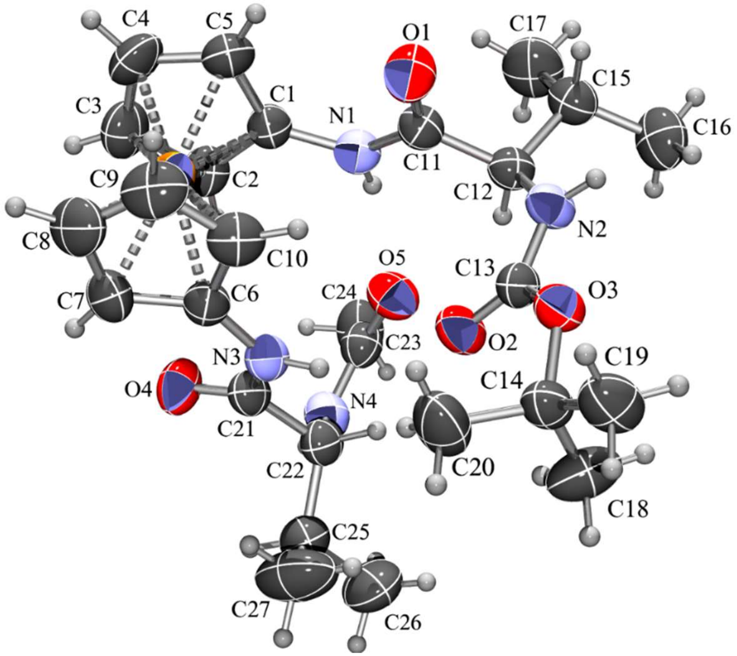

36

Figure S53. ORTEP-3 drawings of molecules of **33** and **36**. Displacement ellipsoids are drawn for the probability of 50 % and hydrogen atoms are shown as spheres of arbitrary radii.

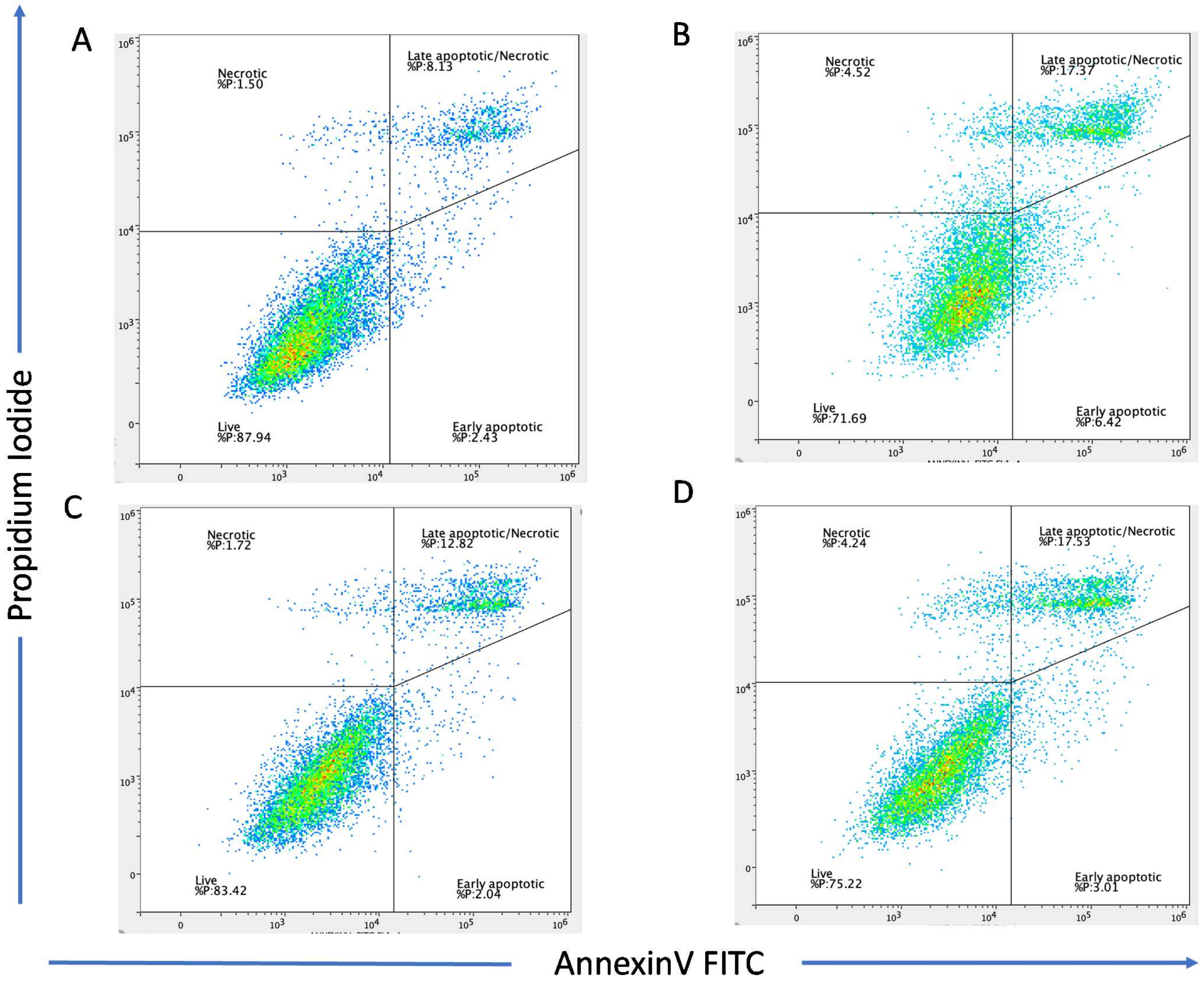

Figure S54. Detection of live, early apoptotic, late apoptotic/necrotic and necrotic HeLa cells by flow cytometry. A. Untreated cells (negative control), B. Starved cells (positive control), C. Cells treated with compound **37** (c = 26 μM), D. Cells treated with compound **37** (c = 105 μM).

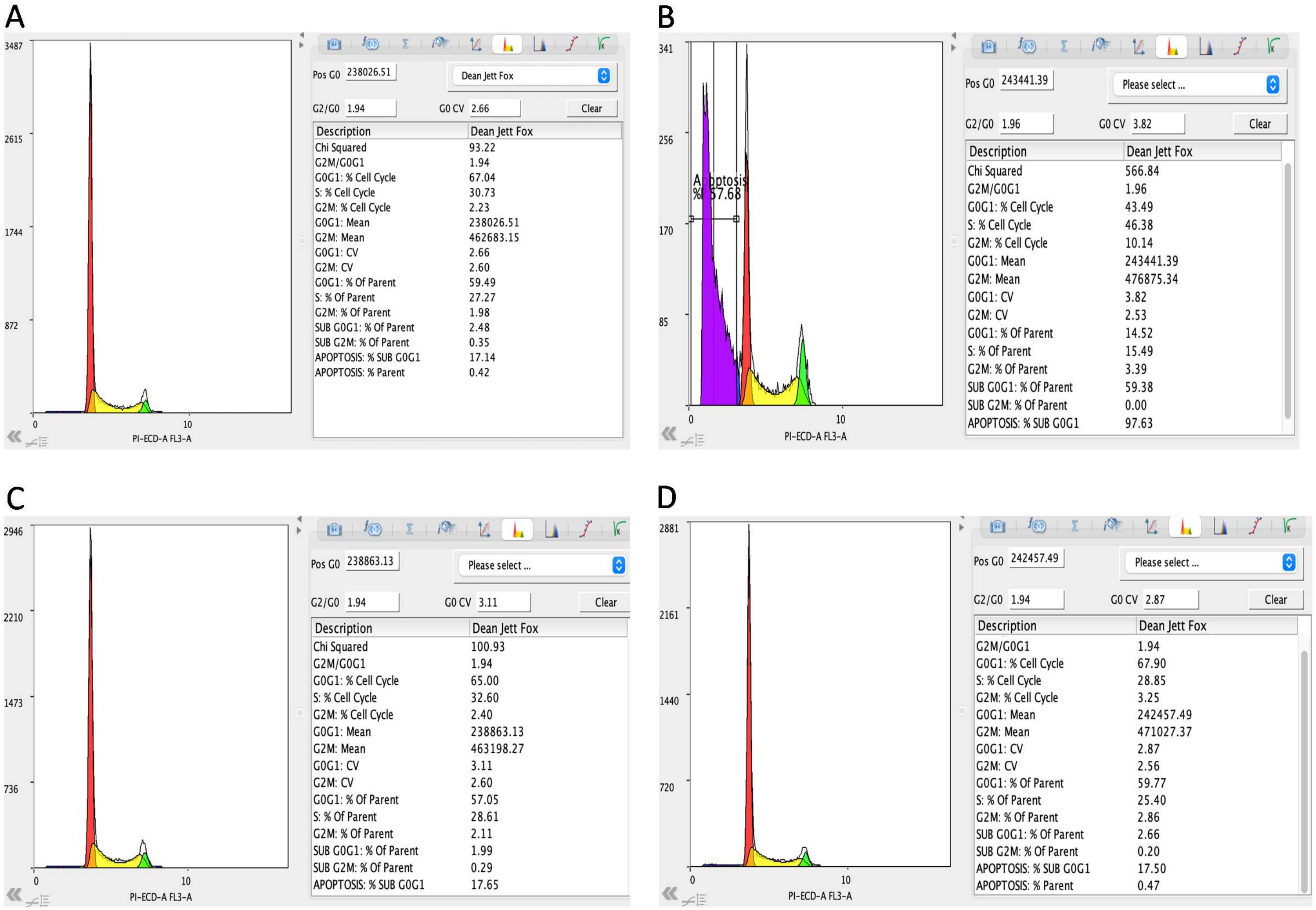

Figure S55. Detection of cell cycle phases by flow cytometry. A. Untreated cells (negative control), B. UV-treated cells (positive control), C. Cells treated with compound **37** (c = 26 μM), D. Cells treated with compound **37** (c = 105 μM).

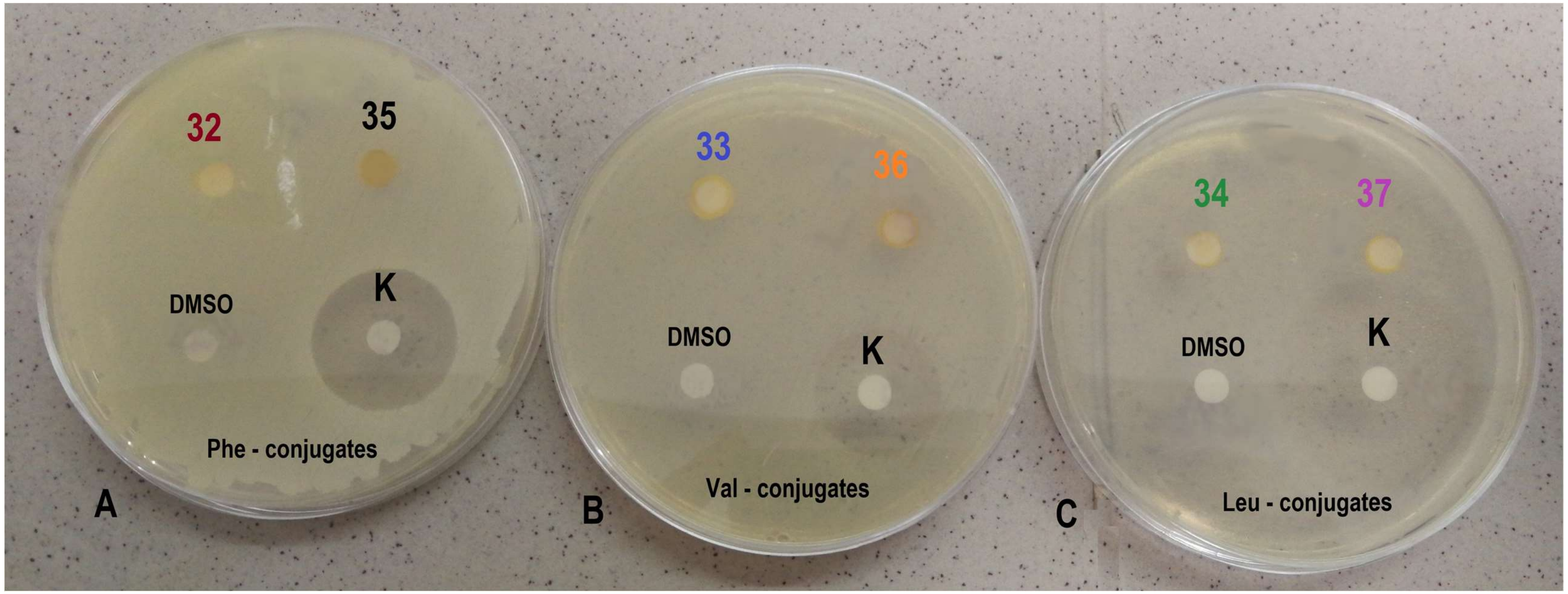

Figure S56. The absence of a growth inhibition zone of the tested microorganisms around the diagnostic disks with the ferrocene peptides **32–37** (A - *Staphylococcus aureus*; B - *Salmonella enterica s. Typhimurium*; C - *Escherichia coli*); DMSO 10 µL/disk - negative control; Kanamicyn 50 µg/disk - positive control).
